# A Multi-Strategy Enhanced Crested Porcupine Optimizer with Targeted Defense Mechanism Improvement for Global Optimization

**DOI:** 10.3390/biomimetics11090634

**Published:** 2026-09-04

**Authors:** Zhaoyong Fan, Xi Li, Zhenhua Xiao, Lianying Zou, Wangming Zhang

**Affiliations:** School of Information and Artificial Intelligence, Nanchang Institute of Science and Technology, Nanchang 330108, China

**Keywords:** crested porcupine optimizer, multi-strategy enhancement, chaotic initialization, dual-modal evasion, adaptive perturbation, engineering optimization

## Abstract

Metaheuristic algorithms are widely used to solve complex optimization problems, but the trade-off between exploration and exploitation often limits their performance. The Crested Porcupine Optimizer (CPO) employs four bio-inspired defense mechanisms and achieves competitive performance, but it still tends to converge prematurely, initialize populations poorly, and rely on static parameters that cannot adapt to different phases. This paper proposes a Multi-Strategy Enhanced Crested Porcupine Optimizer (MSCPO) with four phase-targeted enhancement strategies: (1) Kent Chaos Opposition-Based Learning Initialization (KCOL) improves the initial population distribution through chaotic-weighted reflection; (2) Arctic Puffin Optimization (APO)-Inspired Dual-Modal Evasion (APO-DME) reduces dependence on the global best solution and increases population diversity through dual-mode differential perturbation; (3) Adaptive Dual-Differential Perturbation (ADDP) refines the search by combining population diversity and elite guidance information; (4) Periodic Dynamic Adaptive Perturbation (PDAP) enhances exploitation through periodic trigonometric perturbation and a fitness-conditioned update rule. These strategies interact across the optimization process to strengthen each defense mechanism at the appropriate phase. On the CEC 2017 and CEC 2022 benchmark suites, MSCPO achieves the best overall mean rank among all compared algorithms, with an overall rank of 2.638 on CEC 2017 and 2.375 on CEC 2022. A full-factorial ablation over all 16 strategy combinations confirms that each strategy contributes positively: removing any single strategy degrades the overall mean rank, and the complete MSCPO achieves the best mean rank (3.34), significantly outperforming all single-strategy variants (Wilcoxon signed-rank test, *p* < 0.001). To verify the practical applicability of MSCPO, the algorithm is further applied to three engineering design problems: step-cone pulley design, hydrostatic thrust bearing design, and robotic gripper design. MSCPO ranks first on the hydrostatic thrust bearing problem, second on the step-cone pulley problem, and third on the robotic gripper problem. Future work will explore adaptive population sizing to further improve the scalability of MSCPO on very high-dimensional problems.

## 1. Introduction

Optimization is a fundamental process in science and engineering, with applications ranging from structural design [1] and resource allocation [2] to feature selection [3], hyperparameter tuning [4] and signal decomposition [5]. The core difficulty lies in finding the global optimum within a high-dimensional, multimodal, and non-convex search space [6]. Nature-inspired metaheuristic algorithms have become a popular approach to these problems because they require no derivatives, need few parameters, and can navigate complex landscapes without problem-specific mathematical models [7]. Drawing on biological evolution, physical phenomena, and swarm intelligence, these algorithms can handle non-differentiable or discontinuous objectives that are often intractable for gradient-based methods.

In 2024, Abdel-Basset et al. [8] proposed the Crested Porcupine Optimizer (CPO), inspired by the defensive behaviors of the crested porcupine. CPO models four defense mechanisms of increasing aggressiveness: visual intimidation (first defense), auditory deterrence (second defense), olfactory assault (third defense), and physical counterattack (fourth defense). The first two mechanisms serve as exploration operators that drive broad dispersive search across the solution space, while the latter two function as exploitation operators for intensive local refinement around promising regions [8]. CPO employs a cyclic population reduction technique that adjusts the number of active individuals in each iteration, reflecting that not all porcupines perceive threats simultaneously. This design preserves population diversity while still permitting convergence. CPO has performed well on CEC 2014, CEC 2017, CEC 2020, and CEC 2022 benchmark suites. On these suites, it outperforms classical metaheuristics such as the gray wolf optimizer (GWO) [9], whale optimization algorithm (WOA) [10], differential evolution (DE) [11], and salp swarm algorithm [12], as well as more recent algorithms such as gradient-based optimizer (GBO) [13], African vultures optimization algorithm (AVOA) [14], Runge–Kutta method (RUN) beyond the metaphor [15], equilibrium optimizer (EO) [16], and artificial gorilla troops optimizer (GTO) [17].

Since 2024, CPO has been applied to a growing range of engineering problems. Zhao et al. [18] employed an enhanced CPO variant for wireless sensor network deployment and achieved higher coverage rates than competing methods. Pan et al. [19] combined CPO with feature mode decomposition for bearing fault diagnosis and extracted fault characteristic frequencies reliably even under strong interference noise. Zhang et al. [20] improved CPO for unmanned aerial vehicle (UAV) path planning by incorporating the Cauchy inverse cumulative distribution and JAYA attack strategies. Cai et al. [21] optimized PID controller parameters for CO_2_ control in cleanrooms with an improved CPO. Luo et al. [22] applied a self-adaptive CPO variant to optimize ensemble learning models for concrete chloride concentration prediction. These applications demonstrate that CPO can be adapted to a wide range of problem domains.

Despite its strengths, CPO has several limitations. First, its standard random initialization can produce clustered or poorly distributed populations, leaving promising regions unexplored. Second, the first defense depends on a single global best solution, which risks premature convergence on multimodal landscapes. Third, the second defense employs fixed random weights without adaptation, so its exploration effectiveness varies across optimization phases. Fourth, the static convergence parameter α in the fourth defense cannot adapt to the evolving search landscape; it either exploits too little in later stages or perturbs too much, disrupting convergence. Several improved variants of CPO have been proposed to address these limitations; a mechanism-level comparison of these variants is provided in Section 6.2. However, these variants integrate multiple enhancements without establishing an explicit correspondence between each strategy and the intrinsic phases of the original CPO.

To fill this gap, this paper proposes a Multi-Strategy Enhanced Crested Porcupine Optimizer (MSCPO) that integrates four phase-targeted enhancement strategies, each targeting one weakness identified above: (1) Kent Chaos Opposition-Based Learning (KCOL) improves standard random initialization with a Kent chaotic map and opposition-based learning. The Kent map produces sequences with favorable ergodicity and near-uniform distribution properties [23,24]; the opposition-based reflection constructs complementary candidates with respect to the current population bounds, from which the better candidate is greedily retained. (2) APO-Inspired Dual-Modal Evasion (APO-DME) redesigns the first defense mechanism. Inspired by the Arctic Puffin Optimizer [25], APO-DME eliminates the single-point dependency on the global best and introduces bimodal differential perturbation: uniform random perturbation based on population differential information for local diversity, and Levy flight for long-distance global escapes. The two modes are chosen with equal probability. (3) Adaptive Dual-Differential Perturbation (ADDP) enhances the second defense by combining two different vectors, one defined by two random population members and one pointing toward the current best solution, both scaled by a random coefficient. (4) Periodic Dynamic Adaptive Perturbation (PDAP) modifies the fourth defense by adding a periodic trigonometric perturbation and a random-peer reference, where the update direction is conditioned on the relative fitness of the current individual and the reference peer.

MSCPO is evaluated on CEC 2017 (D = 10, 30, 50, and 100) and CEC 2022 (D = 10, 20) against 11 competitors: genetic algorithm (GA) [26], DE [11], WOA [10], Harris Hawks Optimization (HHO) [27], dung beetle optimizer (DBO) [28], Gekko Japonicus Algorithm (GJA) [29], sparrow search algorithm (SSA) [30], Beaver Behavior Optimizer (BBO) [31], the original CPO [8], and two high-performance CEC competition algorithms (L-SHADE [32], the CEC 2014 winner, and LSHADE-SPACMA [33], a top-ranked CEC 2017 entrant). Statistical analysis employs the Wilcoxon signed-rank test and the Friedman mean rank test. The ablation study covers all strategy combinations in a full-factorial design and investigates the individual and combined contributions of each strategy with Nemenyi post hoc analysis [34]. MSCPO is also validated on three engineering design problems: step-cone pulley design, hydrostatic thrust bearing design, and robotic gripper design [35]. The main contributions of this paper are summarized as follows:

1. MSCPO achieves an overall Mean Rank of 2.638 on CEC 2017 and 2.375 on CEC 2022, surpassing the original CPO (ranks of 5 and 4, respectively) and most nature-inspired competitors at D ≤ 50.
2. The full-factorial ablation shows that the four strategies provide complementary, phase-targeted contributions: removing any single strategy degrades the overall mean rank, the two exploration-phase strategies (APO-DME and ADDP) carry the largest individual contribution, and the complete MSCPO achieves the best mean rank (3.34), significantly outperforming all single-strategy and pairwise variants.
3. MSCPO outperforms CPO and most competitors on moderate-to-high dimensional problems (D = 10 to D = 50), although its advantage over the strongest rivals (GJA, BBO, and LSHADE-SPACMA) weakens at D = 100, highlighting the difficulty of budget-constrained high-dimensional optimization.
4. MSCPO also achieves highly competitive results on three engineering design problems, demonstrating that it remains effective on real-world problems beyond benchmark suites.

The remainder of this paper is organized as follows. Section 2 reviews the original CPO algorithm. Section 3 describes the MSCPO algorithm and the four improvement strategies. Section 4 reports the experimental results on the benchmark suites. Section 5 covers the engineering applications. Section 6 discusses the practical efficiency of MSCPO, compares it with prior CPO variants, and analyzes its limitations; Section 7 concludes the paper and outlines future work.

## 2. Original CPO Algorithm

The Crested Porcupine Optimizer (CPO) [8] is a novel metaheuristic algorithm inspired by the biological defense mechanisms of crested porcupines (CP). It mimics four defensive behaviors of CP: visual intimidation, auditory deterrence, olfactory assault, and physical attack. The first two (sight and sound) are responsible for exploration, and the latter two (odor and physical attack) govern exploitation. This division balances global and local search.

### 2.1. Initialization

The initial population of CPO is generated uniformly within the boundaries of the search space:

(1) $$x_{i}=L+r\cdot (U-L)\;,\;i=1,\;2,\cdots ,N^{\prime }$$

where r is a vector of uniformly distributed random numbers in $\left[0,\;1\right]$, and *U* and *L* are the upper and lower bounds of the search range.

### 2.2. Cyclic Population Reduction

CPO starts with a randomly initialized population. In each iteration, it employs cyclic population reduction. This mechanism reflects the fact that not all individuals in a group perceive a threat simultaneously. Mathematically, it dynamically adjusts the number of active individuals N participating in position updates during each iteration t, as defined by the following equation:

(2) $$N=N_{min}+\left(N^{\prime }-N_{min}\right)\cdot \left(1-\left(\frac{t\;\%\;\frac{T_{max}}{T}}{\frac{T_{max}}{T}}\right)\right)$$

where $N_{min}$ is the minimum population size, t is the current iteration, % represents the modulus operator, $T_{max}$ is the maximum number of iterations, and T is a cyclic parameter typically set to 2. Each active individual then selects a strategy based on a preset probability model to update its position, and the better solution is retained through greedy selection.

### 2.3. Exploration Phase

#### 2.3.1. First Defense Mechanism (Sight)

When a porcupine sights a predator from a distance, it adopts a cautious approach combining its own position with a reference midpoint, enabling wide-range exploration. The mathematical model is as follows:

(3) $$x_{i}^{t+1}=x_{i}^{t}+\tau_{1}\cdot \left|2\tau_{2}x_{CP}^{t}-y_{i}^{t}\right|$$

where the reference midpoint $y_{i}^{t}$ is defined as:

(4) $$y_{i}^{t}=\frac{x_{i}^{t}+x_{r}^{t}}{2}$$

Here, $\tau_{1}∼N\left(0,1\right)$ (standard normal distribution), $\tau_{2}\;∼U\left(0,1\right)$, $x_{CP}^{t}$ is the current global best solution, and $x_{r}^{t}$ is a randomly selected individual from the population (r≠i).

In the visual defense mechanism, each individual generates a midpoint between itself and a random peer and then perturbs this midpoint, guided by the crest porcupine’s position. The absolute-value operator introduces directional diversity.

#### 2.3.2. Second Defense Mechanism (Sound)

As the predator draws closer, the porcupine emits acoustic warnings. This mechanism blends individual memory with neighborhood information, facilitating local exploitation and medium-range exploration. The position update is as follows:

(5) $$x_{i}^{t+1}=\left(1-U_{1}\right)x_{i}^{t}+U_{1}\left(y^{t}+\tau_{3}\left(x_{r1}^{t}-x_{r2}^{t}\right)\right)$$

where $U_{1}\;$ is a random value within $\left[0,\;1\right]$, $\tau_{3}∼U\left(0,1\right)$, and $x_{r1}^{t}$ and $x_{r2}^{t}$ are two distinct random individuals $\left(r1\neq r2\neq i\right)$.

This mechanism produces a convex combination of the current position and a perturbed reference point. The difference vector $\left(x_{r1}^{t}-x_{r2}^{t}\right)$ introduces differential evolution-style perturbation, promoting population diversity.

### 2.4. Exploitation Phase

#### 2.4.1. Third Defense Mechanism (Odor)

When the predator is very close, the porcupine releases a pungent odor. The defense intensity correlates with the perceived threat level through an adaptive sensory factor. The mathematical model is as follows:

(6) $$x_{i}^{t+1}=\left(1-U_{1}\right)x_{i}^{t}+U_{1}\left(x_{r1}^{t}+\;S_{t}\left(x_{r1}^{t}-x_{r2}^{t}\right)-\tau_{3}\delta \gamma_{t}S_{t}\right)$$

(7) $$\delta =\left\{\begin{array}{l} +1,\;\;\;if\;rand\leq 0.5\\ -1,\;\;\;otherwise \end{array}\right.$$

(8) $$\gamma_{t}=2\cdot rand\cdot {\left(1-\frac{t}{t_{max}}\right)}^{\frac{t}{t_{max}}}$$

(9) $$S_{t}=exp\left(f\left(x_{i}^{t}\right)/\sum_{k=1}^{N}f\left(x_{k}^{t}\right)+\varepsilon \right)$$

where $\tau_{3}$ is a random value uniformly distributed in [0, 1], δ is the direction parameter, $\gamma_{t}\;$ is the defense factor, $S_{t}$ is the odor diffusion factor (constrained to the interval [0.3, 2.6]), ε is a small constant to avoid division by zero, and the exponential mapping ensures $S_{t}>0$.

#### 2.4.2. Fourth Defense Mechanism (Physical Attack)

As a last resort, the porcupine charges physically toward the predator. This mechanism concentrates the search around the best-known solution (the crest porcupine). The mathematical model is as follows:

(10) $$x_{i}^{t+1}=x_{CP}^{t}+\left(\alpha \left(1-\tau_{4}\right)+\tau_{4}\right)\left(\delta x_{CP}^{t}-x_{i}^{t}\right)-\tau_{5}\delta \gamma_{t}F_{i}^{t}$$

where $x_{CP}^{t}\;$ is the best solution obtained, α=0.1 is the convergence acceleration factor, $\tau_{4},\;\tau_{5}∼U\left(0,1\right)$, and $F_{i}^{t}$ is the average impact force on the *i*-th predator.

(11) $$F_{i}^{t}=\tau_{6}\times \frac{m_{i}\left(v_{i}^{t+1}-v_{i}^{t}\right)}{∆t}$$

where $\tau_{6}∼U\left(0,1\right)$, $m_{i}$ is the mass of the predator, $v_{i}^{t}=x_{i}^{t}$ is the initial speed, $v_{i}^{t+1}=x_{r}^{t}$ is the final speed of a random solution from the population, and ∆t is the current iteration number.

After each position update, the newly generated position is evaluated, and a greedy selection step determines whether it is retained. Each crested porcupine maintains a historical best position $x_{p}^{i}$, initialized to its starting position. If $f\left(x_{i}^{t+1}\right)\leq f\left(x_{p}^{i}\right)$, the new position is accepted and $x_{p}^{i}\leftarrow x_{i}^{t+1}$; otherwise, $x_{i}^{t+1}$ is discarded and reverted to $x_{p}^{i}$. Whenever an individual improves upon the global best, the global best is updated accordingly.

## 3. Multi-Strategy Enhanced Crested Porcupine Optimizer

Although CPO demonstrates competitive performance, subsequent research has shown that, like many population-based algorithms, it suffers from slow convergence, low accuracy, and entrapment in local optima [18,19,36,37,38]. This section presents the Multi-strategy Enhanced Crested Porcupine Optimizer (MSCPO). MSCPO introduces four strategies: Kent Chaos Opposition-Based Learning initialization (KCOL); APO-inspired Dual-Mode Evasion (APO-DME); Adaptive Dual-Differential Perturbation (ADDP); and Periodic Dynamic Adaptive Perturbation (PDAP). Each modification targets a specific weakness in the corresponding CPO defense phase.

### 3.1. Targeted Enhancement Framework of MSCPO

The correspondence between the original CPO defense mechanisms and the proposed enhancement strategies is summarized in Table 1. Three phases receive new strategies; the olfactory phase remains unchanged. Each enhancement strategy is designed to address specific weaknesses in the corresponding CPO defense phase.

As shown in Table 1, the third defense mechanism remains intact. The sensory factor $S_{t}$ of the olfactory defense is computed from the fitness values of the entire population, so it already injects population-level fitness information into the exploitation phase; we therefore retain this mechanism unchanged and concentrate the modifications on the remaining phases. KCOL runs before the main loop, providing the initial population for the subsequent search. APO-DME and ADDP modify the two exploration-phase defense mechanisms, while PDAP modifies the fourth defense mechanism in the exploitation phase through periodic trigonometric perturbation and a fitness-conditioned position update.

### 3.2. Kent Chaos Opposition-Based Learning Initialization (KCOL)

The initial population of a metaheuristic strongly influences whether it can find global optima. A well-distributed initial population increases the probability that at least one individual starts within the basin of attraction of the global optimum, which can substantially reduce the required search effort.

The original CPO uses uniform random initialization in Equation (1). An initial population with poor coverage reduces the probability of starting near the global optimum. KCOL replaces this scheme with Kent chaotic mapping and opposition-based learning (OBL).

The Kent chaotic map is a piecewise linear transformation on the interval [0, 1], defined as:

(12) $$K_{i,j}^{\left(m+1\right)}=\left\{\begin{array}{ll} \frac{K_{i,j}^{\left(m\right)}}{r}, & 0\leq K_{i,j}^{\left(m\right)}\leq r\\ \frac{1-K_{i,j}^{\left(m\right)}}{1-r}, & r<K_{i,j}^{\left(m\right)}\leq 1 \end{array}\right.$$

where $K_{i,j}^{\left(m\right)}\;$ denotes the *m*-th chaotic iterate for the *j*-th dimension of the *i*-th individual, and $r\in \left(0,1\right)$ is the control parameter to balance chaotic divergence and structural stability.

After $M_{c}$ chaotic iterations, the Kent-mapped value is scaled to the search space:

(13) $${OBL}_{i,j}=K_{i,j}^{\left(M_{c}\right)}\cdot \left(U_{j}-L_{j}\right)+L_{j}$$

The control parameter is set to r=0.4, a value widely adopted in the chaotic optimization literature for producing sequences with favorable ergodicity and uniform distribution properties [23,24]. The chaotic iteration count is set to $M_{c}=10$. A sensitivity analysis varying r∈{0.3,0.4,0.5} and $M_{c}\in \{5,10,15\}$ on the CEC 2017 suite at D = 30 did not reveal statistically significant differences among the tested configurations (Friedman test, *p* = 0.12 > 0.05), indicating robustness to moderate perturbations of r and $M_{c}$.

The motivation for OBL is that, when the optimum’s location is unknown, the opposite candidate is as likely as the original point to lie closer to it [39,40]. For each Kent-mapped individual ${OBL}_{i}$, its opposite is computed as:

(14) $${OBL}_{i,j}^{opp}=L_{j}+U_{j}-{OBL}_{i,j}$$

By greedily selecting the better of the two candidates according to Equation (15), KCOL improves the expected quality of the initial population.

(15) $$x_{i}^{0}=argmin\left\{f\left({OBL}_{i}\right),f\left({OBL}_{i}^{opp}\right)\right\}$$

The greedy selection then guarantees that the retained individual is never worse than the original one.

### 3.3. APO-Inspired Dual-Modal Evasion (APO-DME)

The first defense mechanism in CPO uses the current best solution $x_{CP}$ to guide exploration. This mechanism depends entirely on $x_{CP}$ to determine the search direction. If $x_{CP}$ is trapped in a local optimum, the entire population is attracted toward that suboptimal region, causing stagnation. Individuals that would otherwise explore other regions are pulled back by the best-solution guidance. Real-world optimization problems present heterogeneous local structures: some regions require fine-grained local search to exploit promising gradients, while others require long-distance jumps to escape deep basins. This observation motivates a bimodal exploration mechanism that stochastically alternates between local refinement and global escape with equal probability.

The Arctic Puffin Optimization (APO) algorithm [25] uses two predator-evasion behaviors. One is rapid directional swimming through coordinated movements, which provides local fine-grained exploration:

(16) $$step=x_{{rand}_{1}}-x_{{rand}_{2}},\;x_{i}^{new}=x_{i}+\varepsilon \cdot step$$

where $\varepsilon ∼U\left(0,1\right)$ produces step sizes distributed over the interval $\left[0,∥step∥\right]$. This mode leverages the structural information of the population encoded in the difference vector, enabling local exploration with step sizes on the same order as the typical inter-individual distance.

The other involves sudden long-distance directional changes modeled by a Levy flight:

(17) $$step=x_{{rand}_{1}}-x_{{rand}_{2}},\;x_{i}^{new}=x_{i}+F_{L}\cdot L_{Levy}\left(D\right)\cdot step$$

where $L_{Levy}\left(D\right)$ is a D-dimensional Levy flight vector:

(18) $$L_{Levy}\left(D\right)=\frac{u}{{\left|v\right|}^{1/\beta }}$$

where $u∼N\left(0,\sigma_{u}^{2}\right)$ with:

(19) $$\sigma_{u}={\left(\frac{\Gamma \left(1+\beta \right)sin\left(\pi \beta /2\right)}{\Gamma \left(\left(1+\beta \right)/2\right)\cdot \beta \cdot 2^{\left(\beta -1\right)/2}}\right)}^{1/\beta }$$

and $v∼N\left(0,1\right)$; the setting β=1.5 provides a balance between frequent small steps (local search) and occasional large jumps (global exploration). Levy flights produce step lengths following a heavy-tailed distribution $P\left(\left|s\right|\right)∼{\left|s\right|}^{-1-\beta }$, enabling occasional long-distance jumps that can escape the deep basins of local optima.

The APO-DME position update for the first defense of MSCPO is:

(20) $$x_{i}^{t+1}=\left\{\begin{array}{ll} x_{i}^{t}+\varepsilon_{c}\cdot \left(x_{r_{1}}^{t}-x_{r_{2}}^{t}\right), & \mathrm{if}\;rand<0.5\\ x_{i}^{t}+F_{L}\cdot L_{Levy}\left(D\right)\cdot \left(x_{r_{1}}^{t}-x_{r_{2}}^{t}\right), & \mathrm{otherwise} \end{array}\right.$$

where $\varepsilon_{c}∼U\left(0,1\right)$ is the uniform random perturbation coefficient. $F_{L}=0.5$ is the Levy flight scaling factor, and rand is a random value in [0, 1].

### 3.4. Adaptive Dual-Differential Perturbation (ADDP)

The second defense mechanism in CPO employs a single differential vector $\left(x_{r1}-x_{r2}\right)$, which provides only one search direction per update. In complex multimodal landscapes, effective exploration often requires simultaneous movement along multiple promising directions. With only one differential, the resulting search direction depends entirely on random selection. An unfavorable pair $\left(r1,\;r2\right)$ can produce a poor direction with no alternative. This is problematic in landscapes with narrow valleys, where the optimal direction may not align with any single random differential vector.

Moreover, the original second defense never exploits the current best solution: its search direction is built solely from random population members. Combining a population-defined direction with a direction toward the best-so-far solution provides complementary guidance within a single update.

The ADDP strategy addresses these limitations through a redesigned mechanism. The intermediate reference point is retained and computed via binary mask blending, as shown in Equation (21):

(21) $$x_{i}^{mid}=U_{1}\cdot x_{i}^{t}+\left(1-U_{1}\right)\cdot \left(y_{i}^{t}+U_{2}\cdot \left(x_{r1}^{t}-x_{r2}^{t}\right)\right)$$

where $U_{1}$ and $U_{2}$ are independent binary random vectors. The binary mask enables component-wise selection: some dimensions retain their current values, while others explore new regions. The ADDP-enhanced second defense is:

(22) $$x_{i}^{t+1}=\left\{\begin{array}{ll} x_{i}^{mid}+F\cdot \left(x_{r1}^{t}-x_{r2}^{t}\right)+F\cdot \left(x_{CP}^{t}-x_{i}^{t}\right), & \mathrm{if}\;rand>0.5\\ x_{i}^{mid}, & \mathrm{otherwise} \end{array}\right.$$

where rand is a random value in [0, 1], and F is a random coefficient drawn uniformly from [0, 1], applied identically to both differential vectors. The ADDP-enhanced position update uses a population differential and an elite differential. The population differential $\left(x_{r1}^{t}-x_{r2}^{t}\right)$ promotes diversity by exploring along population-defined directions, encoding structural information about the relative geometry of population members. The elite differential $\left(x_{CP}^{t}-x_{i}^{t}\right)$ points toward the current best-known solution. The perturbation is adaptive in the sense that both differential vectors are constructed from the current population: as the population converges, the magnitudes of the two differentials decrease automatically, so the perturbation scale reduces over the course of the search without any explicit parameter schedule.

### 3.5. Periodic Dynamic Adaptive Perturbation (PDAP)

The fourth defense mechanism in CPO uses a fixed parameter α. The balance between stochastic exploration and deterministic exploitation fundamentally changes during optimization. Early iterations need strong stochasticity to explore widely, while late iterations require deterministic refinement to converge precisely. A fixed convergence-speed factor α imposes the same balance throughout the optimization process. Moreover, many optimization landscapes contain periodic local structures in which static perturbation strategies become trapped. The original mechanism lacks a periodic oscillation component and is therefore vulnerable to such structures.

MSCPO replaces the fourth defense mechanism with PDAP, which integrates periodic trigonometric perturbation, a random-peer reference, and a fitness-conditioned update:

(23) $$x_{i}^{t+1}=\left\{\begin{array}{ll} x_{i}^{t}+\eta_{t}\cdot \left(P_{i}-I\cdot x_{i}^{t}\right), & \mathrm{if}\;f\left(x_{i}^{t}\right)>f\left(P_{i}\right)\\ x_{i}^{t}+\eta_{t}\cdot \left(x_{i}^{t}-P_{i}\right), & \mathrm{otherwise} \end{array}\right.$$

where $\eta_{t}=sin\left(2\pi U_{1}\right)+cos\left(2\pi U_{2}\right)$ is the periodic trigonometric perturbation, and $U_{1},U_{2}∼U\left(0,1\right)$ are independent. $P_{i}=x_{k}^{t}$ is the random-peer reference point, with k chosen uniformly at random from the current individuals excluding i. The random integer coefficient I is defined as follows:

(24) $$I=round\left(1+rand\right)\in \{1,\;2\}$$

where I=1 and I=2 each occur with probability 0.5. This coefficient scales the attraction term when the current individual is worse than the reference peer, adding variability to the update magnitude.

As shown in Equation (23), the PDAP position update depends on the relative fitness of $x_{i}$ and $P_{i}$. If the individual $x_{i}$ is worse than the random peer $P_{i}$, the update term $\left(P_{i}-I\cdot x_{i}^{t}\right)$ pulls $x_{i}$ toward $P_{i}$ with a magnitude scaled by I∈{1,2}, thereby directing the search toward a potentially better region. Otherwise, the update term $\left(x_{i}^{t}-P_{i}\right)$ pushes $x_{i}$ away from $P_{i}$, preserving the individual’s advantageous position while still introducing diversity through the periodic perturbation $\eta_{t}$. The update is adaptive in the sense that both its direction and its reference point depend on the current fitness state of the population: the relative fitness comparison determines whether the individual is attracted toward or repelled from a randomly resampled peer.

### 3.6. Flowchart of MSCPO

**Figure 1:**
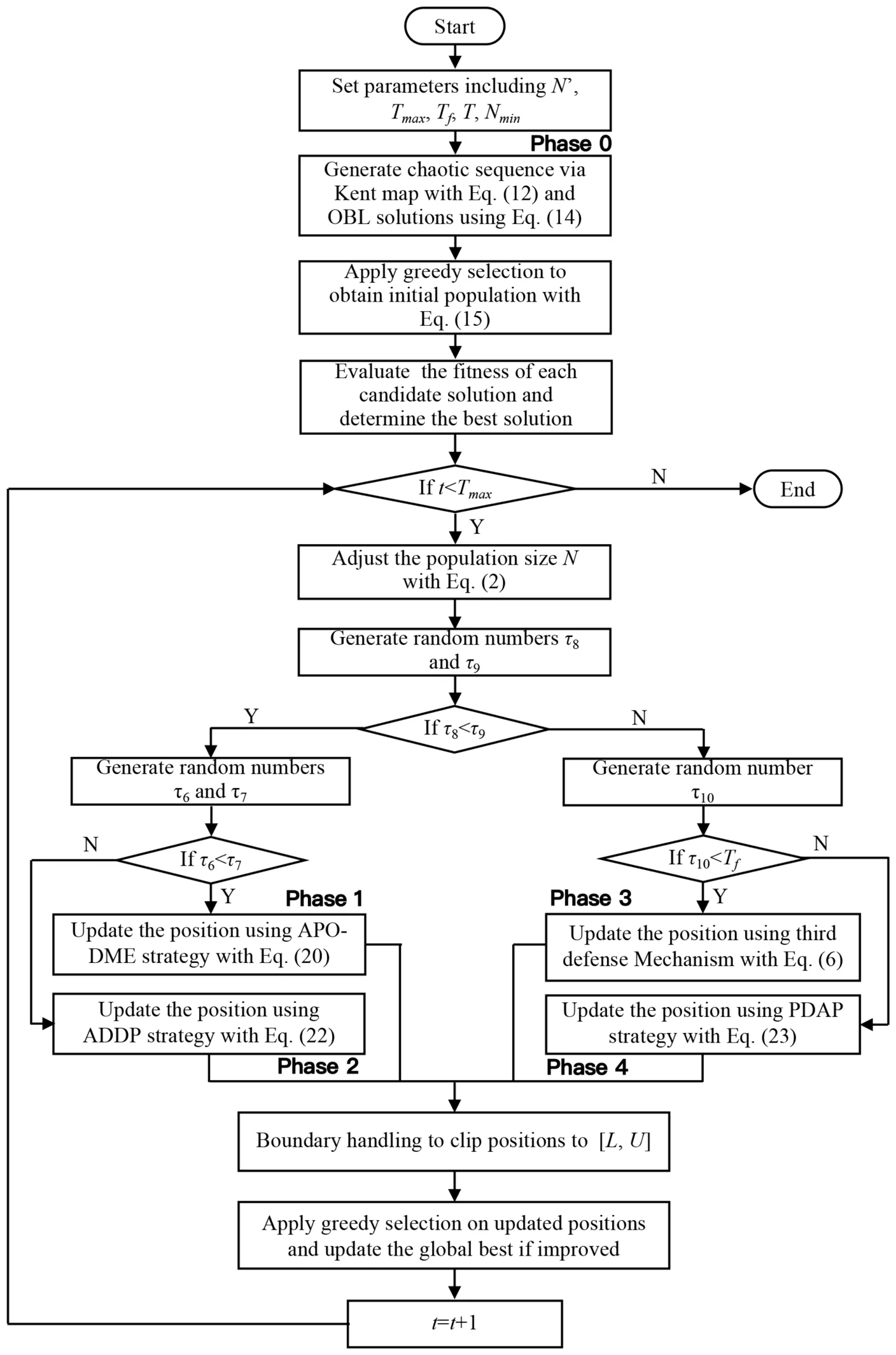
Flowchart of the MSCPO algorithm. The five numbered phases correspond to Table 1 in Section 3.1: Phase 0 (initialization)—KCOL; Phase 1—APO-DME replaces the first defense; Phase 2—ADDP replaces the second defense; Phase 3—original odor defense preserved; Phase 4—PDAP replaces the fourth defense.

The overall flowchart of MSCPO is illustrated in Figure 1.

### 3.7. Theoretical Analysis

The convergence of MSCPO can be analyzed within the standard probabilistic search framework [41,42]. Let $X_{t}$ denote the population position matrix at iteration t, and let $f\left(X_{t}\right)$ denote the corresponding fitness values. The position update in MSCPO is governed by four stochastic mechanisms: KCOL, APO-DME, ADDP, and PDAP, among which APO-DME, ADDP, and PDAP are activated probabilistically through the CPO defense selection framework, whereas KCOL acts at initialization.

First, MSCPO satisfies the improvement condition: the greedy selection applied after every position update retains a newly generated position only when it does not worsen the current fitness, so that $f\left(x_{best}^{t+1}\right)\leq f\left(x_{best}^{t}\right)$ for all iterations t, where $x_{best}^{t}$ denotes the best solution found up to iteration t. The best-fitness sequence therefore forms a non-increasing stochastic process.

Second, MSCPO satisfies the reachability condition: every subset of the bounded search space $S=\left[L,U\right]$ with positive volume is sampled with strictly positive probability. KCOL generates samples over S at initialization; the uniform-perturbation mode of APO-DME produces new positions with positive density over S; and the Levy-flight mode follows a heavy-tailed distribution with unbounded support, so that any neighborhood of any point in S is reached with positive probability at every iteration. Hence, for the optimality region $R_{\varepsilon }=\left\{x\in S:f\left(x\right)-f^{*}\leq \varepsilon \right\}$ with any ε>0, every iteration has a positive probability of entering $R_{\varepsilon }$, independently of the search history. All update operators produce bounded displacements on S: KCOL confines samples within $\left[L,U\right]$; the APO-DME step is scaled by the step factor and the Levy scaling parameter $F_{L}$; the ADDP perturbation is scaled by $F\in \left[0,\;1\right]$; and the PDAP is bounded by $\eta_{t}\in [-2,2]$. The search therefore remains stable on bounded domains.

Under the improvement and reachability conditions, the convergence theorem for random search algorithms [41] applies: the best-so-far solution of MSCPO converges in probability to the global optimum:

(25) $$\underset{t\to \infty }{\lim}P\left(f\left(x_{best}^{t}\right)-f^{*}\leq \varepsilon \right)=1,\;\;\forall \varepsilon >0$$

This result concerns the limit behavior of the algorithm: it guarantees that the global optimum is attained with probability one as the number of iterations grows, but it does not specify a convergence rate, which depends on the problem structure.

### 3.8. Computational Complexity Analysis

#### 3.8.1. Time Complexity

The time complexity of MSCPO is determined by two components: the initialization phase and the main loop. Let N denote the initial population size, D the problem dimension, $T_{max}$ the maximum number of iterations, and $O\left(f_{c}\right)$ the complexity of a single objective-function evaluation.

The initialization phase proceeds as follows. First, random population initialization takes $O\left(ND\right)$. Second, evaluating the initial fitness of all individuals takes $O\left(Nf_{c}\right)$. Third, the Kent chaotic map iterates $M_{c}$ times over the N×D matrix, taking $O\left(M_{c}ND\right)$, where $M_{c}=10$ is a fixed constant. Opposition-based learning then generates N opposite solutions and performs greedy selection, requiring $O\left(ND\right)$ for solution generation and $O\left(Nf_{c}\right)$ for the additional fitness evaluations. KCOL therefore consumes exactly 2N objective evaluations during initialization, compared with N in the original CPO. Therefore, the total initialization complexity is $O\left(M_{c}ND+ND+Nf_{c}\right)=O(N(M_{c}D+f_{c}))$.

The main loop runs for $T_{max}$ iterations. Within each iteration, the algorithm processes $N\left(t\right)$ individuals, where $N\left(t\right)$ decreases from N to $N_{min}$ via cyclic population reduction, and the average population size over a run is $\bar{N}=\left(N+N_{min}\right)/2\approx 0.9N$. For each individual, the complexity breakdown is as follows:

- In APO-DME, selecting two random individuals and computing the step vector takes $O\left(D\right)$ Levy flight generation takes $O\left(D\right)$, and the position update takes $O\left(D\right)$;
- In the second defense mechanism (ADDP), computing the reference point and the dual-differential perturbation takes $O\left(D\right)$;
- For the odor defense, computing the sensory factor $S_{t}$ requires $O\left(N\left(t\right)\right)$ for the summation, and the position update takes $O\left(D\right)$. The summation is recomputed for each individual entering this branch, because the fitness vector changes as individuals are updated within the same iteration;
- In PDAP, selecting a random peer via a random permutation takes $O\left(N\left(t\right)\right)$, computing the periodic perturbation takes $O\left(D\right)$, and updating the position takes $O\left(D\right)$. In addition, the boundary handling and fitness evaluation take $O\left(D\right)$ and $O\left(f_{c}\right)$ per individual, respectively.

Each individual enters the exploration branch (APO-DME or ADDP, costing $O(D+f_{c})$) or the exploitation branch (the odor defense or PDAP, costing $O(N\left(t\right)+D+f_{c})$) with probability 0.5. The expected per-iteration complexity is therefore $O\left(N\left(t\right)\left(D+f_{c}\right)+{N(t)}^{2}\right)$, and the worst-case per-iteration complexity is $O\left(N(t)(D+f_{c}+N(t))\right)$. With an average population size of $\bar{N}$ over $T_{max}$ iterations, the total complexity of the main loop is $O\left(T_{max}\left(\bar{N}D+\bar{N}f_{c}+{\bar{N}}^{2}\right)\right)$.

Combining the initialization and the main loop, we obtain the overall time complexity of MSCPO:

(26) $$O\left(M_{c}ND+Nf_{c}+T_{max}\bar{N}\left(D+f_{c}+\bar{N}\right)\right)$$

The same $O\left({N(t)}^{2}\right)$ summation cost arises in the third and fourth defense mechanisms of the original CPO, whose overall complexity is $O\left(ND+Nf_{c}+T_{max}\bar{N}\left(D+f_{c}+\bar{N}\right)\right)$. The two algorithms thus share the same asymptotic form. For standard benchmark functions where $O\left(f_{c}\right)=O\left(D\right)$, and for engineering problems whose closed-form objectives with multiple constraint terms also cost $O\left(D\right)$ per evaluation, the total time complexity simplifies to $O\left(T_{max}ND\right)$ when $D\geq \bar{N}$; for low-dimensional problems with $D<\bar{N}$, the complexity is $O\left(T_{max}{\bar{N}}^{2}\right)$, which remains identical in asymptotic order to the original CPO.

#### 3.8.2. Spatial Complexity

The spatial complexity is determined by the memory required to store the population and auxiliary variables:

- Population matrix: $O\left(ND\right)$;
- Individual historical best matrix: $O\left(ND\right)$ which is maintained in both CPO and MSCPO;
- Fitness vector: $O\left(N\right)$;
- Global best solution: $O\left(D\right)$;
- Kent chaotic matrix (initialization only): $O\left(ND\right)$;
- Intermediate variables: $O\left(D\right)$.

The overall spatial complexity is:

(27) $$O\left(ND+ND+N+D+ND+D\right)=O\left(ND\right)$$

Overall, MSCPO has a time complexity of $O\left(T_{max}ND\right)$ and a spatial complexity of $O\left(ND\right)$, both identical in asymptotic order to those of the original CPO. The four enhancement strategies add O(D) arithmetic operations per individual and exactly N additional objective evaluations, which are incurred once at initialization and account for about 0.1% of the total evaluation budget under the experiment settings (e.g., 30 out of approximately 27,060 evaluations for N=30, $T_{max}=1000$) and about 0.2% on the engineering problems (N=100, $T_{max}=500$).

## 4. Experimental Verification and Analysis

This section evaluates the Multi-Strategy Enhanced Crested Porcupine Optimizer (MSCPO). The primary objective is to assess the algorithm’s performance against a diverse set of established optimization methods, including classical evolutionary algorithms (GA [26], DE [11]), swarm intelligence algorithms (WOA [10], HHO [27], DBO [28], GJA [29], SSA [30], BBO [31]), the original CPO [8], and two high-performance CEC competition algorithms (L-SHADE [32], LSHADE-SPACMA [33]). The evaluation was conducted on the CEC 2017 and CEC 2022 benchmark suites across various dimensions, with a fixed population size of 30 and a maximum of 1000 iterations per run. Under this fixed-iteration protocol, the total number of objective evaluations per run may differ across algorithm families, because some competitors employ population size reduction (e.g., L-SHADE, LSHADE-SPACMA, and the CPO family) while others evaluate the full population in every iteration. All algorithms were executed over 30 independent runs to ensure statistical reliability. Statistical significance was assessed using the Wilcoxon signed-rank test and Friedman mean rank test at a significance level of α=0.05.

Section 4.1 describes the parameters and experimental environment. Section 4.2 presents the ablation study of the four enhancement strategies. Section 4.3 presents a comparative analysis on CEC 2017, including the Wilcoxon signed-rank test and the Friedman mean rank test across the tested dimensions. Section 4.4 presents a comparative analysis of CEC 2022. Detailed numerical results (mean and standard deviation for each function) are provided in Appendix A, and only the statistically aggregated results are presented here.

### 4.1. Experimental Settings and Comparative Algorithms

Experiments were conducted on two widely recognized benchmark suites, CEC 2017 [43] and CEC 2022 [44]. Table 2 summarizes the CEC 2017 test suite, categorizing functions into unimodal, multimodal, hybrid, and composition types. Each function is listed with its number, name, lower and upper bounds, and minimum value ($f_{min}$). Table 3 outlines the CEC 2022 test suite, detailing types (unimodal, basic, hybrid, and composition), numbers, names, bounds, and minimum values ($f_{min}$).

To comprehensively evaluate the proposed MSCPO from multiple perspectives, a diverse set of algorithms spanning different eras and paradigms was selected for comparison:

- Classical evolutionary algorithms: Genetic Algorithm (GA) [26] and Differential Evolution (DE) [11].
- Swarm intelligence algorithms: Whale Optimization Algorithm (WOA) [10] and original Crested Porcupine Optimizer (CPO) [8].
- State-of-the-art high-performance algorithms: Harris Hawks Optimization (HHO) [27], Dung Beetle Optimizer (DBO) [28], Gekko Japonicus Algorithm (GJA) [29], Sparrow Search Algorithm (SSA) [30], and Beaver Behavior Optimizer (BBO) [31].
- High-performance CEC competition algorithms: L-SHADE [32] and its variant LSHADE-SPACMA [33]. The former is the winner of the IEEE CEC 2014 international optimization competition, and the latter ranked among the top four of the CEC 2017 single-objective competition.

The complete parameter settings of all compared algorithms, including the proposed MSCPO, are listed in Table A1 (Appendix A). All experiments were conducted on the same computing platform, comprising a 2.60 GHz Intel i7-10750H CPU, 16 GB of RAM, and the Windows 10 operating system, and all algorithms were implemented in MATLAB R2023b.

### 4.2. Ablation Study Results and Analysis

To validate the effectiveness of each enhancement strategy and their interactions, we conducted a full-factorial ablation study covering all $2^{4}=16$ combinations of the four strategies (KCOL, APO-DME, ADDP, and PDAP) on the 29 benchmark functions from the CEC 2017 test suite (D = 30) under 30 independent runs. Variants are named by listing the included strategies after “CPO_” (e.g., CPO_KCOL_APODME includes KCOL and APO-DME only); three-strategy variants are equivalently denoted MSCPO_woX, indicating MSCPO without strategy X. Table 4 lists the composition of all 16 variants. As the raw fitness values of different functions differ by orders of magnitude, the comparison relies on scale-invariant quantities only: the Friedman mean rank, the number of best/worst results, and the Wilcoxon signed-rank test against MSCPO.

**Figure 2:**
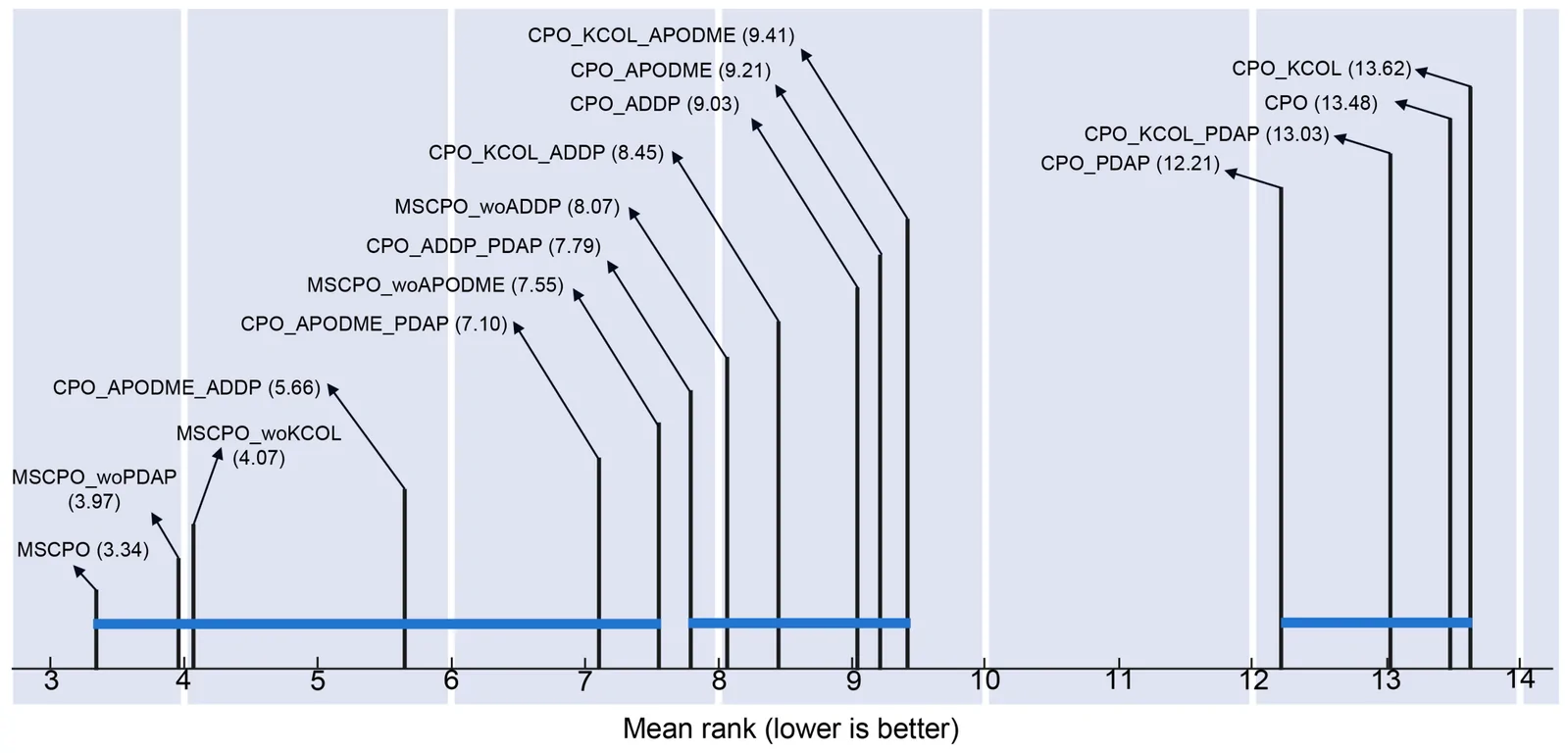
Critical difference diagram for the full factorial ablation (k=16 variants, N=29 CEC 2017 functions at D = 30). Variants are ordered by Friedman mean rank; low rank (left) indicates better performance. Blue horizontal bars connect variants that do not differ significantly at α = 0.05 according to the Nemenyi post hoc test ($q_{0.05}=3.426$, CD = 4.28).

All 16 variants were compared under identical settings. The single-strategy tier is summarized in Table 5, and the complete factorial comparison is reported in Table 6. The complete per-function results of all 16 variants are available from the corresponding author upon reasonable request. In Table 5 and Table 6, Mean Rank is the average Friedman rank across the 29 test functions for each variant, where a lower value indicates better overall performance; No. Best and No. Worst give the number of functions on which a variant achieves the best and worst mean fitness values, respectively. The Friedman test statistic is reported to assess whether significant performance differences exist among the variants (*p* < 0.05 indicates statistical significance). To further assess pairwise comparisons, the Nemenyi post hoc test with a Critical Difference (CD) threshold [34] is applied; the CD is computed using Equation (28):

(28) $$CD=q_{\alpha }\sqrt{k\left(k+1\right)/6N}$$

where $q_{\alpha }$ is the critical value from the Studentized range distribution, k is the number of algorithms, and N is the number of test functions. In this study, N=29, with k=6 ($q_{0.05}=2.850$, CD = 1.40) for the single-strategy tier in Table 5 and k=16 ($q_{0.05}=3.426$, CD = 4.28) for the full comparison in Table 6. The corresponding CD diagram is shown in Figure 2.

Table 5 summarizes the single-strategy tier. Consistent with the design intent, ADDP (mean rank 2.8621) and APO-DME (mean rank 2.9655) provide the largest individual contributions, and both differ significantly from the original CPO (4.8276), whereas CPO_PDAP (4.1034) and CPO_KCOL (4.8621) alone do not. MSCPO achieves the best mean rank (1.3793), obtains the best result on 22 of the 29 functions, and is never the worst on any function; its rank gap to every other variant in this tier exceeds the CD of 1.40, i.e., MSCPO differs significantly from all five variants.

Table 6 reports the complete factorial comparison. Three observations emerge. First, the complete MSCPO attains the best mean rank (3.3448), achieves the best result on 8 of the 29 functions, and is never the worst on any function. Second, the leave-one-out tier shows that removing any single strategy degrades the mean rank (3.9655, 4.0690, 7.5517, and 8.0690 for MSCPO_woPDAP, MSCPO_woKCOL, MSCPO_woAPODME, and MSCPO_woADDP, respectively); the degradation is statistically significant when APO-DME or ADDP is removed (*p* = 3.00 × 10^−5^ and *p* = 1.27 × 10^−3^, Wilcoxon signed-rank test), but not when KCOL or PDAP is removed (*p* = 1.41 × 10^−1^ and *p* = 6.817 × 10^−1^). Third, pairwise combinations of the exploration-phase strategies markedly outperform their constituent single strategies; CPO_APODME_ADDP attains the best pairwise rank (5.6552), and MSCPO significantly outperforms all single-strategy and pairwise variants (*p* < 0.01). It is worth noting that KCOL alone does not significantly improve over CPO on this suite (mean ranks 13.6207 versus 13.4828, within the same CD group), which is expected for an initialization strategy whose direct effect is diluted over 1000 iterations; its contribution emerges in combination, removing KCOL from the complete MSCPO degrades the mean rank from 3.3448 to 4.0690, and its greedy selection never degrades the initial population quality.

The factorial structure of Table 6 further indicates that the four strategies combine without destructive interactions: adding APO-DME, ADDP, or PDAP to any combination improves its mean rank (in all 24 nested comparisons involving these three strategies), whereas the effect of adding KCOL is neutral on average, consistent with its role as an initialization operator whose benefit materializes in the complete configuration, as evidenced by the leave-one-out analysis above. We therefore describe the four strategies as providing complementary, phase-targeted contributions, whose complete combination yields the best overall performance.

In summary, the full-factorial ablation establishes that the four strategies provide complementary, phase-targeted contributions, and that the complete four-strategy configuration is required to achieve the best overall performance.

### 4.3. Numerical Experiments on CEC 2017

In this section, MSCPO was compared against 11 optimization algorithms on the CEC 2017 test suite across four dimensions (D = 10, 30, 50, and 100). Detailed numerical results (mean and standard deviation for each function) are provided in Table A2, Table A3, Table A4 and Table A5 of Appendix A.

#### 4.3.1. CEC 2017 Wilcoxon Signed-Rank Test

The Wilcoxon signed-rank test was used to compare MSCPO against each competitor on a per-function basis. Detailed results are provided in Table A6, Table A7, Table A8 and Table A9 of Appendix A. Table 7 summarizes the results, comparing MSCPO against CPO, GA, DE, WOA, HHO, DBO, GJA, SSA, BBO, L-SHADE, and LSHADE-SPACMA. The results are marked with ‘+’, ‘−’, and ‘=’, indicating whether MSCPO performed better than, worse than, or comparably to the corresponding competitors, respectively.

The detailed analysis is as follows:

(1) At D = 10, MSCPO outperformed (and was inferior to) CPO, GA, DE, WOA, HHO, DBO, GJA, SSA, BBO, L-SHADE, and LSHADE-SPACMA on 20 (1), 29 (0), 23 (4), 29 (0), 29 (0), 29 (0), 27 (1), 28 (0), 28 (0), 19 (3), and 14 (5) functions, respectively, with 8, 0, 2, 0, 0, 0, 1, 1, 1, 7, and 10 draws. Overall, MSCPO won 275 and lost 14 of these pairwise comparisons against all 11 competitors. It dominated GA, WOA, HHO, and DBO (29 wins, 0 losses each), as well as SSA and BBO (28 wins, 0 losses each). The strongest challenges came from LSHADE-SPACMA (14 wins, 5 losses, 10 draws) and L-SHADE (19 wins, 3 losses, 7 draws), whose success-history-based parameter adaptation provides advantages in smooth search landscapes. DE presented a moderate challenge (23 wins, 4 losses), while CPO was clearly surpassed (20 wins, 1 loss). This dominance at D = 10 confirms that the four phase-distributed strategies provide decisive advantages in low-dimensional spaces.

(2) At D = 30, MSCPO outperformed (and was inferior to) CPO, GA, DE, WOA, HHO, DBO, GJA, SSA, BBO, L-SHADE, and LSHADE-SPACMA on 20 (0), 29 (0), 26 (0), 27 (0), 27 (2), 29 (0), 17 (7), 26 (2), 19 (5), 23 (4), and 12 (9) functions, respectively, with 9, 0, 3, 2, 0, 0, 5, 1, 5, 2, and 8 draws. Overall, MSCPO won 255 and lost 29 of these comparisons. Its advantage over CPO strengthened (20 wins, 0 losses vs. 20 wins, 1 loss at D = 10), and it dominated DE (26 wins, 0 losses vs. 23 wins, 4 losses at D = 10). This suggests that APO-DME’s dual-modal exploration and ADDP’s adaptive differential perturbation become increasingly effective as the multimodal complexity increases. However, competitive pressure escalated: LSHADE-SPACMA surpassed MSCPO on 9 functions (up from 5 at D = 10), and GJA became more competitive, with 7 wins (up from 1). BBO also gained ground (5 wins, up from 0). Total losses increased from 14 to 29, reflecting the growing challenge of the expanded search space.

(3) At D = 50, MSCPO outperformed (and was inferior to) CPO, GA, DE, WOA, HHO, DBO, GJA, SSA, BBO, L-SHADE, and LSHADE-SPACMA on 23 (0), 29 (0), 29 (0), 27 (0), 27 (2), 29 (0), 16 (10), 23 (2), 12 (9), 19 (4), and 9 (8) functions, respectively, with 6, 0, 0, 2, 0, 0, 3, 4, 8, 6, and 12 draws. Overall, MSCPO won 243 and lost 35 of these comparisons. It dominated CPO (23 wins, 0 losses), and GA, DE, and DBO (29 wins, 0 losses each). GJA surpassed MSCPO on 10 functions (up from 7 at D = 30), BBO achieved 9 wins (up from 5 at D = 30), and LSHADE-SPACMA reached 8 wins (with more draws). Total losses increased to 35 from 29 at D = 30. The adaptive mechanisms in ADDP and PDAP remained effective at this dimensionality, as evidenced by MSCPO’s sustained dominance over CPO and the suppression of DE.

(4) At D = 100, MSCPO outperformed (and was inferior to) CPO, GA, DE, WOA, HHO, DBO, GJA, SSA, BBO, L-SHADE, and LSHADE-SPACMA on 24 (0), 29 (0), 29 (0), 27 (2), 26 (3), 28 (0), 10 (13), 18 (7), 9 (18), 12 (9), and 9 (13) functions, respectively, with 5, 0, 0, 0, 0, 1, 6, 4, 2, 8, and 7 draws. Overall, MSCPO won 221 and lost 65 of these comparisons. It still dominated CPO, GA, DE, WOA, HHO, and DBO (all with 24+ wins and 0–3 losses), but faced significant challenges from BBO (9 wins, 18 losses), GJA (10 wins, 13 losses), and LSHADE-SPACMA (9 wins, 13 losses). This is the first dimension at which LSHADE-SPACMA achieved more wins than losses against MSCPO. The total losses of 65 nearly double relative to D = 50 (35) and increased more than four-fold relative to D = 10 (14). This escalation reflects two factors: first, as the search space volume expands, the depth of exploration achievable by any algorithm is limited; second, the population-diversity-preserving strategies in BBO and GJA, and the success-history-based adaptation in LSHADE-SPACMA, provide advantages under extremely budget-constrained high-dimensional optimization that the MSCPO design does not fully match.

**Figure 3:**
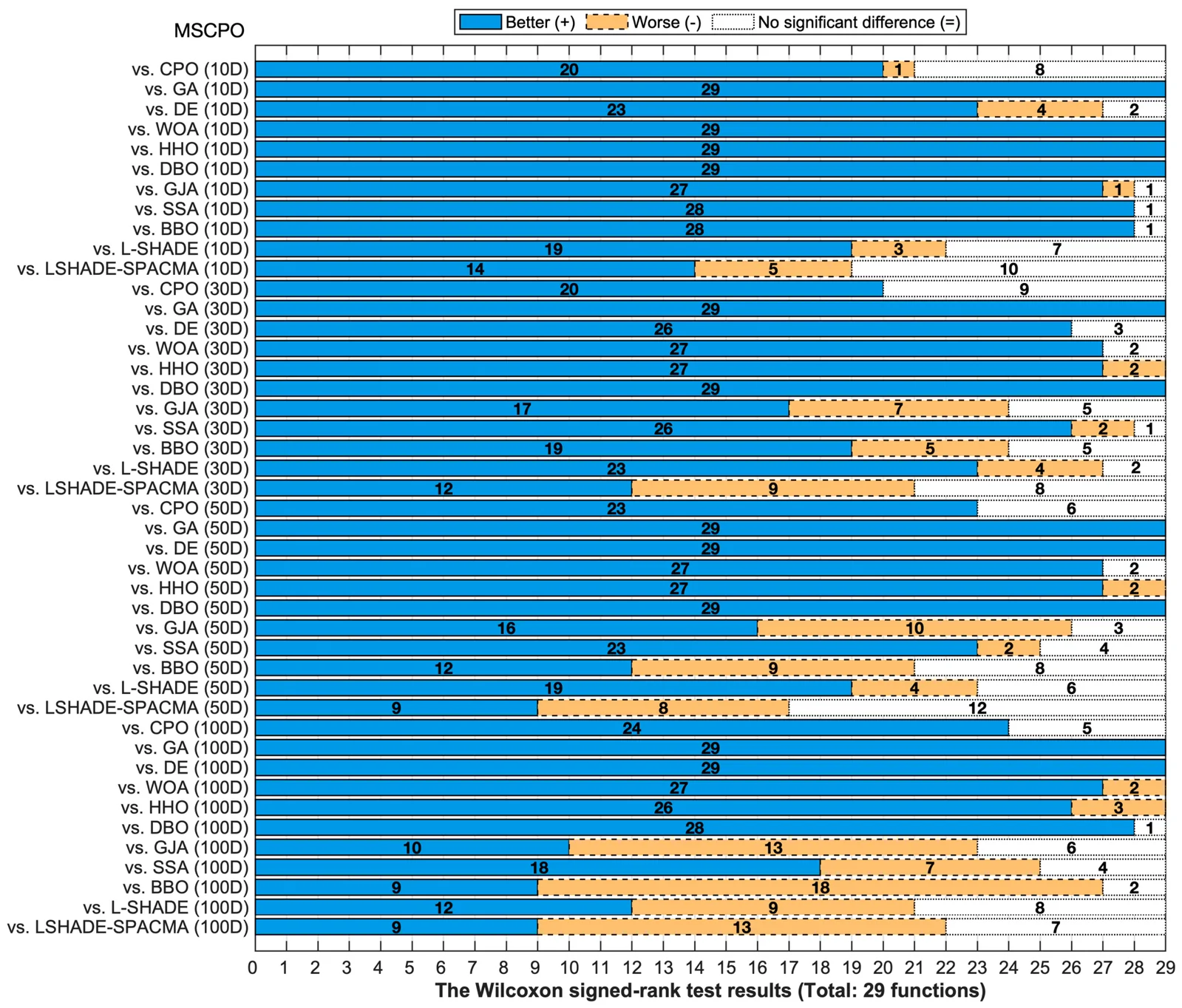
Wilcoxon signed-rank test outcomes (α = 0.05) of MSCPO against each competitor on the 29 CEC 2017 functions (D = 10, 30, 50, 100; 30 runs per function). “+”/“−”: number of functions on which MSCPO is significantly better/worse; “=”: p≥0.05.

As shown by the “Total 1” columns of Table 7, MSCPO won more comparisons than it lost against all 11 competitors across the four dimensions. It dominated GA (116 wins, 0 losses), DBO (115 wins, 0 losses), WOA (110 wins, 2 losses), HHO (109 wins, 7 losses), and DE (107 wins, 4 losses). It also led CPO (87 wins, 1 loss) and SSA (95 wins, 11 losses). Against L-SHADE (73 wins, 20 losses), the advantage narrowed at higher dimensions. The most competitive rivals were GJA (70 wins, 31 losses) and BBO (68 wins, 32 losses), against which MSCPO’s advantage was marginal and reversed at D = 100. Against LSHADE-SPACMA (44 wins, 35 losses), the relationship was near-balanced, as LSHADE-SPACMA’s CMA-ES covariance matrix learning provides complementary strengths to MSCPO’s phase-distributed design. The “Total 2” row of Table 7 reveals a clear and consistent dimension-dependent performance trend: MSCPO’s win count decreases monotonically from 275 at D = 10 to 255 at D = 30, 243 at D = 50, and 221 at D = 100, while the losses increase from 14 to 29, 35, and 65. This trend is visually confirmed by Figure 3. Overall, MSCPO won 994, lost 143, and drew 139 of the 1276 pairwise comparisons, demonstrating robustness across the full dimensionality range on CEC 2017, although its advantage diminished and partially reversed against the strongest competitors at D = 100.

#### 4.3.2. CEC 2017 Friedman Mean Rank Test

**Figure 4:**
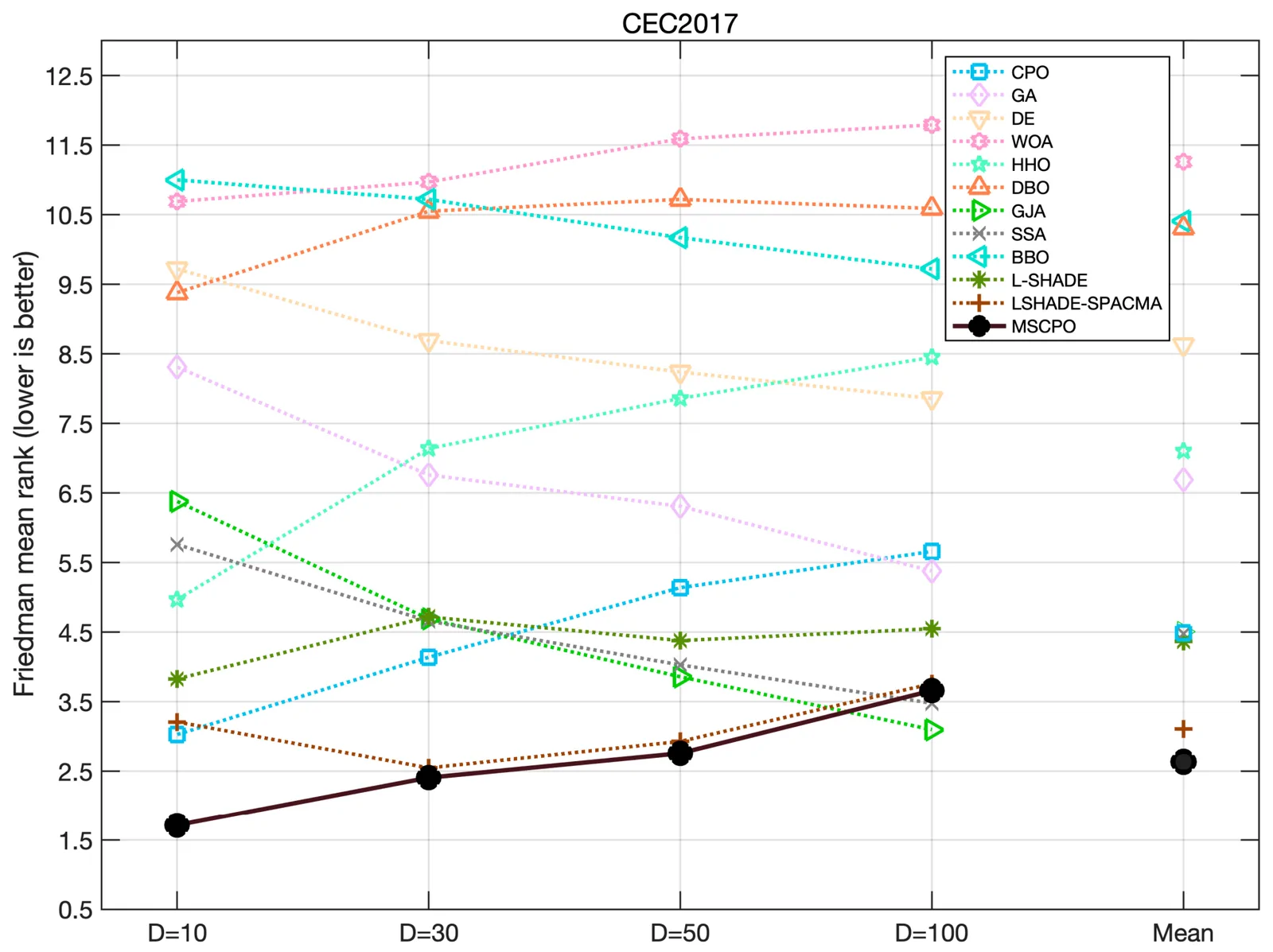
Friedman mean ranks (lower is better) on the 29 CEC 2017 functions (D = 10, 30, 50, 100; 30 runs per function). Ranks are computed per function and averaged over all functions; “Mean” denotes the average over the four dimensions; each dimension is an independent experiment.

In this section, we used the Friedman mean rank test, a robust non-parametric method, to statistically analyze the performance of the competing algorithms on the CEC 2017 test suite. Table 8 presents the results of the compared algorithms over 30 runs. In particular, the last two columns of Table 8 show that MSCPO achieved the best overall mean rank averaged over the four dimensions, ranking first at D = 10, 30, and 50. LSHADE-SPACMA and L-SHADE followed closely behind. Additionally, Figure 4 visualizes these rankings, illustrating the overall superiority of MSCPO over its competitors. The detailed analysis is as follows:

At D = 10, MSCPO ranked first (mean rank 1.72), outperforming all 11 competitors. The gap to the second-ranked CPO (mean rank 3.03) was 1.31. At D = 30, MSCPO again ranked first (mean rank 2.41), with LSHADE-SPACMA following closely (mean rank 2.55, gap = 0.14), which made this the most competitive dimension for the top two. At D = 50, MSCPO ranked first (mean rank 2.76), followed by LSHADE-SPACMA (mean rank 2.93) and GJA (mean rank 3.86). At D = 100, MSCPO ranked third (mean rank 3.66), behind GJA (3.10) and SSA (3.48) but ahead of the other 9 competitors. This is the first dimension at which MSCPO did not rank first. The Friedman *p*-values for all four dimensions are less than 0.05, indicating statistically significant differences among the 12 algorithms at every dimension of CEC 2017.

As shown in Table 8 and Figure 4, MSCPO achieved the best overall mean rank of 2.64, followed by LSHADE-SPACMA (3.11) and L-SHADE (4.37). The original CPO achieved an overall mean rank of 4.49 (5th place), demonstrating that the proposed improvements yield substantial performance gains. Although MSCPO was surpassed by GJA and SSA at D = 100, its strong performance at D = 10, 30, and 50 gave it the best overall ranking on CEC 2017.

### 4.4. Numerical Experiments on CEC 2022

The detailed experimental results on CEC 2022 are reported in Table A10 and Table A11 of Appendix A.

#### 4.4.1. CEC 2022 Wilcoxon Signed-Rank Test

In this section, we applied the non-parametric Wilcoxon signed-rank test to statistically analyze the CEC 2022 results. Detailed experimental results of the Wilcoxon signed-rank test for CEC 2022 are reported in Table A12 and Table A13 of Appendix A. Table 9 compares MSCPO against each competitor on the 12 CEC 2022 functions at D = 10 and D = 20.

(1) At D = 10, MSCPO outperformed (and was inferior to) CPO, GA, DE, WOA, HHO, DBO, GJA, SSA, BBO, L-SHADE, and LSHADE-SPACMA by 6 (2), 12 (0), 7 (3), 12 (0), 11 (0), 11 (0), 11 (0), 11 (0), 10 (1), 3 (2), and 7 (1) functions, respectively, with 4, 0, 2, 0, 1, 1, 1, 1, 1, 7, and 4 draws. Overall, MSCPO won 101 and lost 9 of these pairwise comparisons against all 11 competitors. MSCPO demonstrated complete dominance over GA and WOA (12 wins, 0 losses each), as well as HHO and DBO (11 wins, 0 losses each). The strongest challenges came from L-SHADE (3 wins, 2 losses, 7 draws) and DE (7 wins, 3 losses, 2 draws), whereas LSHADE-SPACMA posed a moderate challenge (7 wins, 1 loss, 4 draws).

(2) At D = 20, MSCPO outperformed (and was inferior to) CPO, GA, DE, WOA, HHO, DBO, GJA, SSA, BBO, L-SHADE, and LSHADE-SPACMA by 7 (0), 12 (0), 9 (2), 12 (0), 12 (0), 12 (0), 9 (1), 11 (0), 8 (1), 5 (4), and 3 (5) functions, respectively, with 5, 0, 1, 0, 0, 0, 2, 1, 3, 3, and 4 draws. Overall, MSCPO won 100 and lost 13 of these comparisons. It was outperformed by LSHADE-SPACMA (3 wins, 5 losses, 4 draws). The reversal against LSHADE-SPACMA at D = 20 reflects the increased complexity of the CEC 2022 functions at higher dimensions, where LSHADE-SPACMA’s covariance matrix adaptation is more effective. MSCPO still dominated GA, WOA, HHO, and DBO (12 wins, 0 losses each), and its advantage over CPO strengthened (7 wins, 0 losses vs. 6 wins, 2 losses at D = 10). L-SHADE narrowed the gap (5 wins, 4 losses, 3 draws vs. 3 wins, 2 losses, 7 draws at D = 10), and BBO became more competitive (8 wins, 1 loss, 3 draws vs. 10 wins, 1 loss, 1 draw at D = 10).

As shown by the “Total 1” columns of Table 9, MSCPO won more comparisons than it lost against all 11 competitors across both dimensions. It achieved overwhelming superiority over GA (24 wins, 0 losses), WOA (24 wins, 0 losses), HHO (23 wins, 0 losses), DBO (23 wins, 0 losses), and SSA (22 wins, 0 losses). It also led GJA (20 wins, 1 loss), BBO (18 wins, 2 losses), and DE (16 wins, 5 losses). Against CPO (13 wins, 2 losses), the advantage was smaller than on CEC 2017, reflecting the increased challenge of the CEC 2022 suite. The most competitive rivals were L-SHADE (8 wins, 6 losses) and LSHADE-SPACMA (10 wins, 6 losses); MSCPO’s advantage over them was marginal and even reversed at D = 20 against LSHADE-SPACMA.

**Figure 5:**
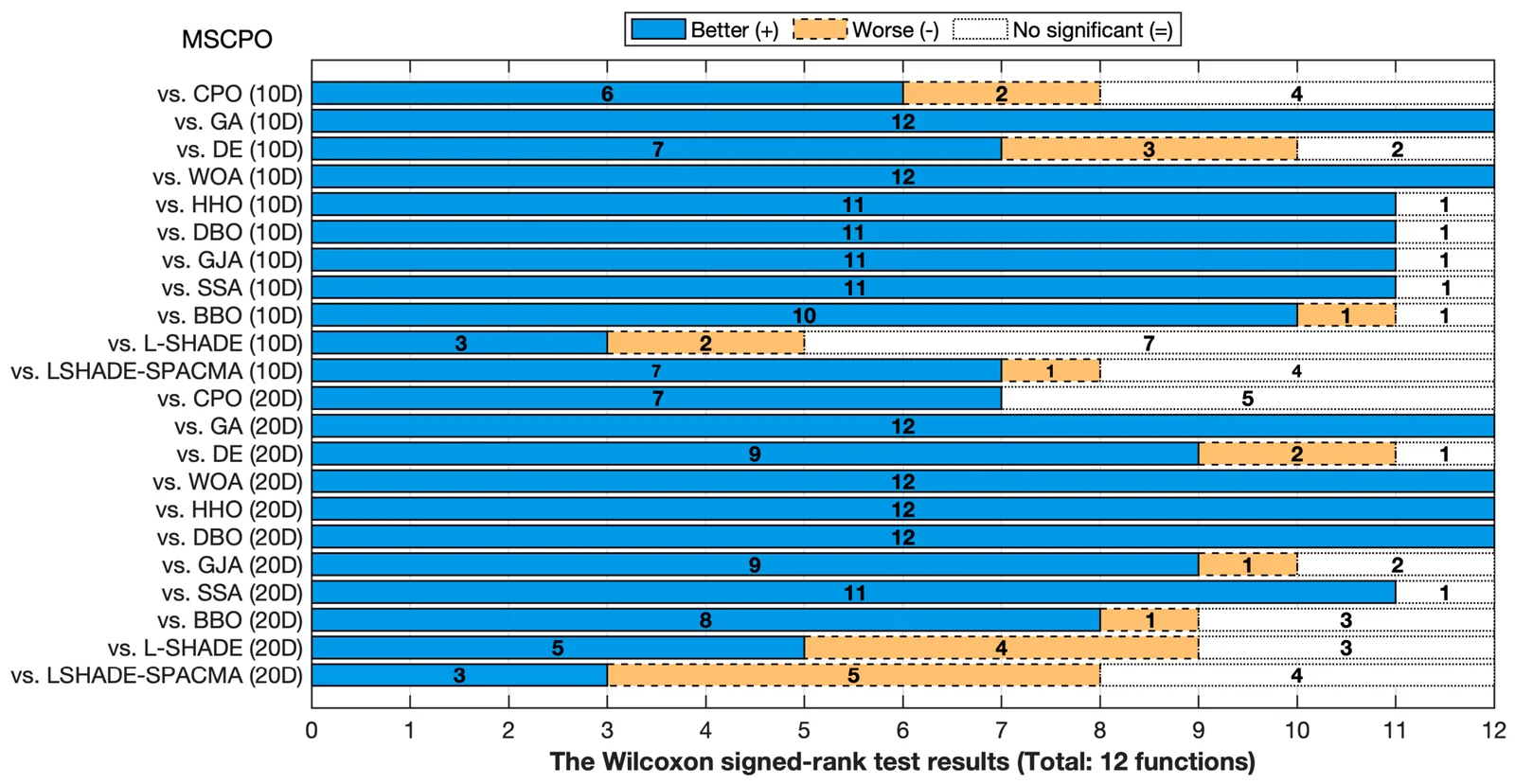
Wilcoxon signed-rank test outcomes (α = 0.05) of MSCPO against each competitor on the 12 CEC 2022 functions (D = 10, 20; 30 runs per function). “+”/“−”: number of functions on which MSCPO is significantly better/worse; “=”; p≥0.05.

The “Total 2” row of Table 9 reveals that MSCPO’s win count remained stable across all dimensions (101 at D = 10 vs. 100 at D = 20), whereas the losses increased from 9 to 13, indicating rising competitive pressure as dimensionality increases. This trend is visually confirmed by Figure 5. Overall, the proposed MSCPO won 201, lost 22, and drew 41 of the 264 pairwise comparisons. This demonstrates the robustness and effectiveness of MSCPO on CEC 2022, although LSHADE-SPACMA proved to be a strong competitor at D = 20.

#### 4.4.2. CEC 2022 Friedman Mean Rank Test

**Figure 6:**
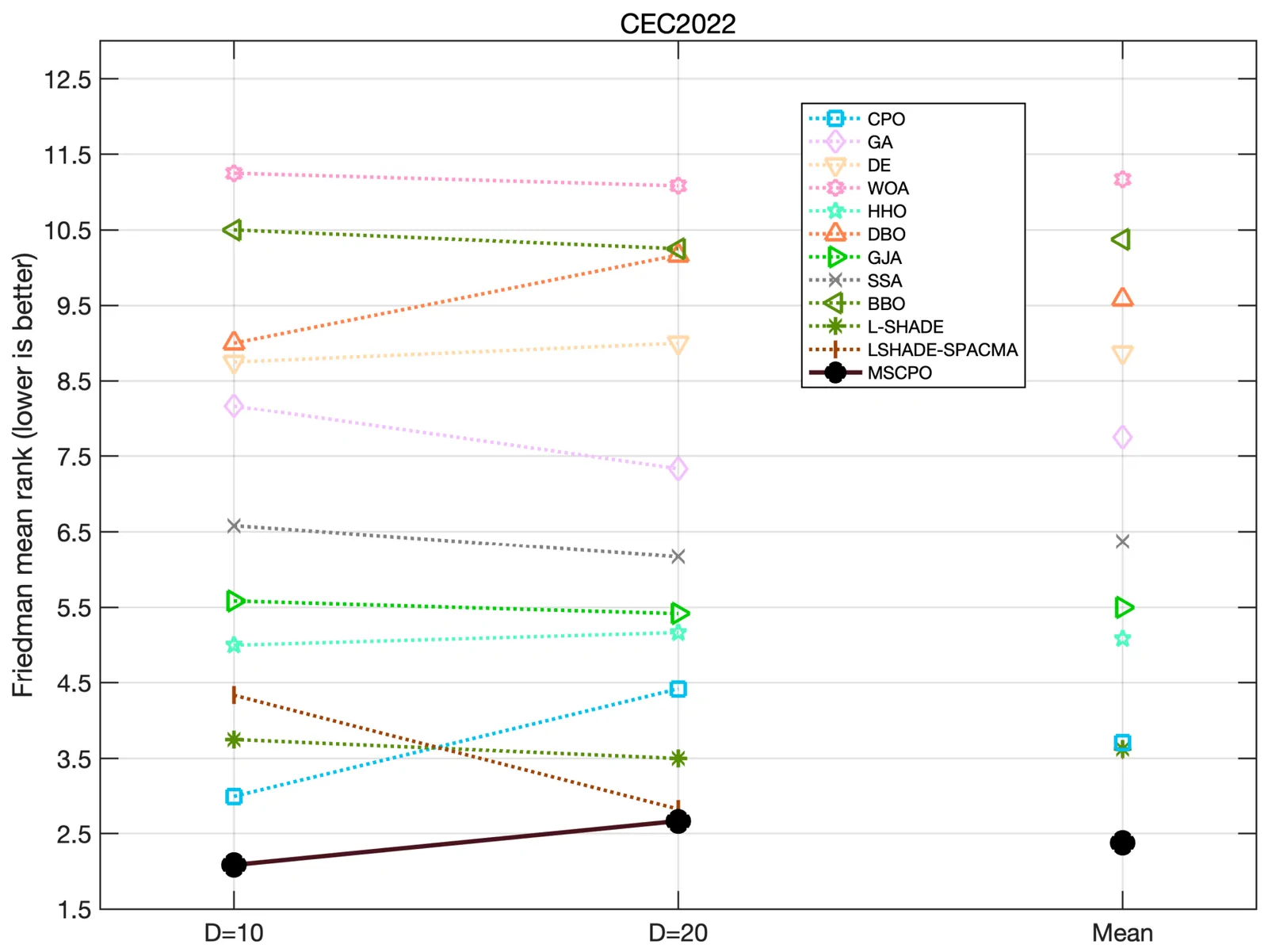
Friedman mean ranks (lower is better) on the 12 CEC 2022 functions (D = 10, 20; 30 runs per function). Ranks are computed per function and averaged over all functions; “Mean” denotes the average over the two dimensions; each dimension is an independent experiment.

In this section, we applied the Friedman mean rank test to evaluate the results on the CEC 2022 test suite. Table 10 presents the Friedman mean rank test results at D = 10 and D = 20. The Friedman *p*-values are 6.94 × 10^−15^ (D = 10) and 5.88 × 10^−15^ (D = 20), both well below α = 0.05, confirming statistically significant performance differences among the 12 algorithms. Figure 6 visualizes the ranking comparison between MSCPO and its competitors.

At D = 10, MSCPO ranked first (mean rank 2.08), outperforming all 11 competitors. CPO ranked second (mean rank 3.00), followed by L-SHADE (mean rank 3.75) and LSHADE-SPACMA (mean rank 4.33), indicating that MSCPO’s strategies provide decisive advantages on the compact yet challenging CEC 2022 functions at low dimensionality. At D = 20, MSCPO again ranked first (mean rank 2.67), surpassing the other 11 competitors. LSHADE-SPACMA improved substantially to second place (mean rank 2.83, gap = 0.16), making this the most competitive dimension between the top two algorithms. L-SHADE held third place (mean rank 3.50), while CPO dropped to fourth (mean rank 4.42). The close competition between MSCPO and LSHADE-SPACMA at D = 20 reflects the increased complexity of the shifted and rotated functions in CEC 2022, on which LSHADE-SPACMA’s semi-parameter adaptation is more effective.

Combining the results of Table 10 and Figure 6, we find that MSCPO achieved the best overall mean rank of 2.375, followed by LSHADE-SPACMA (mean rank 3.583) and L-SHADE (mean rank 3.625). The original CPO achieved an overall mean rank of 3.708 (4th place). MSCPO maintained first place on CEC 2022, with its largest advantage at D = 10 and a narrower but preserved advantage at D = 20.

## 5. Application in Engineering Optimization Problems

In this section, MSCPO was evaluated on three engineering design problems: the step-cone pulley design problem [35,45], the hydrostatic thrust bearing design problem [35,46], and the robotic gripper design [35,47]. For these constrained problems, a larger population (N=100) with a smaller iteration budget ($T_{max}=500$) was adopted, since each evaluation involves multiple nonlinear constraint terms and is therefore more expensive than a benchmark-function evaluation. MSCPO and all competitors were each run independently 30 times under the identical configuration, so the comparison remains internally fair.

### 5.1. Step-Cone Pulley Design

**Figure 7:**
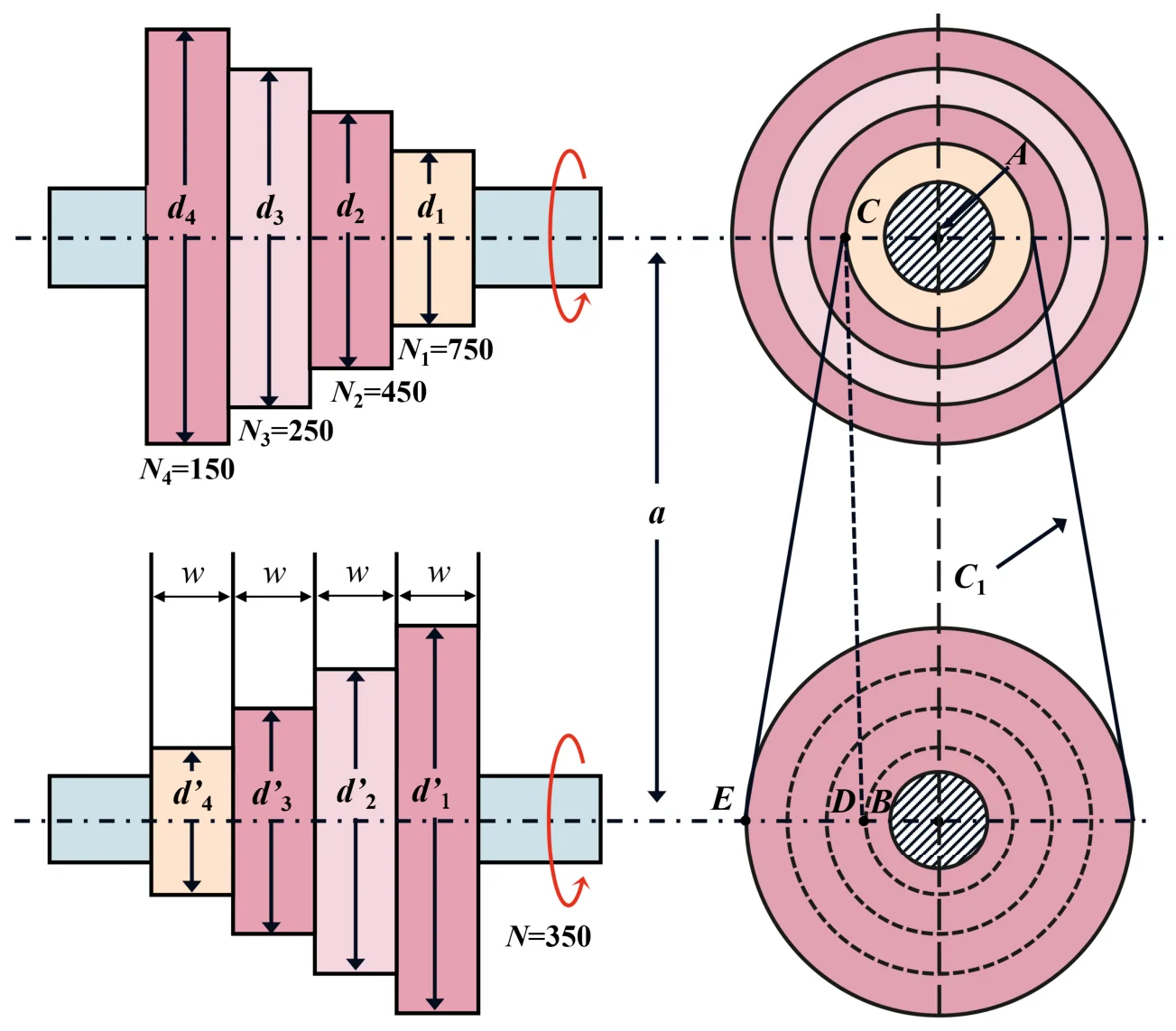
Mechanical structure of the step-cone pulley system.

The step-cone pulley design problem is a classical benchmark in engineering optimization. It aims to minimize the mass of a four-stage pulley assembly. The problem involves five design variables: the diameters of the four steps and the common pulley width. It is subject to 11 nonlinear constraints, including the requirement of transmitting 0.75 horsepower. The mechanical structure of the step-cone pulley system is depicted in Figure 7.

The mathematical model is formulated as follows:

Minimize:

(29) $$f\left(x\right)=\rho \omega \left[d_{1}^{2}\left\{1+{\left(\frac{N_{1}}{N}\right)}^{2}\right\}+d_{2}^{2}\left\{1+{\left(\frac{N_{2}}{N}\right)}^{2}\right\}+d_{3}^{2}\left\{1+{\left(\frac{N_{3}}{N}\right)}^{2}\right\}+d_{4}^{2}\left\{1+{\left(\frac{N_{4}}{N}\right)}^{2}\right\}\right]$$

Subject to:

$h_{1}\left(x\right)=C_{1}-C_{2}=0$, $h_{2}\left(x\right)=C_{1}-C_{3}=0$, $h_{3}\left(x\right)=C_{1}-C_{4}=0$, $g_{1,2,3,4}\left(x\right)=R_{i}\geq 2$, $g_{5,6,7,8}\left(x\right)=P_{i}\geq \left(0.75\;\times \;745.6998\right)$, where $C_{i}$ indicates the length of the belt to obtain speed $N_{i}$ and is given by

$$C_{i}=\frac{\pi d_{i}}{2}\left(1+\frac{N_{i}}{N}\right)+\frac{{\left(\frac{N_{i}}{N}-1\right)}^{2}}{4a}+2a,\;i=\left(1,2,3,4\right).$$

$R_{i}$ is the tension ratio and is given by

$$R_{i}=exp\left[\mu \left\{\pi -2{sin}^{-1}\left\{\left(\frac{N_{i}}{N}-1\right)\frac{d_{i}}{2a}\right\}\right\}\right],\;i=\left(1,2,3,4\right).$$

$P_{i}$ is the power transmitted at each step

$$P_{i}=stw\left[1-exp\left[-\mu \left\{\pi -2{sin}^{-1}\left\{\left(\frac{N_{i}}{N}-1\right)\frac{d_{i}}{2a}\right\}\right\}\right]\right]\frac{\pi d_{i}N_{i}}{60},\;i=\left(1,2,3,4\right).$$

$$\rho =7200\;kg/m^{3},\;a=3\;m,\;\mu =0.35,\;s=1.75\;MPa,\;t=8\;mm.$$

**Figure 8:**
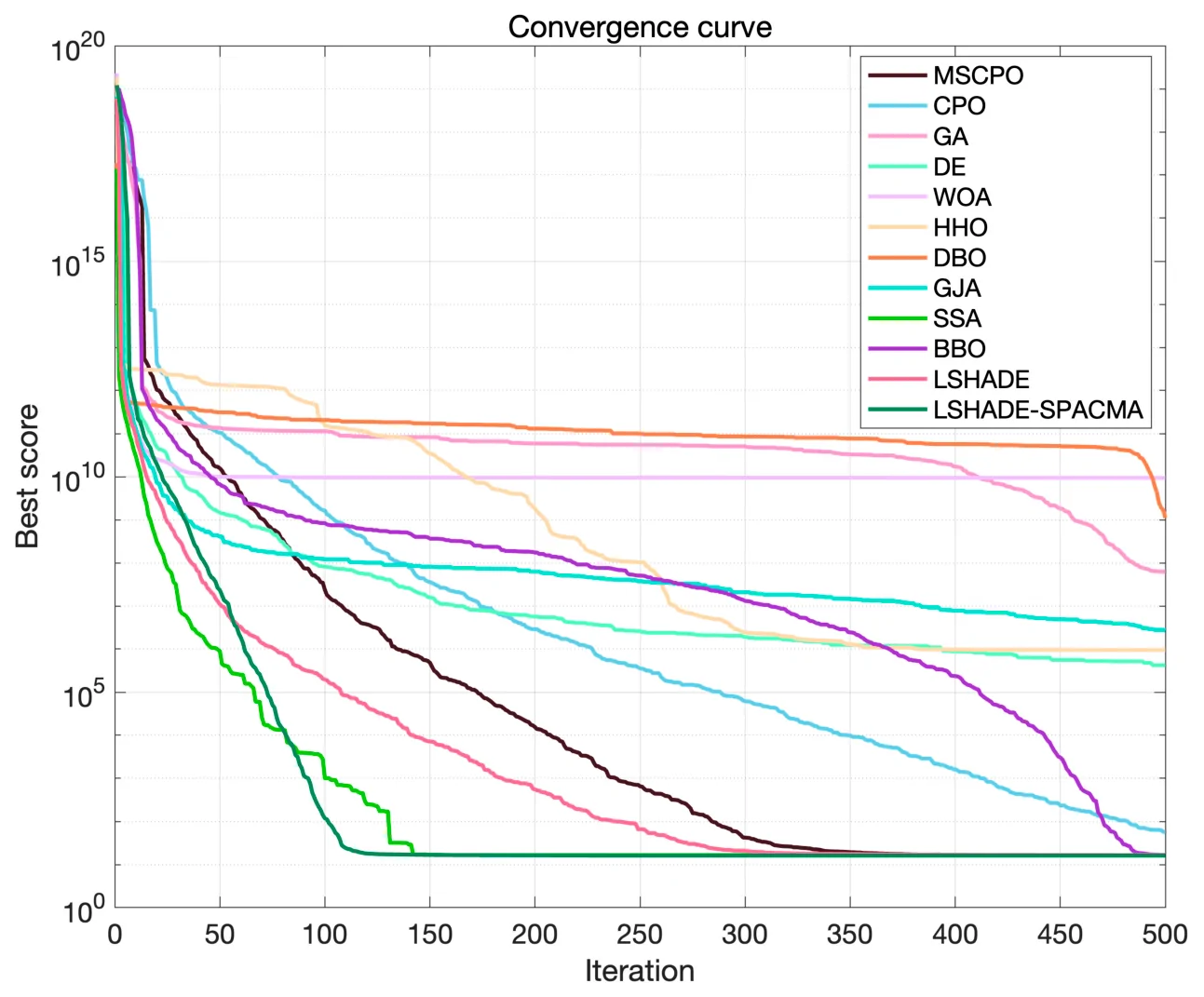
Average convergence curve for the step-cone pulley design problem.

**Figure 9:**
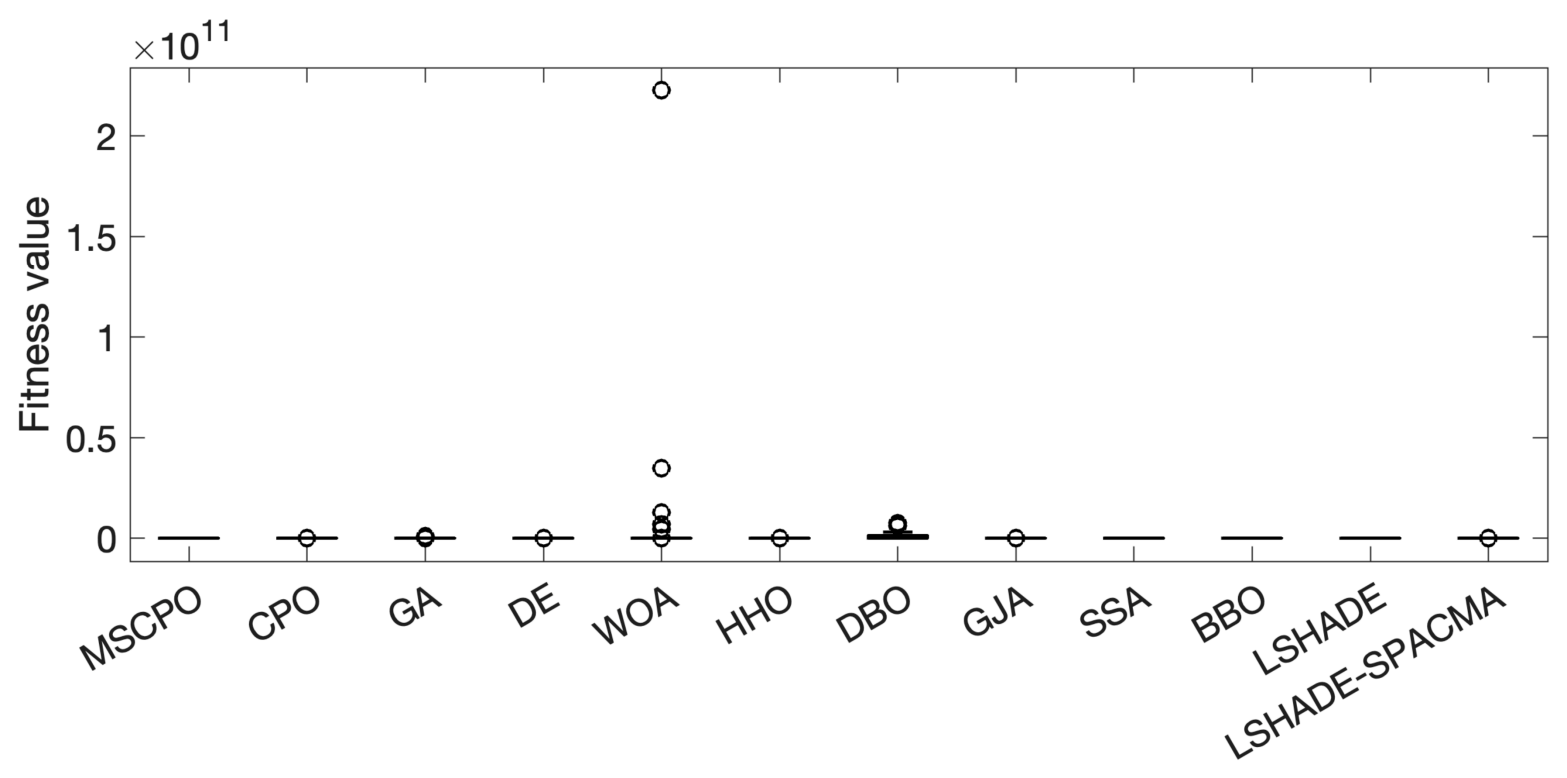
Box plot for the step-cone pulley design problem.

The proposed MSCPO algorithm was applied to the step-cone pulley design problem and compared with CPO, WOA, HHO, GA, DE, DBO, BBO, GJA, SSA, L-SHADE, and LSHADE-SPACMA. The optimal parameters and statistical results obtained by the different algorithms are presented in Table 11 and Table 12. The average convergence curve and box plot of the step-cone pulley design are given in Figure 8 and Figure 9.

As presented in Table 11, LSHADE-SPACMA obtained the lowest optimal cost (16.09), followed closely by MSCPO (16.16); the gap between the two is only about 0.4%, and both are clearly better than all other competitors. MSCPO also markedly improves over the original CPO (18.41), confirming its capability in identifying near-global optima for this constrained problem. The convergence behavior illustrated in Figure 8 reveals that MSCPO converges rapidly and stabilizes at the optimal value with negligible fluctuations, indicating an effective balance between global exploration and local exploitation. In contrast, DBO, WOA, GA, GJA, HHO, and DE show slower convergence or premature stagnation at higher objective values.

The statistical metrics in Table 12 and the box plot in Figure 9 provide insights into algorithmic robustness. LSHADE-SPACMA is almost perfectly stable on this problem, reaching 16.09 in every run (Std = 2.40 × 10^−7^). MSCPO attains the second-best mean (16.47) and the second-smallest standard deviation (0.09), with a worst-case value of 16.68, ahead of SSA (mean 16.58) and L-SHADE (mean 16.62). This consistency across multiple independent runs highlights its reliability and low sensitivity to initial conditions. In contrast, WOA, DBO, and GA exhibit large variance and poor worst-case results, indicating instability and a higher risk of convergence failure. As shown in Table 11, the design variables ($d_{1}-d_{4},w$) obtained by MSCPO fall within reasonable engineering limits. Crucially, this parameter set satisfies all 11 nonlinear constraints, thereby confirming its feasibility. These results highlight the practical utility of the method in generating viable engineering solutions.

In summary, on the step-cone pulley design problem, MSCPO ranks second overall, behind only LSHADE-SPACMA, and first among all nature-inspired competitors, clearly improving over the original CPO in both accuracy and robustness. WOA, GA, DE, DBO, and GJA yield inferior or highly unstable results, underscoring their inadequacy for handling such complex and constrained problems. These findings validate the efficacy of the improved strategies in MSCPO.

### 5.2. Hydrostatic Thrust Bearing Design

**Figure 10:**
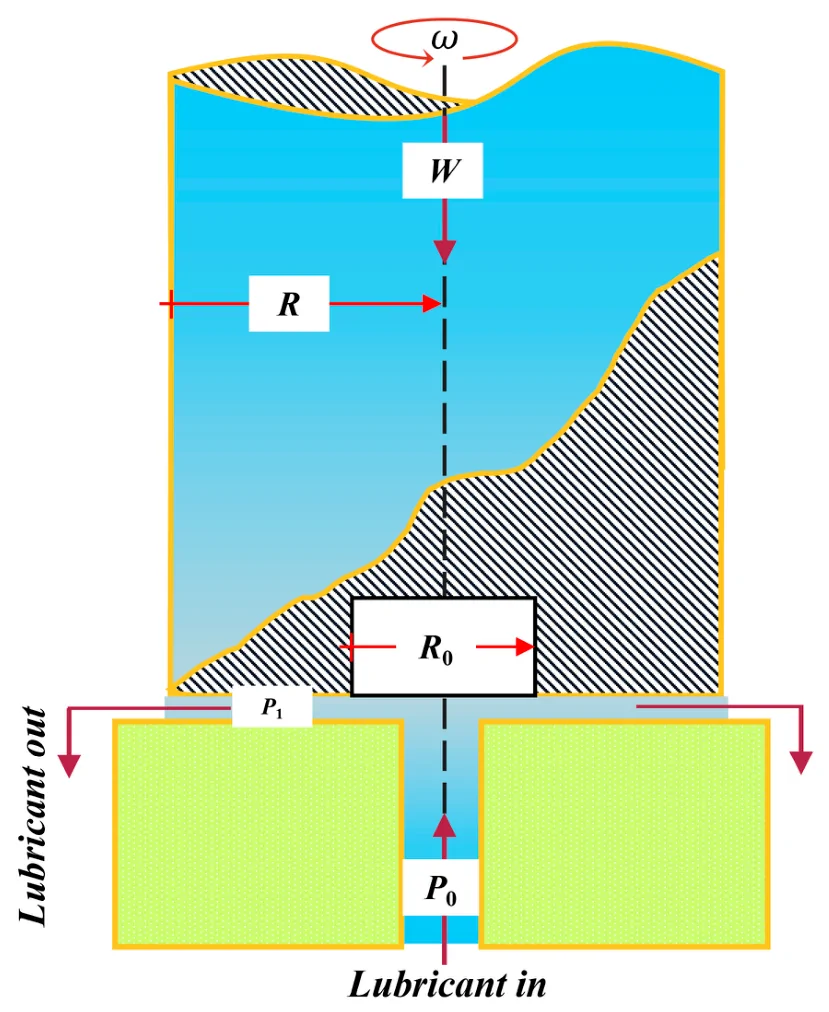
Schematic of the hydrostatic thrust bearing design.

The hydrostatic thrust bearing design problem aims to minimize the total power loss during operation. It has four design variables: the bearing step radius R, the recess radius $R_{0}$, the oil viscosity μ, and the lubricant flow rate Q. The system must satisfy seven nonlinear constraints related to inlet oil pressure, load capacity, oil film thickness, and other critical performance criteria. The configuration of the bearing is illustrated in Figure 10. The mathematical model is formulated as follows.

Minimize:

(30) $$f\left(x\right)=\frac{QP_{0}}{0.7}+E_{f}$$

Subject to:

$$g_{1}\left(x\right)=W-W_{S}\geq 0,\;g_{2}\left(x\right)={P_{max}-P}_{0}\geq 0,\;g_{3}\left(x\right)={\Delta T}_{max}-\Delta T\geq 0,\;g_{4}\left(x\right)=h-h_{min}\geq 0,\;g_{5}\left(x\right)={R-R}_{0}\geq 0,\;g_{6}\left(x\right)=0.001-\frac{\gamma }{gP_{0}}\left(\frac{Q}{2\pi Rh}\right)\geq 0,\;g_{7}\left(x\right)=5000-\frac{W}{\pi \left(R^{2}-R_{0}^{2}\right)}\geq 0,$$

where

$$W=\frac{\pi P_{0}}{2}\frac{R^{2}-R_{0}^{2}}{ln\left(\frac{R}{R_{0}}\right)},\;P_{0}=\frac{6\mu Q}{\pi h^{3}}ln\left(\frac{R}{R_{0}}\right),\;E_{f}=9336Q\gamma C\Delta T,\;\Delta T=2\left(10^{P}-560\right),\;P=\frac{{log}_{10}{log}_{10}\left(8.122\times 10^{6}\mu +0.8\right)-c_{1}}{n},\;h={\left(\frac{2\pi N}{60}\right)}^{2}\left(\frac{2\pi \mu }{E_{f}}\right)\left(\frac{R^{4}-R_{0}^{4}}{4}\right)$$

with

$$\gamma =0.0307,\;C=0.5,\;n=-3.55,\;c_{1}=10.04,\;W_{S}=10,100,\;P_{max}=1000,\;{\Delta T}_{max}=50,\;h_{min}=0.001,\;g=386.4,\;N=750.\;1\leq R,\;R_{0},\;Q\leq 16,\;10^{-6}\leq \mu \leq 16\times 10^{-6}.$$

**Figure 11:**
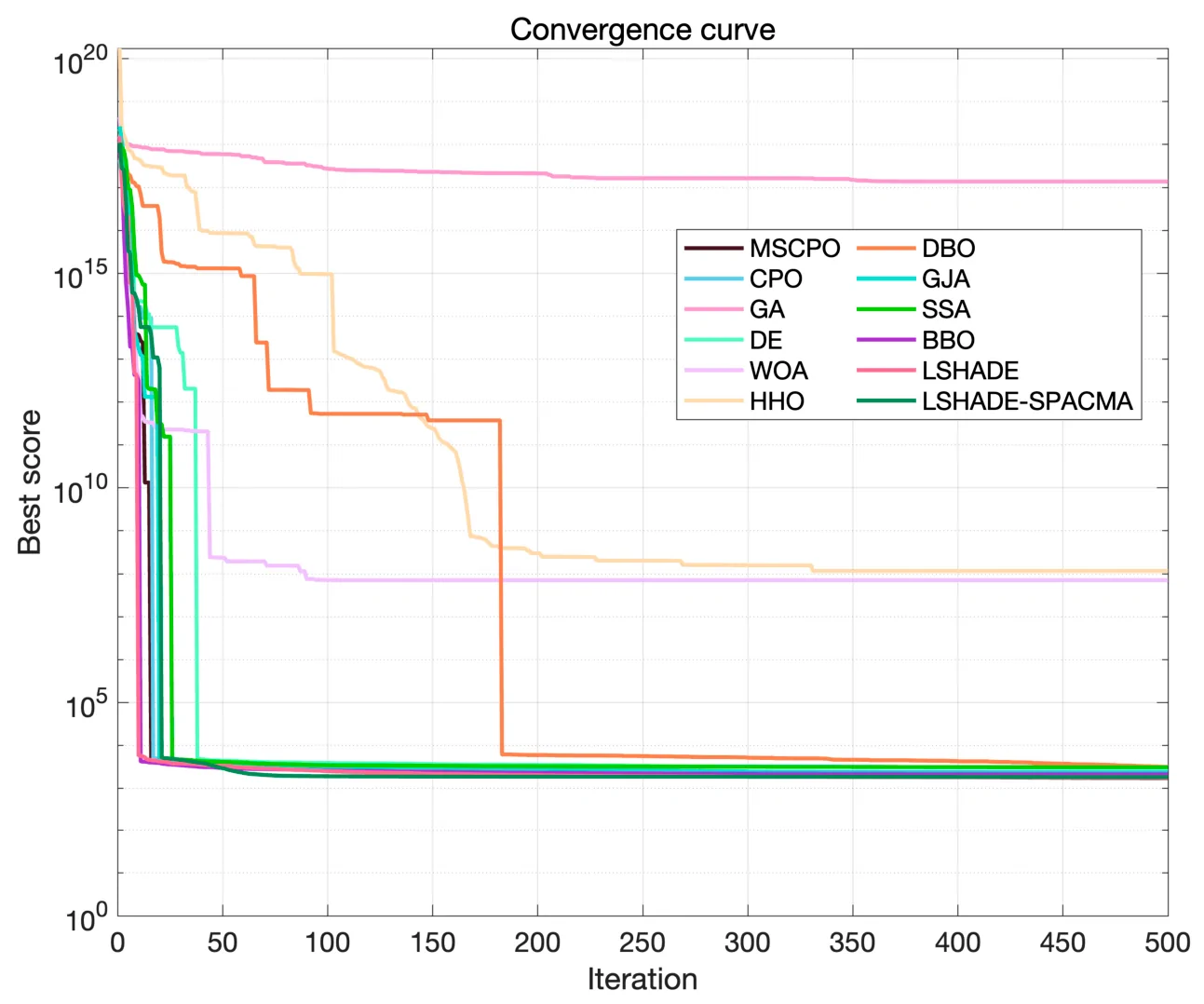
Average convergence curve for the hydrostatic thrust bearing design problem.

**Figure 12:**
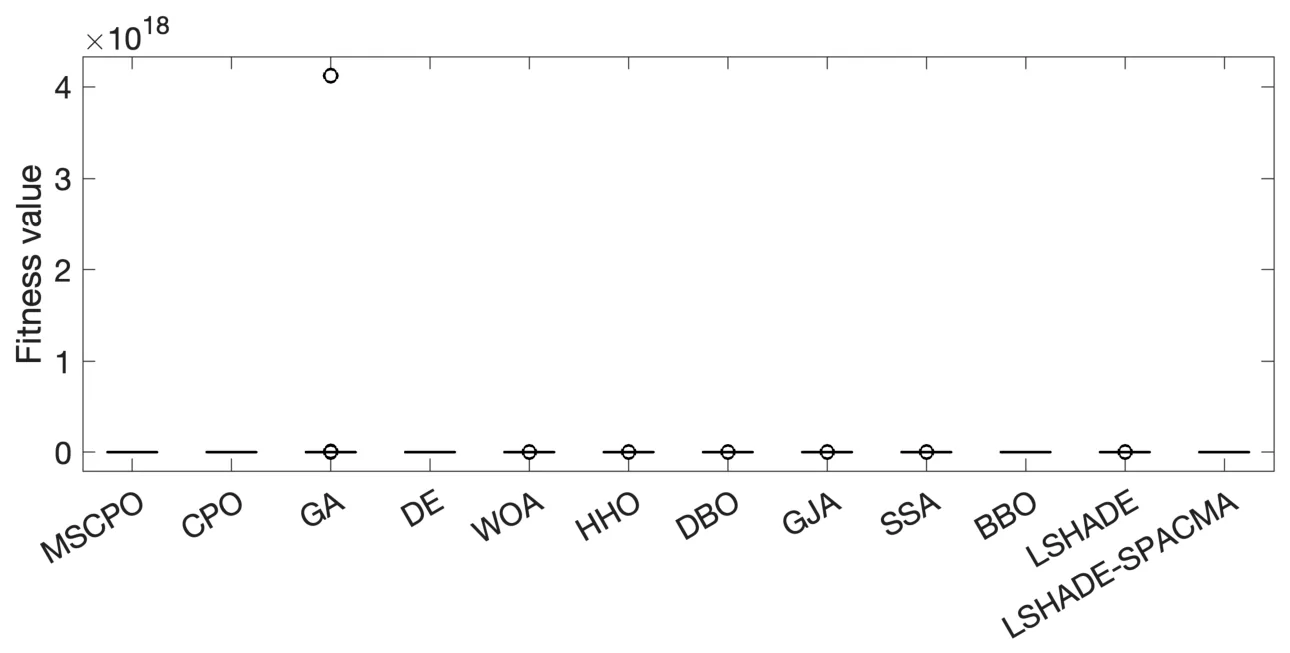
Box plot for the hydrostatic thrust bearing design problem.

Table 13 and Table 14 report the optimal designs and the corresponding statistical results, and Figure 11 and Figure 12 show the convergence curves and box plots.

As recorded in Table 13, MSCPO achieves the lowest minimum power loss (1632.34) among all twelve algorithms, slightly ahead of LSHADE-SPACMA (1633.25) and L-SHADE (1637.26). The convergence curve in Figure 11 shows that MSCPO, CPO, DE, GJA, SSA, BBO, L-SHADE, and LSHADE-SPACMA all exhibit a sharp decline in the objective value during the initial search phase, stabilizing after approximately 25 iterations. MSCPO attains a consistently lower best value, indicating better refinement near the optimum. In contrast, GA, WOA, HHO, and DBO display slower convergence or apparent convergence to suboptimal plateaus.

The statistical metrics in Table 14 and the box plot in Figure 12 demonstrate that MSCPO achieves the lowest mean power loss (1730.27), the smallest standard deviation (61.02), and the best worst-case value (1839.35) among all compared algorithms, including the two CEC competition algorithms L-SHADE (mean 1774.11, Std 61.72) and LSHADE-SPACMA (mean 1822.31, Std 105.84), indicating consistent performance across multiple trials. In contrast, algorithms such as GA and HHO show higher variance and degraded worst-case performance, reflecting sensitivity to initialization or parameter settings. The optimal design vector obtained by MSCPO (R=5.97, $R_{0}=5.40$, $\mu =5.40\times 10^{-6}$, and Q=2.31) corresponds to physically realistic dimensions and operating conditions. This configuration satisfies all seven nonlinear constraints, confirming that the solution is both optimal and feasible for the intended application.

### 5.3. Robotic Gripper Design

**Figure 13:**
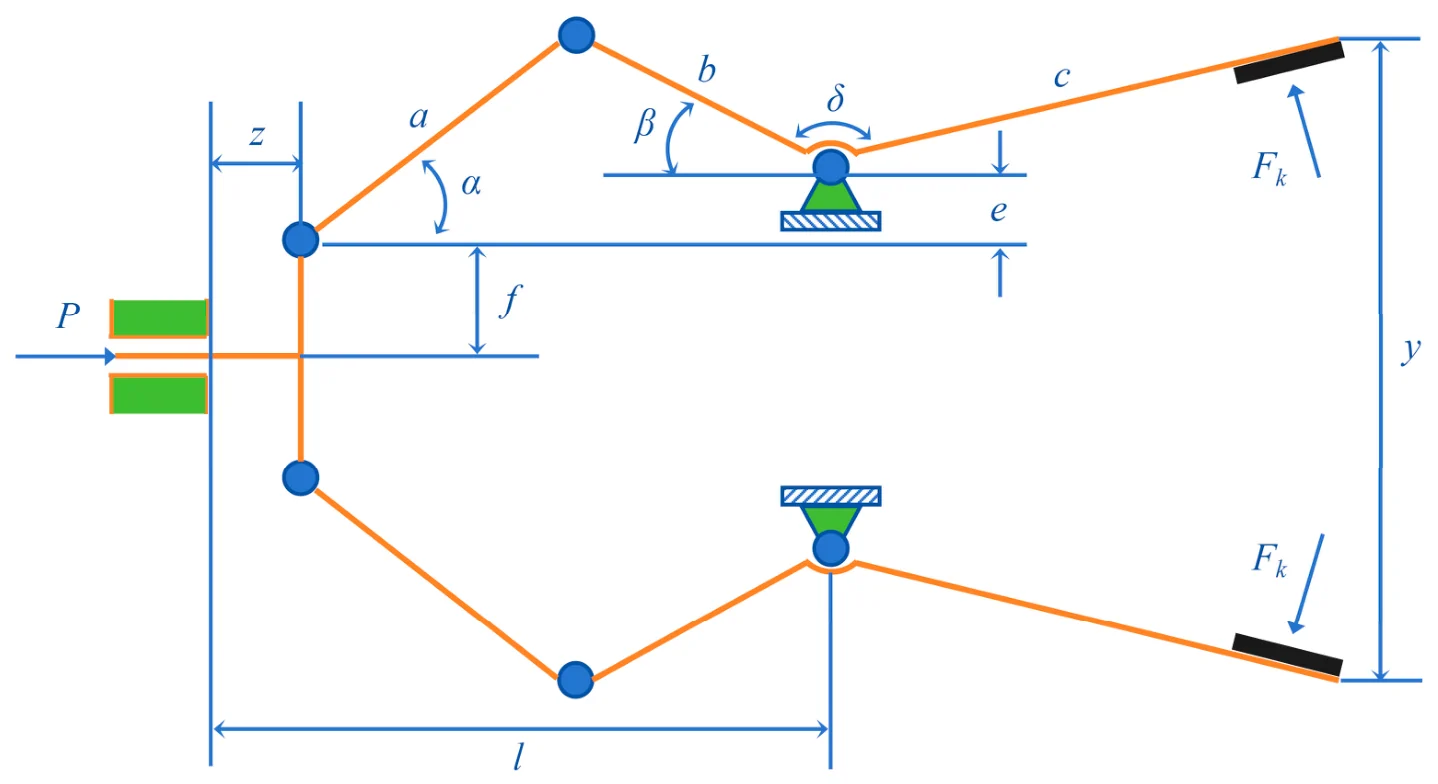
Schematic of the robotic gripper mechanism.

The robotic gripper design problem is a classical mechanical optimization problem. Its objective is to minimize the difference between the maximum and minimum gripping forces. The structure of the gripper mechanism is illustrated in Figure 13. The problem involves seven design variables related to the gripper mechanism and seven nonlinear design constraints. The mathematical model is formulated as follows.

Minimize:

(31) $$f\left(x\right)=\underset{z}{\max}F_{k}\left(x,z\right)-\underset{z}{\min}F_{k}\left(x,z\right)$$

Subject to:

$$g_{1}\left(x\right)=Y_{min}-y\left(x,z_{max}\right)\geq 0,\;g_{2}\left(x\right)=y\left(x,z_{max}\right)\geq 0,\;g_{3}\left(x\right)={y\left(x,0\right)-Y}_{max}\geq 0,\;g_{4}\left(x\right)=Y_{G}-y\left(x,0\right)\geq 0,\;g_{5}\left(x\right)={\left(a+b\right)}^{2}-l^{2}-e^{2}\geq 0,\;g_{6}\left(x\right)={\left(l-z_{max}\right)}^{2}+{\left(a-e\right)}^{2}-b^{2}\geq 0,\;g_{7}\left(x\right)=l-z_{max}\geq 0,$$

where

$$g=\sqrt{{\left(l-z\right)}^{2}+e^{2}},\;\alpha =arccos\left(\frac{a^{2}+g^{2}-b^{2}}{2ag}\right)+\Phi ,\;\beta =arccos\left(\frac{b^{2}+g^{2}-a^{2}}{2bg}\right)-\Phi ,\;\Phi =arctan\left(\frac{e}{l-z}\right),\;F_{k}=\frac{Pbsin\left(\alpha +\beta \right)}{2ccos\left(\alpha \right)},\;y\left(x,z\right)=2\left(e+f+csin\left(\beta +\delta \right)\right).$$

$$Y_{min}=50,\;Y_{max}=100,\;Y_{G}=150,\;z_{max}=100,\;P=100,\;10\leq f,\;a,\;b\leq 150,\;100\leq c\leq 200,\;0\leq e\leq 50,\;100\leq l\leq 300,\;1\leq \delta \leq 3.14.$$

**Figure 14:**
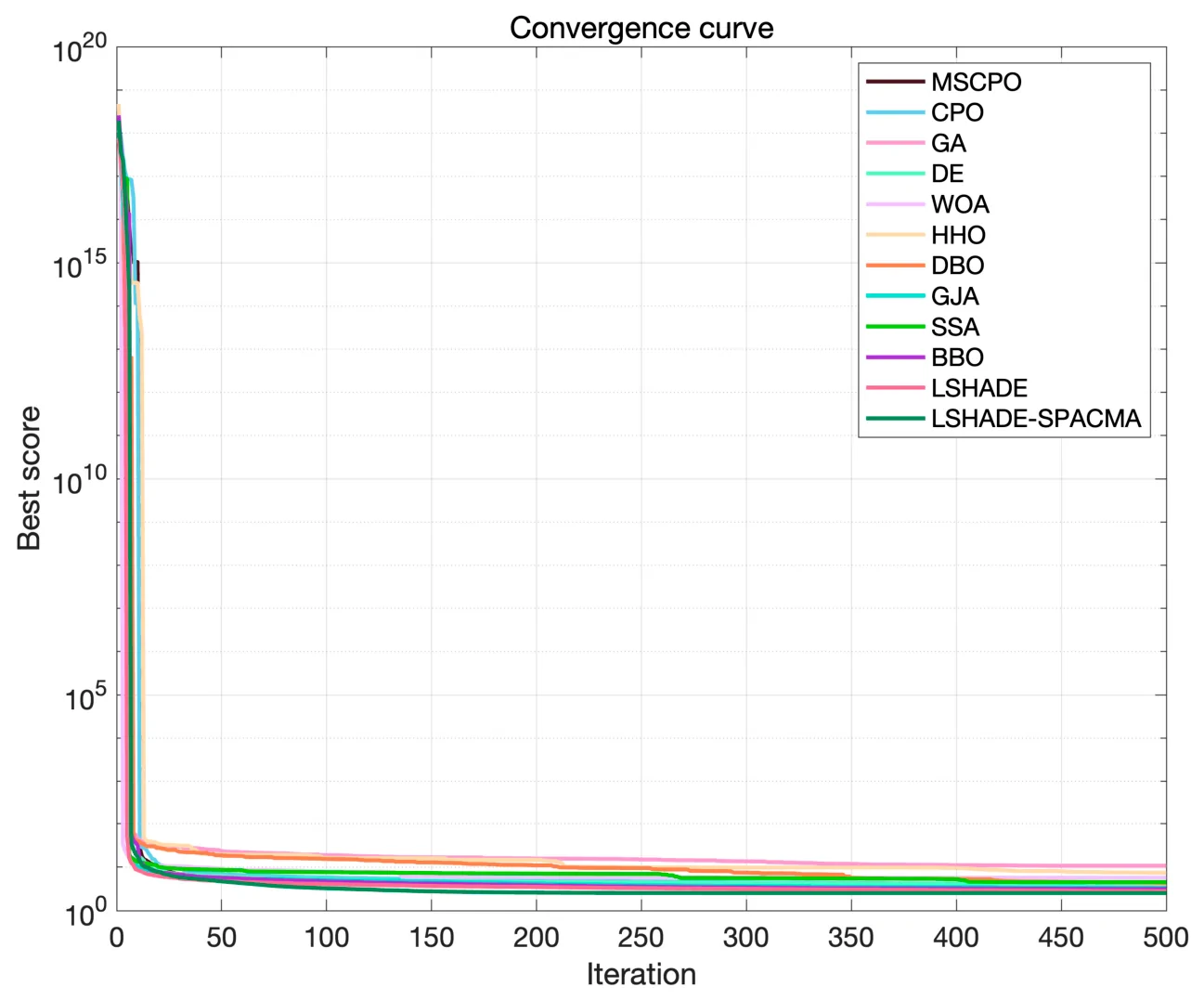
Average convergence curve for the robotic gripper design problem.

**Figure 15:**
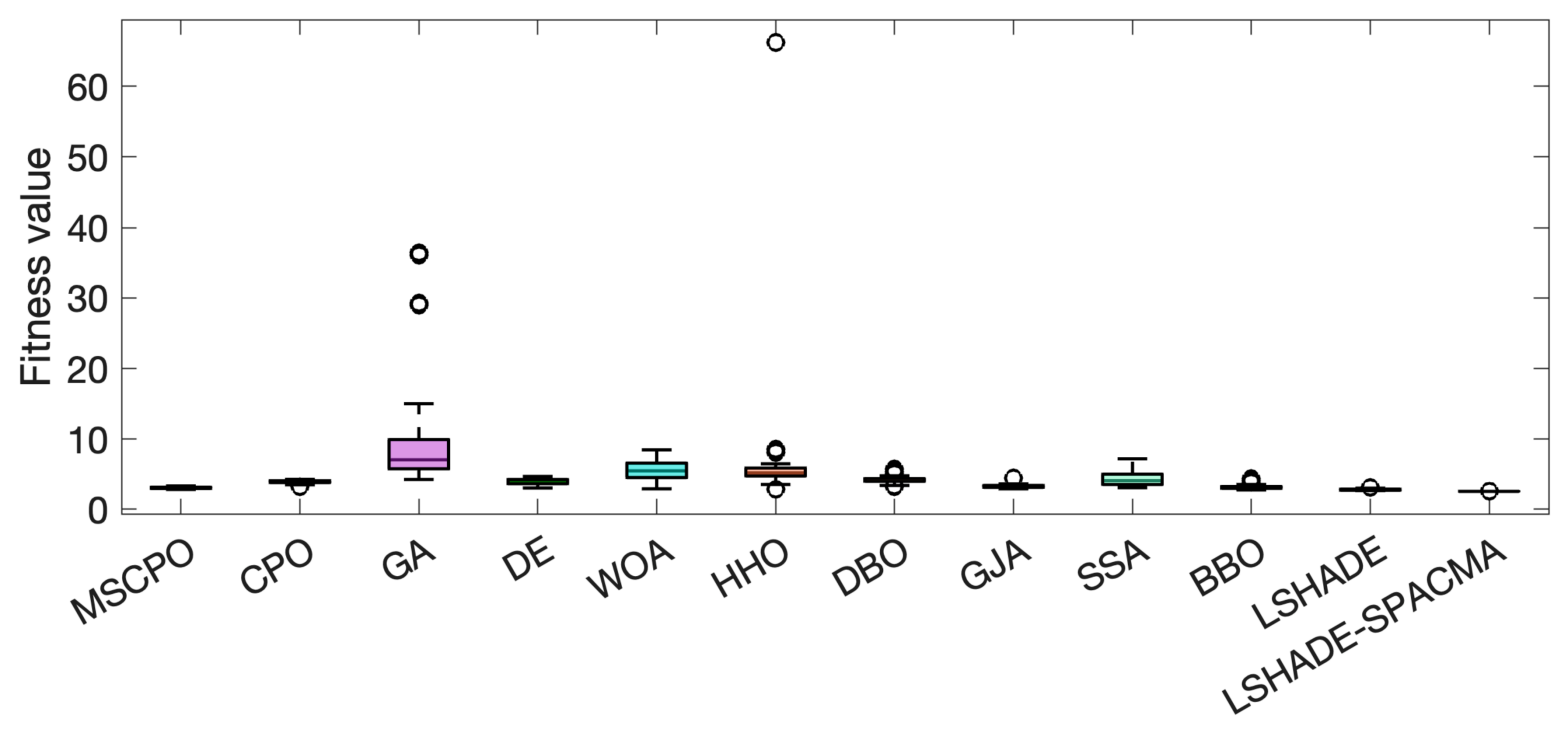
Box plot for the robotic gripper design problem.

The proposed MSCPO algorithm was applied to the robotic gripper design problem. The optimal parameters and statistical results are listed in Table 15 and Table 16. The average convergence curve and box plot of the robotic gripper design are given in Figure 14 and Figure 15.

The optimal solutions obtained by all tested algorithms are recorded in Table 15. LSHADE-SPACMA obtains the lowest best value (2.5438), followed by L-SHADE (2.6502) and MSCPO (2.7312), with BBO (2.7965) close behind; the gap between MSCPO and the best value is within 8%. MSCPO clearly outperforms the original CPO (3.2382) as well as GA, DE, DBO, and SSA, confirming its strong optimization capability for this constrained mechanical design problem.

As shown in Figure 14, all tested algorithms exhibit a rapid initial reduction in the objective value and stabilize after approximately 100 iterations. The final converged values of the top performers differ only by a narrow margin: LSHADE-SPACMA and L-SHADE reach slightly lower final values, while MSCPO converges to a value close to the two CEC competition algorithms and clearly lower than the other nine competitors, including the original CPO.

The statistical results in Table 16 and the distribution of solutions visualized in Figure 15 demonstrate that LSHADE-SPACMA, L-SHADE, and MSCPO are the only three algorithms that combine low mean objective values with small standard deviations on this problem. LSHADE-SPACMA achieves the lowest mean (2.5439) and standard deviation (5.81 × 10^−4^), followed by L-SHADE (mean 2.7846, Std 0.1245) and MSCPO (mean 3.0344, Std 0.1277), which is ahead of BBO (3.1662) and clearly better than the original CPO (3.9336). In contrast, HHO and GA show large fluctuations and a higher worst-case objective value. The optimal design variables obtained by MSCPO all fall within the specified variable bounds. More importantly, this design strictly satisfies all seven nonlinear constraints, confirming that the optimal solution is both mechanically feasible and compliant with the problem’s performance requirements.

## 6. Discussion

### 6.1. Practical Efficiency

The asymptotic analysis in Section 3.8 shows that MSCPO preserves the time-complexity order of the original CPO, with only a constant-factor overhead. To verify this in practice, Table 17 compares the measured wall-clock runtimes of the two algorithms on all benchmark suites and engineering problems considered in this paper.

As shown in Table 17, the runtime ratio of MSCPO to CPO ranges from 1.13 to 1.44 across all benchmark configurations and, importantly, decreases as the problem dimension grows, confirming that the overhead is a small, dimension-independent constant factor. The ratios on the three engineering problems (1.18, 1.20, and 1.03 for the step-cone pulley, hydrostatic thrust bearing, and robotic gripper problems, respectively) fall within the same range. In addition, the extra N objective evaluations consumed by KCOL at initialization account for only about 0.1–0.2% of the total evaluation budget, as quantified in Section 3.8. The measured runtimes thus corroborate the complexity analysis: the performance gains of MSCPO reported in Section 4 and Section 5 are obtained at an acceptable, constant-factor runtime cost.

### 6.2. Comparison with Prior CPO Variants

Several enhanced variants of CPO have been proposed recently. ECPO [18] combines Sobol sequence initialization with a guided search strategy, an adaptive Levy flight, and centroid-based opposition-based learning; BTCPO [36] integrates a butterfly search strategy with a triangular walk; CAPCPO [38] introduces a composite Cauchy mutation strategy, an adaptive dynamic adjustment strategy, and a population mutation strategy; and SDHCPO [48] combines Sobol-OBL initialization, cosine annealing, a DE/rand/1 mutation, and horizontal–vertical crossover. The mechanism-level comparison is shown in Table 18. A distinguishing feature of MSCPO is its phase-targeted design: each of the four strategies is assigned to a specific phase of the original CPO (the initialization stage and the first, second, and fourth defense phases), so that the enhancements address four distinct weaknesses rather than modifying a single operator. This one-to-one correspondence also allows the contribution of each strategy to be analyzed independently, as demonstrated by the ablation study in Section 4.2.

A direct or indirect numerical comparison between MSCPO and these variants is not feasible. As shown in Table 18, these variants were evaluated under substantially different configurations: ECPO was compared with 14 algorithms, including five CEC competition winners, on the CEC 2014, CEC 2017, CEC 2020, and CEC 2022 suites with a maximum of 500 iterations; BTCPO was compared with 8 algorithms on 23 classical benchmark functions and the CEC 2021 suite; CAPCPO was compared with 11 algorithms on the CEC 2017, CEC 2019, and CEC 2022 suites with a maximum of 500 iterations; and SDHCPO was compared with 7 algorithms on the CEC 2017 (D = 30 and 50) and CEC 2022 (D = 20) suites with a maximum of 500 iterations. Although partial overlaps with our testbed exist (CEC 2017 at D = 30 in ECPO and CAPCPO, and CEC 2022 at D = 10 and 20 in CAPCPO and at D = 20 in SDHCPO), the iteration budgets (500 versus 1000 iterations) and the comparison sets differ, and none of these studies reports results under the full configuration adopted in this paper (CEC 2017 at D = 10, 30, 50, and 100, and CEC 2022 at D = 10 and 20, with 1000 iterations). A direct comparison would therefore require re-implementing and carefully re-tuning each variant under a unified protocol, which falls outside the scope of this study. An indirect comparison through common baselines is likewise unreliable, because the absolute performance of any shared baseline itself depends on the evaluation budget. The comparison in this section is therefore restricted to the mechanism level. In this respect, the strategies of these variants are largely complementary to those of MSCPO, and combining them with the phase-targeted framework of MSCPO is a promising direction for future CPO enhancement.

### 6.3. Limitations and Generalizability

Several limitations of this study should be acknowledged. First, the advantage of MSCPO is dimension-dependent: on CEC 2017 it ranks first at D = 10, 30, and 50 but drops to third at D = 100, behind GJA and SSA; on CEC 2022 at D = 20, it records more losses than wins against LSHADE-SPACMA in the pairwise Wilcoxon comparison despite a better overall mean rank. A plausible explanation is that, with a fixed initial population size and a cyclic reduction schedule that does not adapt to the problem dimension, the four strategies may not fully exploit high-dimensional search spaces; this hypothesis remains to be tested. The attenuation is unlikely to stem from computational cost, because the runtime overhead of MSCPO relative to CPO decreases as the dimension grows, as shown in Table 17. Second, on the engineering problems, MSCPO is outperformed by LSHADE-SPACMA on the step-cone pulley and robotic gripper problems (and also by L-SHADE on the latter), suggesting that covariance matrix adaptation and success-history-based parameter adaptation are particularly effective on these highly constrained problems. Third, MSCPO has so far been evaluated only on unconstrained single-objective benchmark functions and three constrained engineering problems handled by a static penalty approach; its generalizability to other problem classes, such as dynamic, multi-modal constrained, or many-objective problems, remains to be verified. Finally, although KCOL is designed to improve the diversity and boundary coverage of the initial population, this coverage gain has not yet been quantified with discrepancy-based measures, and a systematic comparison with alternative schemes, such as random initialization, Latin hypercube sampling, Sobol sequences, the Kent map without opposition-based learning, and opposition-based learning without the Kent map, is left as an open question.

## 7. Conclusions and Future Work

This paper proposes MSCPO, a multi-strategy enhanced crested porcupine optimizer that addresses four limitations of the original CPO through four phase-targeted enhancement strategies: KCOL for population initialization, APO-DME for the first defense phase, ADDP for the second defense phase, and PDAP for the fourth defense phase. On the CEC 2017 suite, MSCPO attains the best overall mean rank (2.638), followed by LSHADE-SPACMA (3.112) and L-SHADE (4.371); it ranks first at D = 10, 30, and 50 (mean ranks 1.72, 2.41, and 2.76, respectively) but drops to third at D = 100. On the CEC 2022 suite, MSCPO ranks first at both tested dimensions (mean ranks 2.08 at D = 10 and 2.67 at D = 20; overall 2.375), followed overall by LSHADE-SPACMA (3.583) and L-SHADE (3.625), although at D = 20 the pairwise comparison against LSHADE-SPACMA records more losses than wins. The full ablation experiment over all 16 combinations of the four strategies shows that removing any single strategy degrades the overall ranking and that the complete MSCPO achieves the best mean rank (3.34), indicating that the four strategies contribute jointly to the final performance. On three constrained engineering design problems, MSCPO ranks first on the hydrostatic thrust bearing problem, second on the step-cone pulley problem, and third on the robotic gripper problem among eleven competing algorithms, confirming its applicability beyond benchmark functions.

Future work will proceed in three directions. First, the cyclic population reduction inherited from CPO follows a fixed schedule that does not account for the problem dimension; adapting the population schedule to the dimensionality or to the search progress, in the spirit of the linear population size reduction in L-SHADE [32] will be investigated to improve the performance of MSCPO on high-dimensional problems. Second, KCOL will be systematically compared with alternative initialization schemes, including Latin hypercube sampling and Sobol-based sequences, and the possibility of combining schemes, for instance by integrating Sobol-based sampling with opposition-based learning, or adaptively selecting them according to problem characteristics will be explored. Third, the phase-targeted framework will be extended to multi-objective optimization, as explored by multi-objective crested porcupines optimization (MOCPO) [49] and multi-objective crested porcupine optimization algorithm based on multiple defense strategies (MCPOMD) [50].

## Figures and Tables

**Table 1:** Correspondence between CPO Phases and MSCPO Strategies.

CPO Phase	Enhancement Strategy	Modification Type	Targeted Weakness
Initialization (Phase 0)	KCOL	Replacement	Uniform randomness causes clustering and poor boundary coverage
First Defense (Phase 1)	APO-DME	Replacement	Single-point dependency on $x_{CP}$ causes exploration stagnation
Second Defense (Phase 2)	ADDP	Replacement	Single differential vector limits search direction diversity
Third Defense (Phase 3)	Original preserved	Unchanged	—
Fourth Defense (Phase 4)	PDAP	Replacement	Fixed α imposes a constant exploration-exploitation balance throughout the search, and the update contains no periodic perturbation component

**Table 2:** Description of the CEC 2017 test suite [43].

Function Type	No.	Name	[*lb*, *ub*]	$f_{min}$
Unimodal Functions	F1	Shifted and Rotated Bent Cigar Function	[−100, 100]	100
	F3	Shifted and Rotated Zakharov Function	[−100, 100]	300
Multimodal Functions	F4	Shifted and Rotated Rosenbrock’s Function	[−100, 100]	400
	F5	Shifted and Rotated Rastrigin’s Function	[−100, 100]	500
	F6	Shifted and Rotated Expanded Scaffer’s F6 Function	[−100, 100]	600
	F7	Shifted and Rotated Lunacek Bi−Rastrigin Function	[−100, 100]	700
	F8	Shifted and Rotated Non-Continuous Rastrigin Function	[−100, 100]	800
	F9	Shifted and Rotated Levy Function	[−100, 100]	900
	F10	Shifted and Rotated Schwefel’s Function	[−100, 100]	1000
Hybrid Functions	F11	Hybrid Function 1 (*N* = 3)	[−100, 100]	1100
	F12	Hybrid Function 2 (*N* = 3)	[−100, 100]	1200
	F13	Hybrid Function 3 (*N* = 3)	[−100, 100]	1300
	F14	Hybrid Function 4 (*N* = 4)	[−100, 100]	1400
	F15	Hybrid Function 5 (*N* = 4)	[−100, 100]	1500
	F16	Hybrid Function 6 (*N* = 4)	[−100, 100]	1600
	F17	Hybrid Function 7 (*N* = 5)	[−100, 100]	1700
	F18	Hybrid Function 8 (*N* = 5)	[−100, 100]	1800
	F19	Hybrid Function 9 (*N* = 5)	[−100, 100]	1900
	F20	Hybrid Function 10 (*N* = 6)	[−100, 100]	2000
Composition Functions	F21	Composition Function 1 (*N* = 3)	[−100, 100]	2100
	F22	Composition Function 2 (*N* = 3)	[−100, 100]	2200
	F23	Composition Function 3 (*N* = 4)	[−100, 100]	2300
	F24	Composition Function 4 (*N* = 4)	[−100, 100]	2400
	F25	Composition Function 5 (*N* = 5)	[−100, 100]	2500
	F26	Composition Function 6 (*N* = 5)	[−100, 100]	2600
	F27	Composition Function 7 (*N* = 6)	[−100, 100]	2700
	F28	Composition Function 8 (*N* = 6)	[−100, 100]	2800
	F29	Composition Function 9 (N = 6)	[−100, 100]	2900
	F30	Composition Function 10 (*N* = 3)	[−100, 100]	3000

Note: *N* in the function names denotes the number of component functions of the hybrid/composition function, as defined in the official technical reports [43].

**Table 3:** Description of the CEC 2022 test suite [44].

Function Type	No.	Name	[*lb*, *ub*]	$f_{min}$
Unimodal Function	F1	Shifted and full rotated Zakharov function	[−100, 100]	300
Basic Functions	F2	Shifted and full rotated Rosenbrock’s function	[−100, 100]	400
	F3	Shifted and full rotated expanded Schaffer’s F6 function	[−100, 100]	600
	F4	Shifted and full rotated non-continuous Rastrigin’s function	[−100, 100]	800
	F5	Shifted and full rotated Levy function	[−100, 100]	900
Hybrid Functions	F6	Hybrid function 1 (*N* = 3)	[−100, 100]	1800
	F7	Hybrid function 2 (*N* = 6)	[−100, 100]	2000
	F8	Hybrid function 3 (*N* = 5)	[−100, 100]	2200
Composition Functions	F9	Composition function 1 (*N* = 5)	[−100, 100]	2300
	F10	Composition function 2 (*N* = 4)	[−100, 100]	2400
	F11	Composition function 3 (*N* = 5)	[−100, 100]	2600
	F12	Composition function 4 (*N* = 6)	[−100, 100]	2700

Note: *N* in the function names denotes the number of component functions of the hybrid/composition function, as defined in the official technical reports [44].

**Table 4:** Compositions of CPO, MSCPO, and the 14 intermediate variants.

Algorithms	KCOL	APO-DME	ADDP	PDAP
CPO	No	No	No	No
CPO_KCOL	Yes	No	No	No
CPO_APODME	No	Yes	No	No
CPO_ADDP	No	No	Yes	No
CPO_PDAP	No	No	No	Yes
CPO_KCOL_APODME	Yes	Yes	No	No
CPO_KCOL_ADDP	Yes	No	Yes	No
CPO_KCOL_PDAP	Yes	No	No	Yes
CPO_APODME_ADDP	No	Yes	Yes	No
CPO_APODME_PDAP	No	Yes	No	Yes
CPO_ADDP_PDAP	No	No	Yes	Yes
MSCPO_woKCOL	No	Yes	Yes	Yes
MSCPO_woAPODME	Yes	No	Yes	Yes
MSCPO_woADDP	Yes	Yes	No	Yes
MSCPO_woPDAP	Yes	Yes	Yes	No
MSCPO	Yes	Yes	Yes	Yes

**Table 5:** Ablation study results of the single-strategy tier on CEC 2017 at D = 30.

Algorithms	Mean Rank	No. Best	No. Worst
MSCPO	1.3793	22	0
CPO_ADDP	2.8621	1	2
CPO_APODME	2.9655	3	2
CPO_PDAP	4.1034	2	5
CPO	4.8276	1	9
CPO_KCOL	4.8621	0	11

Friedman test: $\chi_{F}^{2}$ = 76.00, *p* = 5.77 × 10^−15^ (<0.001), indicating significant performance differences among the six variants of the single-strategy tier. CD (Nemenyi, k=6, N=29, $q_{0.05}=2.850$) = 1.40.

**Table 6:** Full-factorial ablation of all strategy combinations on CEC 2017 at D = 30.

Tier	Variant	Mean Rank	No. Best	No. Worst	*p*-Value vs. MSCPO	Significant
Full	MSCPO	3.3448	8	0	-	-
Three-strategy	MSCPO_woPDAP	3.9655	8	1	6.81 × 10^−1^	No
Three-strategy	MSCPO_woKCOL	4.0690	6	0	1.41 × 10^−1^	No
Pairwise	CPO_APODME_ADDP	5.6552	1	0	1.48 × 10^−3^	Yes
Pairwise	CPO_APODME_PDAP	7.1034	2	0	6.44 × 10^−3^	Yes
Three-strategy	MSCPO_woAPODME	7.5517	0	0	3.00 × 10^−5^	Yes
Pairwise	CPO_ADDP_PDAP	7.7931	0	0	3.31 × 10^−4^	Yes
Three-strategy	MSCPO_woADDP	8.0690	2	0	1.27 × 10^−3^	Yes
Pairwise	CPO_KCOL_ADDP	8.4483	0	0	8.41 × 10^−6^	Yes
Single- strategy	CPO_ADDP	9.0345	0	1	8.31 × 10^−5^	Yes
Single- strategy	CPO_APODME	9.2069	0	0	3.60 × 10^−4^	Yes
Pairwise	CPO_KCOL_APODME	9.4138	0	1	1.09 × 10^−4^	Yes
Single- strategy	CPO_PDAP	12.2069	0	3	1.54 × 10^−4^	Yes
Pairwise	CPO_KCOL_PDAP	13.0345	1	3	1.86 × 10^−5^	Yes
Baseline	CPO	13.4828	1	9	1.14 × 10^−5^	Yes
Single- strategy	CPO_KCOL	13.6207	0	11	1.14 × 10^−5^	Yes

Friedman test: $\chi_{F}^{2}$ = 211.58, *p* = 9.32 × 10^−37^ (<0.001). CD (Nemenyi, k=16, N=29, $q_{0.05}=3.426)$ = 4.28. *p*-values: Wilcoxon signed-rank test against MSCPO.

**Table 7:** Wilcoxon signed-rank test results for different competitor algorithms (CEC 2017).

MSCPO vs.	D = 10			D = 30			D = 50			D = 100			Total 1		
	+	−	=	+	−	=	+	−	=	+	−	=	+	−	=
CPO	20	1	8	20	0	9	23	0	6	24	0	5	87	1	28
GA	29	0	0	29	0	0	29	0	0	29	0	0	116	0	0
DE	23	4	2	26	0	3	29	0	0	29	0	0	107	4	5
WOA	29	0	0	27	0	2	27	0	2	27	2	0	110	2	4
HHO	29	0	0	27	2	0	27	2	0	26	3	0	109	7	0
DBO	29	0	0	29	0	0	29	0	0	28	0	1	115	0	1
GJA	27	1	1	17	7	5	16	10	3	10	13	6	70	31	15
SSA	28	0	1	26	2	1	23	2	4	18	7	4	95	11	10
BBO	28	0	1	19	5	5	12	9	8	9	18	2	68	32	16
L-SHADE	19	3	7	23	4	2	19	4	6	12	9	8	73	20	23
LSHADE-SPACMA	14	5	10	12	9	8	9	8	12	9	13	7	44	35	37
Total 2	275	14	30	255	29	35	243	35	41	221	65	33	994	143	139

**Table 8:** Friedman mean rank test results for CEC 2017 (α=0.05).

Algorithm	D = 10	D = 30	D = 50	D = 100	Mean	Rank
MSCPO	**1.72**	**2.41**	**2.76**	3.66	**2.64**	**1**
CPO	3.03	4.14	5.14	5.66	4.49	5
GA	8.31	6.76	6.31	5.38	6.69	7
DE	9.72	8.69	8.24	7.86	8.63	9
WOA	10.69	10.97	11.59	11.79	11.26	12
HHO	4.97	7.14	7.86	8.45	7.10	8
DBO	9.38	10.55	10.72	10.59	10.31	10
GJA	6.38	4.69	3.86	**3.10**	4.51	6
SSA	5.76	4.66	4.03	3.48	4.48	4
BBO	11.00	10.72	10.17	9.72	10.41	11
L-SHADE	3.83	4.72	4.38	4.55	4.37	3
LSHADE-SPACMA	3.21	2.55	2.93	3.76	3.11	2
Friedman *p*-value	8.17 × 10^−49^	5.41 × 10^−45^	1.70 × 10^−45^	6.04 × 10^−43^	N/A	N/A

Bold values indicate the best result. N/A: not applicable (the Friedman *p*-value is computed per dimension and does not apply to the averaged Mean and Rank columns).

**Table 9:** Wilcoxon signed-rank test results for different competitor algorithms (CEC 2022).

MSCPO vs.	D = 10			D = 20			Total 1		
	+	−	=	+	−	=	+	−	=
CPO	6	2	4	7	0	5	13	2	9
GA	12	0	0	12	0	0	24	0	0
DE	7	3	2	9	2	1	16	5	3
WOA	12	0	0	12	0	0	24	0	0
HHO	11	0	1	12	0	0	23	0	1
DBO	11	0	1	12	0	0	23	0	1
GJA	11	0	1	9	1	2	20	1	3
SSA	11	0	1	11	0	1	22	0	2
BBO	10	1	1	8	1	3	18	2	4
L-SHADE	3	2	7	5	4	3	8	6	10
LSHADE-SPACMA	7	1	4	3	5	4	10	6	8
Total 2	101	9	22	100	13	19	201	22	41

**Table 10:** Friedman mean rank test results for CEC 2022 (α = 0.05).

Algorithm	D = 10	D = 20	Mean	Rank
MSCPO	**2.08**	**2.67**	**2.38**	**1**
CPO	3.00	4.42	3.71	4
GA	8.17	7.33	7.75	8
DE	8.75	9.00	8.88	9
WOA	11.25	11.08	11.17	12
HHO	5.00	5.17	5.08	5
DBO	9.00	10.17	9.58	10
GJA	5.58	5.42	5.50	6
SSA	6.58	6.17	6.38	7
BBO	10.50	10.25	10.38	11
L-SHADE	3.75	3.50	3.63	3
LSHADE-SPACMA	4.33	2.83	3.58	2
Friedman *p*-value	6.94 × 10^−15^	5.88 × 10^−15^	N/A	N/A

Bold values indicate the best result. N/A: not applicable (the Friedman *p*-value is computed per dimension and does not apply to the averaged Mean and Rank columns).

**Table 11:** Best solutions for the step-cone pulley design problem.

Algorithm	Optimal Values for Variables					Optimal Cost
	$d_{1}$	$d_{2}$	$d_{3}$	$d_{4}$	w	
MSCPO	38.68	53.23	70.97	85.09	89.64	16.16
CPO	39.99	55.03	73.37	87.96	86.97	18.41
GA	40.11	55.20	73.59	88.24	87.50	44.32
DE	40.92	56.30	75.07	90.00	84.49	2.10 × 103
WOA	39.73	54.67	72.88	87.38	87.38	16.89
HHO	39.67	54.58	72.77	87.25	87.25	16.63
DBO	40.91	56.31	75.05	90.00	90.00	2.70 × 106
GJA	39.99	55.03	73.36	87.96	90.00	3.27 × 105
SSA	38.63	53.16	70.88	84.98	89.49	16.18
BBO	38.56	53.06	70.75	84.82	89.67	16.26
L-SHADE	38.74	53.30	71.06	85.20	89.32	16.26
LSHADE-SPACMA	38.41	52.86	70.47	84.50	90.00	**16.09**

Bold values indicate the best result.

**Table 12:** Statistical results for the step-cone pulley design problem.

Algorithm	Min	Worst	Std	Mean	Median
MSCPO	16.16	16.68	8.89 × 10^−2^	16.47	16.49
CPO	18.41	1.70 × 10^2^	3.20 × 10^1^	5.63 × 10^1^	5.08 × 10^1^
GA	44.32	9.46 × 10^8^	1.88 × 10^8^	6.26 × 10^7^	1.48 × 10^5^
DE	2.10 × 10^3^	4.35 × 10^6^	8.19 × 10^5^	4.23 × 10^5^	1.56 × 10^5^
WOA	16.89	2.23 × 10^11^	4.09 × 10^10^	9.39 × 10^9^	26.78
HHO	16.63	2.11 × 10^7^	4.04 × 10^6^	9.55 × 10^5^	17.77
DBO	2.70 × 10^6^	7.57 × 10^9^	2.06 × 10^9^	1.11 × 10^9^	7.80 × 10^7^
GJA	3.27 × 10^5^	8.03 × 10^6^	2.00 × 10^6^	2.69 × 10^6^	2.54 × 10^6^
SSA	16.18	16.89	2.02 × 10^−1^	16.58	16.60
BBO	16.26	17.51	3.37 × 10^−1^	16.82	16.83
L-SHADE	16.26	16.92	1.69 × 10^−1^	16.62	16.64
LSHADE-SPACMA	**16.09**	**16.09**	**2.40 × 10^−7^**	**16.09**	**16.09**

Bold values indicate the best result.

**Table 13:** Best solutions for the hydrostatic thrust bearing design problem.

Algorithm	Optimal Values for Variables				Optimal Cost
	R	$R_{0}$	μ	Q	
MSCPO	5.97	5.40	5.40 × 10^−6^	2.31	**1632.34**
CPO	6.17	5.60	6.63 × 10^−6^	3.76	1879.01
GA	7.75	7.01	5.73 × 10^−6^	4.97	2988.75
DE	6.87	6.10	5.88 × 10^−6^	4.11	2454.06
WOA	6.07	5.50	5.57 × 10^−6^	2.51	1687.66
HHO	7.43	6.98	7.50 × 10^−6^	6.91	2385.81
DBO	6.74	6.13	5.97 × 10^−6^	3.81	2265.77
GJA	6.20	5.65	7.43 × 10^−6^	5.04	1961.74
SSA	6.09	5.48	6.92 × 10^−6^	4.18	1926.34
BBO	6.05	5.49	5.69 × 10^−6^	2.59	1684.29
L-SHADE	5.97	5.39	5.44 × 10^−6^	2.34	1637.26
LSHADE-SPACMA	5.96	5.39	5.51 × 10^−6^	2.37	1633.25

Bold values indicate the best result.

**Table 14:** Statistical results for the hydrostatic thrust bearing design problem.

Algorithm	Min	Worst	Std	Mean	Median
MSCPO	**1632.34**	**1839.35**	**61.02**	**1730.27**	**1728.63**
CPO	1879.01	2308.01	103.81	2049.73	2039.92
GA	2988.75	4.12 × 10^18^	7.53 × 10^17^	1.38 × 10^17^	6175.14
DE	2454.06	3494.79	287.19	2943.60	2866.63
WOA	1687.66	1.06 × 10^9^	2.69 × 10^8^	7.08 × 10^7^	2685.88
HHO	2385.81	1.20 × 10^9^	3.56 × 10^8^	1.17 × 10^8^	3414.52
DBO	2265.77	4558.33	558.88	3158.51	3120.30
GJA	1961.74	2943.01	216.57	2341.68	2350.12
SSA	1926.34	6139.42	976.11	3124.16	2907.73
BBO	1684.29	2914.24	345.60	2134.71	1994.14
L-SHADE	1637.26	1897.13	61.72	1774.11	1782.86
LSHADE-SPACMA	1633.25	2042.79	105.84	1822.31	1810.40

Bold values indicate the best result.

**Table 15:** Best solutions obtained for the robotic gripper design problem.

Algorithm	Optimal Values for Variables							Optimal Cost
	a	b	c	e	f	l	δ	
MSCPO	149.24	141.19	195.60	7.84	144.60	107.41	2.44	2.7312
CPO	148.03	139.59	186.22	7.89	107.75	121.30	2.24	3.2382
GA	143.73	124.97	167.63	16.57	94.03	142.80	2.39	4.2925
DE	150.00	149.73	197.72	0.00	10.00	114.75	1.64	3.0227
WOA	150.00	149.85	178.98	0.00	150.00	103.75	2.38	2.8980
HHO	150.00	149.88	176.97	0.00	150.00	100.85	2.45	2.8730
DBO	146.89	146.83	193.93	0.00	10.00	101.59	1.58	3.2309
GJA	150.00	131.13	189.05	18.69	128.12	103.96	2.37	2.9083
SSA	144.88	144.74	173.44	0.00	10.00	101.52	1.54	3.0588
BBO	149.93	137.44	199.21	12.30	100.28	105.46	2.15	2.7965
L-SHADE	149.40	145.27	199.97	3.98	147.43	103.38	2.35	2.6502
LSHADE-SPACMA	150.00	149.88	200.00	0.00	150.00	100.94	2.30	**2.5438**

Bold values indicate the best result.

**Table 16:** Statistical results for the robotic gripper design problem.

Algorithm	Min	Worst	Std	Mean	Median
MSCPO	2.7312	3.3110	0.1277	3.0344	3.0357
CPO	3.2382	4.3134	0.2389	3.9336	3.9156
GA	4.2925	36.4711	9.2051	10.7331	7.0667
DE	3.0227	4.7173	0.4187	3.9113	3.8639
WOA	2.8980	8.4718	1.3979	5.6896	5.5061
HHO	2.8730	66.2347	11.1674	7.4029	5.2083
DBO	3.2309	5.7523	0.6130	4.2780	4.2784
GJA	2.9083	4.4986	0.2920	3.2644	3.2086
SSA	3.0588	7.2062	1.2138	4.4945	4.1173
BBO	2.7965	4.4330	0.3860	3.1662	3.1063
L-SHADE	2.6502	3.1374	0.1245	2.7846	2.7439
LSHADE-SPACMA	**2.5438**	**2.5466**	**5.81 × 10^−4^**	**2.5439**	**2.5438**

Bold values indicate the best result.

**Table 17:** Average wall-clock runtime per run of CPO and MSCPO (in seconds). Each value is the mean over 30 independent runs, averaged over all 29 (CEC 2017) or 12 (CEC 2022) functions at the corresponding dimension. All timings were measured within a single MATLAB R2023b process with plotting disabled.

Benchmark	Configuration	CPO (s)	MSCPO (s)	Ratio (MSCPO/CPO)
CEC 2017	D = 10	0.0466	0.0671	1.44
CEC 2017	D = 30	0.0820	0.1027	1.25
CEC 2017	D = 50	0.1295	0.1553	1.20
CEC 2017	D = 100	0.2949	0.3327	1.13
CEC 2022	D = 10	0.0487	0.0698	1.43
CEC 2022	D = 20	0.0679	0.0875	1.29
Step-cone pulley	D = 5	0.7471	0.8804	1.18
Hydrostatic thrust bearing	D = 4	0.6144	0.7363	1.20
Robotic gripper	D = 7	31.9769	32.9921	1.03

**Table 18:** Comparison of prior variants and MSCPO.

Algorithm	Initialization	Enhancement Strategies	Validation Testbeds
ECPO [18]	Sobol	guided search, adaptive Levy flight, centroid-based OBL	CEC 2014 (D = 30), CEC 2017 (D = 30), CEC 2020, CEC 2022
BTCPO [36]	random	butterfly search, triangular walk	23 classical functions, CEC 2021
CAPCPO [38]	random	composite Cauchy mutation, adaptive dynamic adjustment, population mutation	CEC 2017 (D = 30; scalability at D = 100), CEC 2019, CEC 2022 (D = 10, 20)
SDHCPO [48]	Sobol-OBL	cosine annealing, DE/rand/1, horizontal-vertical crossover	CEC 2017 (D = 30, 50), CEC 2022 (D = 20)
MSCPO	KCOL	APO-DME (Phase 1), ADDP (Phase 2), PDAP (Phase 4)	CEC 2017 (D = 10, 30, 50, 100), CEC 2022 (D = 10, 20)

## Data Availability

The data presented in this study, including the MSCPO source code, the parameter settings of all compared algorithms, the raw outputs of all independent runs, and the random seeds used, are available from the corresponding author upon reasonable request.

## Abbreviations

The following abbreviations are used in this manuscript:

CPO	Crested Porcupine Optimizer
MSCPO	Multi-Strategy Enhanced Crested Porcupine Optimizer
KCOL	Kent Chaos Opposition-Based Learning
APO-DME	Arctic Puffin Optimization (APO)-Inspired Dual-Modal Evasion
ADDP	Adaptive Dual-Differential Perturbation
PDAP	Periodic Dynamic Adaptive Perturbation
GA	genetic algorithm
GWO	Gray Wolf Optimizer
WOA	Whale optimization algorithm
DE	differential evolution
GBO	gradient-based optimizer
AVOA	African vultures optimization algorithm
RUN	Runge–Kutta method
EO	equilibrium optimizer
GTO	gorilla troops optimizer
UAV	unmanned aerial vehicle
ECPO	Enhanced CPO
BTCPO	Butterfly Search and Triangular Walk CPO
CAPCPO	Composite Adaptive CPO
HHO	Harris Hawks Optimization
DBO	dung beetle optimizer
GJA	Gekko Japonicus Algorithm
SSA	Sparrow search algorithm
BBO	Beaver behavior optimizer
L-SHADE	success-history based adaptive differential evolution with linear population size reduction
LSHADE-SPACMA	LSHADE with semi-parameter adaptation hybrid with CMA-ES
CP	crested porcupines
OBL	opposition-based learning
APO	Arctic Puffin Optimization
CD	Critical Difference
MOCPO	multi-objective crested porcupines optimization
MCPOMD	multi-objective crested porcupine optimization algorithm based on multiple defense strategies

## Appendix A

**Table A1:** Parameter settings of MSCPO and all compared algorithms, as used in the experiments.

Algorithm	Parameter Settings
GA	$\mathrm{Real}\text{-}\mathrm{coded}\;\mathrm{GA};\;\mathrm{roulette}\text{-}\mathrm{wheel}\;\mathrm{selection};\;\mathrm{arithmetic}\;\mathrm{crossover}\;\mathrm{with}\;\mathrm{probability}\;p_{c}=0.6;$ $\mathrm{non}\text{-}\mathrm{uniform}\;\mathrm{mutation}\;\mathrm{with}\;\mathrm{probability}\;p_{m}=0.3;$ infeasible offspring regenerated until within the bounds
DE	DE/rand/1/bin; scale factor β~U(0.3, 0.9) $(\mathrm{resampled}\;\mathrm{per}\;\mathrm{dimension});\;\mathrm{crossover}\;\mathrm{probability}\;p_{CR}=0.2$
WOA	Logarithmic spiral constant b=1; coefficient a $\mathrm{decreases}\;\mathrm{linearly}\;\mathrm{from}\;2\;\mathrm{to}\;0;\;a_{2}$ decreases linearly from−1 to−2
HHO	$\mathrm{Initial}\;\mathrm{energy}\;E_{0}\sim U(-1,\;1);$ $\mathrm{escaping}\;\mathrm{energy}\;E_{1}=2(1-t/T_{max});$ Levy exponent β=1.5
DBO	Population proportions: ball-rolling beetles 20%, brood balls 25%, small dung beetles 30%, thief beetles 25%; k=0.1, b=0.2; $\mathrm{dancing}\;\mathrm{probability}\;0.2;\;P\left(\alpha =-1\right)=0.1;$ $R=1-t/T_{max}$
GJA	Step-size coefficient β decays from 1.2 to 0.3 via the cosh schedule; α=0.6β; guidance factor 0.3 applied every 3 generations; memory updated every 5 generations (capacity 5, replacing the worst 10%)
SSA	$\mathrm{Producer}\;\mathrm{ratio}\;P_{D}=0.2;$ $\mathrm{scrounger}\;\mathrm{ratio}\;S_{D}=0.3;$ safety threshold ST=0.8
BBO	$\mathrm{Exploitation}\;\mathrm{probability}\;E\left(t\right)=sin(\pi t/{2T}_{max})$ (exploitation if rand<E(t), $\mathrm{exploration}\;\mathrm{otherwise});\;\mathrm{architect}\;\mathrm{proportion}\;25\%;\;\mathrm{per}\text{-}\mathrm{dimension}\;\mathrm{perturbation}\;\mathrm{applied}\;\mathrm{with}\;\mathrm{probability}\;0.5:\;(1/10)\cos(\pi t/{2T}_{max}$ $)(u_{j}-l_{j})N(0,1)$
L-SHADE	Memory size H=N; $\mathrm{initial}\;\mathrm{memory}\;M_{F}=M_{CR}=0.5;$ $F\sim Cauchy\left(M_{F},\;0.1\right)$ truncated to [0, 1]; $CR\sim N(M_{CR},0.1)$ truncated to [0, 1]$;\;p\sim U(\frac{2}{N},0.2);$ external archive of size at most N; $\mathrm{linear}\;\mathrm{population}\;\mathrm{size}\;\mathrm{reduction}\;\mathrm{to}\;N_{min}=4;$ $\mathrm{evaluation}\;\mathrm{budget}\;FES=\;N\cdot T_{max}$
LSHADE-SPACMA	$p_{best}=0.11;$ memory size H=5; $\mathrm{archive}\;\mathrm{rate}\;1.4;\;N_{min}=4;$ first half of the run F=0.45+0.1U(0,1), $\mathrm{second}\;\mathrm{half}\;F\sim Cauchy\left(\mu_{F},\;0.1\right);$ CMA-ES crossover applied with probability 0.5; σ=0.5
CPO	Cyclic parameter T=2; convergence acceleration factor α=0.1; $\mathrm{third}/\mathrm{fourth}\;\mathrm{defense}\;\mathrm{trade}\text{-}\mathrm{off}\;\mathrm{probability}\;T_{f}=0.8;$ $\mathrm{population}\;\mathrm{size}\;\mathrm{reduction}\;\mathrm{to}\;N_{min}$ as in Equation (2)
MSCPO	CPO parameters unchanged (T=2, $T_{f}=0.8,$ $N_{min}$); the fixed α is not used because PDAP replaces the fourth defense. KCOL: Kent map control parameter r=0.4, $\mathrm{chaotic}\;\mathrm{iteration}\;\mathrm{count}\;M_{c}=10.$ APO-DME: mode 1 coefficient ε~U(0,1); $\mathrm{mode}\;2\;\mathrm{scaling}\;\mathrm{factor}\;F_{L}=0.5$ with Levy exponent β=1.5. ADDP: scaling factor F~U(0,1) $\left(\mathrm{resampled}\;\mathrm{per}\;\mathrm{update}).\;\mathrm{PDAP}:\;\eta_{t}=\sin\left(2\pi U_{1}\right)+cos(2\pi U_{2}\right)$ $\mathrm{with}\;U_{1},\;U_{2}\sim U(0,1);$ $I\in \left\{1,2\right\}$ with equal probability

Shared settings: benchmark experiments (CEC 2017/CEC 2022) used N=30 and $T_{max}=1000$; engineering problems used N=100 and $T_{max}=500$. All algorithms were executed over 30 independent runs. Note that MSCPO inherits all parameters of the original CPO; the coefficients of its four strategies are either fixed constants or random variables drawn from fixed distributions at each update, so no problem-specific tuning is required.

**Table A2:** Error statistics of different algorithms on CEC2017 (D = 10).

ID	Metric	MSCPO	CPO	GA	DE	WOA	HHO	DBO	GJA	SSA	BBO	LSHADE	LSHADE-SPACMA
CEC2017-F1	Avg	**1.0000 × 10^2^**	1.0000 × 10^2^	2.1308 × 10^5^	3.4555 × 10^3^	9.2736 × 10^6^	5.0983 × 10^5^	1.5744 × 10^9^	6.0335 × 10^4^	4.0127 × 10^3^	1.1562 × 10^3^	1.0000 × 10^2^	1.0000 × 10^2^
	Std	2.6305 × 10^−9^	9.0566 × 10^−3^	3.1720 × 10^5^	2.8018 × 10^3^	1.5927 × 10^7^	2.3499 × 10^5^	2.2465 × 10^9^	1.4527 × 10^4^	3.9010 × 10^3^	1.7576 × 10^3^	7.9670 × 10^−13^	**3.7320 × 10^−15^**
CEC2017-F3	Avg	**3.0000 × 10^2^**	3.0000 × 10^2^	5.2183 × 10^4^	3.6152 × 10^3^	3.6674 × 10^3^	3.0800 × 10^2^	2.7156 × 10^3^	3.0017 × 10^2^	3.0000 × 10^2^	3.0000 × 10^2^	**3.0000 × 10^2^**	1.4328 × 10^3^
	Std	1.0556 × 10^−14^	9.0865 × 10^−5^	2.5456 × 10^4^	1.2624 × 10^3^	3.4369 × 10^3^	8.8752 × 10^0^	1.1304 × 10^3^	4.9814 × 10^−2^	1.4615 × 10^−6^	5.9241 × 10^−4^	**0.0000 × 10^0^**	4.3436 × 10^3^
CEC2017-F4	Avg	4.0000 × 10^2^	4.0157 × 10^2^	4.3971 × 10^2^	4.0617 × 10^2^	4.2778 × 10^2^	4.2255 × 10^2^	4.9108 × 10^2^	4.0477 × 10^2^	4.0777 × 10^2^	4.0485 × 10^2^	4.0037 × 10^2^	**4.0000 × 10^2^**
	Std	6.2837 × 10^−4^	9.6684 × 10^−1^	3.9871 × 10^1^	4.0610 × 10^−1^	4.2599 × 10^1^	2.9419 × 10^1^	4.5908 × 10^1^	8.0927 × 10^−1^	1.6778 × 10^1^	1.2298 × 10^0^	1.8859 × 10^−1^	**2.1111 × 10^−14^**
CEC2017-F5	Avg	5.0492 × 10^2^	5.0758 × 10^2^	5.8689 × 10^2^	5.1296 × 10^2^	5.5567 × 10^2^	5.5119 × 10^2^	5.4932 × 10^2^	5.1142 × 10^2^	5.3914 × 10^2^	5.1254 × 10^2^	5.1111 × 10^2^	**5.0455 × 10^2^**
	Std	1.8710 × 10^0^	2.9017 × 10^0^	1.7178 × 10^1^	2.4673 × 10^0^	2.2751 × 10^1^	2.1813 × 10^1^	7.4406 × 10^0^	5.5593 × 10^0^	1.4716 × 10^1^	3.9506 × 10^0^	2.2347 × 10^0^	**1.2788 × 10^0^**
CEC2017-F6	Avg	**6.0000 × 10^2^**	6.0000 × 10^2^	6.6079 × 10^2^	**6.0000 × 10^2^**	6.3699 × 10^2^	6.3676 × 10^2^	6.2263 × 10^2^	6.0029 × 10^2^	6.1416 × 10^2^	6.0017 × 10^2^	**6.0000 × 10^2^**	6.0000 × 10^2^
	Std	9.8260 × 10^−11^	2.1640 × 10^−7^	1.0839 × 10^1^	1.0556 × 10^−13^	1.3114 × 10^1^	1.3205 × 10^1^	5.1227 × 10^0^	2.1279 × 10^−1^	1.3309 × 10^1^	4.8955 × 10^−1^	**5.1711 × 10^−14^**	1.9807 × 10^−3^
CEC2017-F7	Avg	7.1623 × 10^2^	7.2098 × 10^2^	8.0464 × 10^2^	7.2485 × 10^2^	7.8804 × 10^2^	7.8425 × 10^2^	7.7835 × 10^2^	7.1974 × 10^2^	7.9206 × 10^2^	7.2442 × 10^2^	7.2267 × 10^2^	**7.1401 × 10^2^**
	Std	2.7612 × 10^0^	3.1904 × 10^0^	1.9932 × 10^1^	3.0003 × 10^0^	2.5857 × 10^1^	2.3157 × 10^1^	8.9945 × 10^0^	3.8948 × 10^0^	1.8073 × 10^1^	7.7348 × 10^0^	2.7500 × 10^0^	**1.8039 × 10^0^**
CEC2017-F8	Avg	8.0510 × 10^2^	8.0788 × 10^2^	8.7985 × 10^2^	8.1252 × 10^2^	8.4441 × 10^2^	8.3044 × 10^2^	8.3302 × 10^2^	8.0857 × 10^2^	8.3688 × 10^2^	8.1380 × 10^2^	8.1009 × 10^2^	**8.0454 × 10^2^**
	Std	2.4024 × 10^0^	3.1356 × 10^0^	1.4035 × 10^1^	2.6089 × 10^0^	1.5919 × 10^1^	7.5385 × 10^0^	4.1537 × 10^0^	3.0209 × 10^0^	1.2646 × 10^1^	5.5780 × 10^0^	2.1546 × 10^0^	**1.4406 × 10^0^**
CEC2017-F9	Avg	**9.0000 × 10^2^**	**9.0000 × 10^2^**	9.4062 × 10^2^	**9.0000 × 10^2^**	1.5312 × 10^3^	1.4822 × 10^3^	1.0510 × 10^3^	9.0003 × 10^2^	1.4986 × 10^3^	9.0000 × 10^2^	**9.0000 × 10^2^**	9.0019 × 10^2^
	Std	**0.0000 × 10^0^**	**0.0000 × 10^0^**	2.1103 × 10^1^	2.1111 × 10^−14^	3.5566 × 10^2^	2.2157 × 10^2^	8.5046 × 10^1^	1.3192 × 10^−2^	3.2293 × 10^2^	1.6334 × 10^−2^	**0.0000 × 10^0^**	4.2131 × 10^−1^
CEC2017-F10	Avg	1.3850 × 10^3^	1.4546 × 10^3^	2.3310 × 10^3^	1.5585 × 10^3^	2.1530 × 10^3^	1.9964 × 10^3^	2.1067 × 10^3^	1.4579 × 10^3^	1.9900 × 10^3^	1.6029 × 10^3^	1.7002 × 10^3^	**1.2336 × 10^3^**
	Std	2.5144 × 10^2^	2.0363 × 10^2^	2.6950 × 10^2^	1.2370 × 10^2^	2.5964 × 10^2^	2.8264 × 10^2^	2.6932 × 10^2^	2.7681 × 10^2^	3.1423 × 10^2^	1.9246 × 10^2^	1.5030 × 10^2^	**9.6545 × 10^1^**
CEC2017-F11	Avg	**1.1012 × 10^3^**	1.1034 × 10^3^	1.1661 × 10^4^	1.1326 × 10^3^	1.4972 × 10^3^	1.1680 × 10^3^	1.3126 × 10^3^	1.1130 × 10^3^	1.1511 × 10^3^	1.1242 × 10^3^	1.1022 × 10^3^	1.1110 × 10^3^
	Std	**1.0726 × 10^0^**	1.8493 × 10^0^	1.0270 × 10^4^	1.4602 × 10^1^	4.0645 × 10^2^	3.3476 × 10^1^	9.6112 × 10^1^	5.2848 × 10^0^	4.5365 × 10^1^	1.1194 × 10^1^	1.2028 × 10^0^	1.1612 × 10^1^
CEC2017-F12	Avg	**1.3398 × 10^3^**	2.2789 × 10^3^	6.4924 × 10^6^	4.0698 × 10^5^	5.6736 × 10^6^	3.8132 × 10^6^	4.3865 × 10^6^	1.2424 × 10^5^	1.5677 × 10^4^	8.4582 × 10^4^	1.9810 × 10^3^	1.6611 × 10^3^
	Std	**9.5234 × 10^1^**	1.1545 × 10^3^	6.6704 × 10^6^	3.0597 × 10^5^	6.6411 × 10^6^	3.7614 × 10^6^	4.2195 × 10^6^	1.4522 × 10^5^	1.5374 × 10^4^	1.1472 × 10^5^	3.2408 × 10^2^	2.4245 × 10^2^
CEC2017-F13	Avg	**1.3046 × 10^3^**	1.3074 × 10^3^	1.8823 × 10^4^	3.5550 × 10^3^	2.1970 × 10^4^	1.7603 × 10^4^	2.0142 × 10^4^	8.1911 × 10^3^	1.0800 × 10^4^	9.0424 × 10^3^	1.3073 × 10^3^	1.3308 × 10^3^
	Std	**3.2287 × 10^0^**	3.3434 × 10^0^	1.0361 × 10^4^	2.8160 × 10^3^	1.6656 × 10^4^	1.2693 × 10^4^	1.2149 × 10^4^	3.8461 × 10^3^	9.4478 × 10^3^	6.3674 × 10^3^	3.3708 × 10^0^	2.6729 × 10^1^
CEC2017-F14	Avg	**1.4008 × 10^3^**	1.4016 × 10^3^	8.0191 × 10^3^	1.4472 × 10^3^	2.5428 × 10^3^	1.7346 × 10^3^	1.8851 × 10^3^	1.4758 × 10^3^	1.7154 × 10^3^	1.5682 × 10^3^	1.4094 × 10^3^	1.4131 × 10^3^
	Std	**6.8945 × 10^−1^**	1.4153 × 10^0^	7.3787 × 10^3^	3.7511 × 10^1^	1.4981 × 10^3^	3.8604 × 10^2^	3.9535 × 10^2^	4.1839 × 10^1^	4.0959 × 10^2^	2.9962 × 10^2^	9.5679 × 10^0^	1.0221 × 10^1^
CEC2017-F15	Avg	**1.5004 × 10^3^**	1.5007 × 10^3^	7.5832 × 10^3^	1.6738 × 10^3^	7.7883 × 10^3^	5.5997 × 10^3^	3.9640 × 10^3^	1.7886 × 10^3^	2.1196 × 10^3^	1.9157 × 10^3^	1.5006 × 10^3^	1.5072 × 10^3^
	Std	**4.8040 × 10^−1^**	4.8307 × 10^−1^	6.4733 × 10^3^	3.4147 × 10^2^	6.0100 × 10^3^	2.1356 × 10^3^	2.1938 × 10^3^	3.8686 × 10^2^	7.2705 × 10^2^	7.9715 × 10^2^	5.1399 × 10^−1^	7.2002 × 10^0^
CEC2017-F16	Avg	**1.6012 × 10^3^**	1.6129 × 10^3^	1.8443 × 10^3^	1.6069 × 10^3^	1.9264 × 10^3^	1.9629 × 10^3^	1.8023 × 10^3^	1.8014 × 10^3^	1.7974 × 10^3^	1.6940 × 10^3^	1.6049 × 10^3^	1.6057 × 10^3^
	Std	**2.0480 × 10^0^**	3.6229 × 10^1^	1.0531 × 10^2^	8.2450 × 10^0^	1.8573 × 10^2^	1.4890 × 10^2^	1.1681 × 10^2^	1.3563 × 10^2^	1.6427 × 10^2^	1.2854 × 10^2^	9.8076 × 10^0^	2.1686 × 10^1^
CEC2017-F17	Avg	1.7056 × 10^3^	1.7049 × 10^3^	1.7709 × 10^3^	1.7038 × 10^3^	1.8131 × 10^3^	1.7736 × 10^3^	1.7766 × 10^3^	1.7481 × 10^3^	1.7834 × 10^3^	1.7577 × 10^3^	1.7125 × 10^3^	**1.7021 × 10^3^**
	Std	3.4154 × 10^0^	3.6594 × 10^0^	2.3723 × 10^1^	4.6646 × 10^0^	5.5035 × 10^1^	5.4902 × 10^1^	1.4530 × 10^1^	3.2998 × 10^1^	5.7536 × 10^1^	2.8110 × 10^1^	9.0409 × 10^0^	**1.9638 × 10^0^**
CEC2017-F18	Avg	**1.8005 × 10^3^**	1.8017 × 10^3^	1.8240 × 10^4^	4.3509 × 10^3^	1.5720 × 10^4^	1.7930 × 10^4^	6.4543 × 10^4^	1.1102 × 10^4^	8.8492 × 10^3^	1.3046 × 10^4^	1.8140 × 10^3^	1.8345 × 10^3^
	Std	**2.9524 × 10^−1^**	9.5977 × 10^−1^	1.3783 × 10^4^	2.3513 × 10^3^	1.3620 × 10^4^	1.2196 × 10^4^	6.5817 × 10^4^	9.0020 × 10^3^	6.2154 × 10^3^	8.7685 × 10^3^	9.7070 × 10^0^	2.0980 × 10^1^
CEC2017-F19	Avg	**1.9002 × 10^3^**	1.9003 × 10^3^	9.1459 × 10^3^	2.1530 × 10^3^	1.3178 × 10^5^	1.1846 × 10^4^	6.6910 × 10^3^	2.1745 × 10^3^	3.8883 × 10^3^	2.5605 × 10^3^	1.9006 × 10^3^	1.9040 × 10^3^
	Std	**1.4234 × 10^−1^**	2.4416 × 10^−1^	8.7179 × 10^3^	5.5099 × 10^2^	2.3840 × 10^5^	1.0381 × 10^4^	1.3339 × 10^4^	3.8935 × 10^2^	2.3600 × 10^3^	1.0860 × 10^3^	6.7959 × 10^−1^	4.8929 × 10^0^
CEC2017-F20	Avg	**2.0000 × 10^3^**	2.0001 × 10^3^	2.1961 × 10^3^	2.0001 × 10^3^	2.1836 × 10^3^	2.1800 × 10^3^	2.1063 × 10^3^	2.0442 × 10^3^	2.0835 × 10^3^	2.0487 × 10^3^	2.0034 × 10^3^	2.0033 × 10^3^
	Std	**1.0789 × 10^−1^**	1.4964 × 10^−1^	5.9824 × 10^1^	2.0840 × 10^−1^	8.3843 × 10^1^	6.2062 × 10^1^	4.4730 × 10^1^	4.4847 × 10^1^	6.1836 × 10^1^	5.3680 × 10^1^	7.5588 × 10^0^	6.7861 × 10^0^
CEC2017-F21	Avg	**2.2000 × 10^3^**	2.2064 × 10^3^	2.2873 × 10^3^	2.2232 × 10^3^	2.3946 × 10^3^	2.4609 × 10^3^	2.2651 × 10^3^	2.2436 × 10^3^	2.3784 × 10^3^	2.2871 × 10^3^	2.2218 × 10^3^	2.2069 × 10^3^
	Std	**8.4444 × 10^−14^**	3.2200 × 10^1^	1.2853 × 10^2^	6.1531 × 10^0^	2.0993 × 10^2^	2.8833 × 10^2^	2.4040 × 10^1^	8.1225 × 10^1^	2.4786 × 10^2^	1.1959 × 10^2^	6.7193 × 10^1^	2.8349 × 10^1^
CEC2017-F22	Avg	**2.3289 × 10^3^**	2.5283 × 10^3^	5.0623 × 10^3^	3.5143 × 10^3^	5.2782 × 10^3^	5.6607 × 10^3^	3.6703 × 10^3^	3.7213 × 10^3^	5.1424 × 10^3^	3.8084 × 10^3^	3.5301 × 10^3^	2.7288 × 10^3^
	Std	**2.5906 × 10^2^**	6.3242 × 10^2^	1.1066 × 10^3^	6.7974 × 10^2^	1.2727 × 10^3^	1.1409 × 10^3^	1.1375 × 10^3^	8.4619 × 10^2^	1.0351 × 10^3^	6.7608 × 10^2^	6.5362 × 10^2^	3.7967 × 10^2^
CEC2017-F23	Avg	**2.6567 × 10^3^**	2.6807 × 10^3^	3.1283 × 10^3^	2.7440 × 10^3^	3.1158 × 10^3^	3.2837 × 10^3^	3.1238 × 10^3^	2.7359 × 10^3^	3.0372 × 10^3^	2.7463 × 10^3^	2.7038 × 10^3^	2.6612 × 10^3^
	Std	2.6033 × 10^1^	3.1948 × 10^1^	9.6542 × 10^1^	3.7153 × 10^1^	1.9744 × 10^2^	2.6116 × 10^2^	1.3478 × 10^2^	4.6001 × 10^1^	2.0740 × 10^2^	5.7889 × 10^1^	3.1076 × 10^1^	**2.3437 × 10^1^**
CEC2017-F24	Avg	**2.5367 × 10^3^**	2.5367 × 10^3^	2.8750 × 10^3^	2.5989 × 10^3^	3.0154 × 10^3^	2.6805 × 10^3^	2.7276 × 10^3^	2.5716 × 10^3^	2.6628 × 10^3^	2.6079 × 10^3^	2.5616 × 10^3^	2.5435 × 10^3^
	Std	4.9013 × 10^1^	4.9013 × 10^1^	2.6963 × 10^2^	**3.9072 × 10^1^**	4.0586 × 10^2^	3.0497 × 10^2^	6.3118 × 10^1^	7.1280 × 10^1^	3.5368 × 10^2^	1.5273 × 10^2^	9.6020 × 10^1^	5.0629 × 10^1^
CEC2017-F25	Avg	2.9098 × 10^3^	2.9158 × 10^3^	2.9923 × 10^3^	**2.9005 × 10^3^**	2.9431 × 10^3^	2.9178 × 10^3^	3.0125 × 10^3^	2.9180 × 10^3^	2.9215 × 10^3^	2.9220 × 10^3^	2.9072 × 10^3^	2.9059 × 10^3^
	Std	2.1120 × 10^1^	1.7746 × 10^1^	2.3541 × 10^1^	1.6858 × 10^1^	3.1678 × 10^1^	1.8121 × 10^1^	5.2459 × 10^1^	1.7066 × 10^1^	2.5755 × 10^1^	**1.5067 × 10^1^**	2.2919 × 10^1^	2.2140 × 10^1^
CEC2017-F26	Avg	2.9000 × 10^3^	2.9016 × 10^3^	3.7964 × 10^3^	2.9395 × 10^3^	3.6363 × 10^3^	3.4871 × 10^3^	3.1896 × 10^3^	2.8878 × 10^3^	3.5596 × 10^3^	**2.8687 × 10^3^**	2.9242 × 10^3^	2.9160 × 10^3^
	Std	**0.0000 × 10^0^**	8.6083 × 10^0^	6.1082 × 10^2^	4.5710 × 10^1^	5.6928 × 10^2^	5.6043 × 10^2^	9.9250 × 10^1^	6.4418 × 10^1^	6.0934 × 10^2^	8.1788 × 10^1^	4.5397 × 10^1^	3.3259 × 10^1^
CEC2017-F27	Avg	3.0935 × 10^3^	3.0932 × 10^3^	3.2124 × 10^3^	**3.0904 × 10^3^**	3.1527 × 10^3^	3.1633 × 10^3^	3.1104 × 10^3^	3.0997 × 10^3^	3.1167 × 10^3^	3.0971 × 10^3^	3.0907 × 10^3^	3.0942 × 10^3^
	Std	1.9224 × 10^0^	1.9874 × 10^0^	3.9349 × 10^1^	**6.8123 × 10^−1^**	4.1575 × 10^1^	5.1850 × 10^1^	1.1068 × 10^1^	1.4918 × 10^1^	3.0260 × 10^1^	5.2218 × 10^0^	2.9150 × 10^0^	4.1747 × 10^0^
CEC2017-F28	Avg	**3.1104 × 10^3^**	3.1208 × 10^3^	3.2851 × 10^3^	3.2864 × 10^3^	3.4390 × 10^3^	3.4062 × 10^3^	3.2868 × 10^3^	3.2787 × 10^3^	3.3154 × 10^3^	3.3021 × 10^3^	3.2945 × 10^3^	3.2454 × 10^3^
	Std	5.6931 × 10^1^	7.9112 × 10^1^	**5.4405 × 10^1^**	9.7980 × 10^1^	1.7997 × 10^2^	1.4552 × 10^2^	1.1361 × 10^2^	1.4451 × 10^2^	1.5536 × 10^2^	1.3771 × 10^2^	1.4424 × 10^2^	1.4393 × 10^2^
CEC2017-F29	Avg	**3.1545 × 10^3^**	3.1647 × 10^3^	3.3282 × 10^3^	3.1768 × 10^3^	3.3750 × 10^3^	3.3327 × 10^3^	3.2404 × 10^3^	3.2036 × 10^3^	3.2840 × 10^3^	3.1797 × 10^3^	3.1663 × 10^3^	3.1565 × 10^3^
	Std	**1.1271 × 10^1^**	1.5127 × 10^1^	7.1740 × 10^1^	1.3249 × 10^1^	1.0415 × 10^2^	8.8335 × 10^1^	4.5729 × 10^1^	4.2897 × 10^1^	9.0241 × 10^1^	2.5421 × 10^1^	1.4115 × 10^1^	1.4120 × 10^1^
CEC2017-F30	Avg	3.1054 × 10^4^	**5.8042 × 10^3^**	2.1623 × 10^6^	3.9803 × 10^4^	1.1321 × 10^6^	8.6551 × 10^5^	1.0675 × 10^6^	1.9145 × 10^5^	3.2936 × 10^5^	9.4758 × 10^4^	1.9634 × 10^5^	1.5409 × 10^5^
	Std	1.4913 × 10^5^	**3.5494 × 10^3^**	1.6362 × 10^6^	3.9745 × 10^4^	1.7386 × 10^6^	1.4057 × 10^6^	8.7821 × 10^5^	3.5491 × 10^5^	5.1685 × 10^5^	2.9737 × 10^5^	4.0134 × 10^5^	3.4980 × 10^5^

Bold values indicate the best result.

**Table A3:** Error statistics of different algorithms on CEC2017 (D = 30).

ID	Metric	MSCPO	CPO	GA	DE	WOA	HHO	DBO	GJA	SSA	BBO	LSHADE	LSHADE-SPACMA
CEC2017-F1	Avg	2.2142 × 10^3^	2.1257 × 10^3^	4.0850 × 10^9^	5.3168 × 10^4^	1.8575 × 10^9^	2.8690 × 10^7^	3.0544 × 10^10^	1.3582 × 10^6^	4.6115 × 10^3^	1.5627 × 10^4^	**1.0007 × 10^2^**	4.5862 × 10^3^
	Std	2.3215 × 10^3^	2.4369 × 10^3^	3.1941 × 10^9^	2.9695 × 10^4^	8.7476 × 10^8^	6.2226 × 10^6^	4.8707 × 10^9^	2.6271 × 10^5^	6.0240 × 10^3^	1.1423 × 10^4^	**1.0343 × 10^−1^**	6.0219 × 10^3^
CEC2017-F3	Avg	7.7012 × 10^3^	3.8271 × 10^4^	2.5055 × 10^5^	1.5424 × 10^5^	2.6005 × 10^5^	4.1066 × 10^4^	7.3766 × 10^4^	9.4120 × 10^3^	4.2652 × 10^4^	**2.1573 × 10^3^**	5.3000 × 10^4^	4.5116 × 10^4^
	Std	3.4341 × 10^3^	7.9245 × 10^3^	6.2742 × 10^4^	2.2090 × 10^4^	7.3935 × 10^4^	7.5710 × 10^3^	7.3517 × 10^3^	4.1870 × 10^3^	5.5319 × 10^3^	**1.2481 × 10^3^**	1.3329 × 10^4^	6.0124 × 10^4^
CEC2017-F4	Avg	**4.6527 × 10^2^**	4.9132 × 10^2^	9.4930 × 10^2^	4.9970 × 10^2^	8.7928 × 10^2^	5.5520 × 10^2^	6.4786 × 10^3^	5.0249 × 10^2^	4.9296 × 10^2^	4.9389 × 10^2^	4.9316 × 10^2^	4.8843 × 10^2^
	Std	3.3608 × 10^1^	2.4269 × 10^1^	1.9870 × 10^2^	**6.7247 × 10^0^**	1.8382 × 10^2^	3.2569 × 10^1^	2.3042 × 10^3^	1.9012 × 10^1^	3.2727 × 10^1^	2.9371 × 10^1^	1.8200 × 10^1^	2.6225 × 10^1^
CEC2017-F5	Avg	6.0418 × 10^2^	6.5357 × 10^2^	9.6277 × 10^2^	6.7971 × 10^2^	8.5422 × 10^2^	7.5235 × 10^2^	8.2807 × 10^2^	5.7085 × 10^2^	7.7394 × 10^2^	5.8775 × 10^2^	6.4872 × 10^2^	**5.5207 × 10^2^**
	Std	3.5262 × 10^1^	1.9062 × 10^1^	6.1231 × 10^1^	9.3155 × 10^0^	7.0634 × 10^1^	3.3563 × 10^1^	2.2118 × 10^1^	1.9636 × 10^1^	3.4217 × 10^1^	1.9553 × 10^1^	1.3528 × 10^1^	**8.6919 × 10^0^**
CEC2017-F6	Avg	6.0001 × 10^2^	6.0005 × 10^2^	7.2011 × 10^2^	**6.0001 × 10^2^**	6.7918 × 10^2^	6.6706 × 10^2^	6.6972 × 10^2^	6.0653 × 10^2^	6.5133 × 10^2^	6.0982 × 10^2^	6.0001 × 10^2^	6.0123 × 10^2^
	Std	3.1733 × 10^−2^	3.2837 × 10^−2^	1.5078 × 10^1^	**2.2390 × 10^−3^**	1.3127 × 10^1^	5.5994 × 10^0^	6.1817 × 10^0^	1.4014 × 10^0^	1.1414 × 10^1^	6.8266 × 10^0^	4.3004 × 10^−2^	1.1594 × 10^0^
CEC2017-F7	Avg	8.6622 × 10^2^	9.0162 × 10^2^	1.7072 × 10^3^	9.1437 × 10^2^	1.2818 × 10^3^	1.2723 × 10^3^	1.2156 × 10^3^	8.1138 × 10^2^	1.2694 × 10^3^	8.5849 × 10^2^	8.8752 × 10^2^	**8.0032 × 10^2^**
	Std	2.6474 × 10^1^	1.8556 × 10^1^	2.1844 × 10^2^	**1.3390 × 10^1^**	7.6928 × 10^1^	7.5810 × 10^1^	4.1315 × 10^1^	1.9834 × 10^1^	6.6526 × 10^1^	5.0019 × 10^1^	1.5293 × 10^1^	1.9042 × 10^1^
CEC2017-F8	Avg	9.0521 × 10^2^	9.3991 × 10^2^	1.1988 × 10^3^	9.7929 × 10^2^	1.0402 × 10^3^	9.8033 × 10^2^	1.0610 × 10^3^	8.6888 × 10^2^	9.8920 × 10^2^	8.8229 × 10^2^	9.4589 × 10^2^	**8.4752 × 10^2^**
	Std	3.9919 × 10^1^	2.8745 × 10^1^	4.2696 × 10^1^	1.1874 × 10^1^	4.5553 × 10^1^	2.4536 × 10^1^	2.0065 × 10^1^	1.8574 × 10^1^	2.3317 × 10^1^	2.0520 × 10^1^	1.5281 × 10^1^	**8.6241 × 10^0^**
CEC2017-F9	Avg	9.1964 × 10^2^	9.8095 × 10^2^	6.3668 × 10^3^	1.6067 × 10^3^	1.0939 × 10^4^	8.2022 × 10^3^	7.8346 × 10^3^	1.0798 × 10^3^	5.4344 × 10^3^	1.8668 × 10^3^	**9.0222 × 10^2^**	1.1033 × 10^3^
	Std	2.8102 × 10^1^	1.1698 × 10^2^	3.3252 × 10^3^	3.6154 × 10^2^	4.8439 × 10^3^	9.6581 × 10^2^	1.2164 × 10^3^	2.2394 × 10^2^	2.1292 × 10^2^	6.8894 × 10^2^	**3.1811 × 10^0^**	1.9555 × 10^2^
CEC2017-F10	Avg	6.8933 × 10^3^	6.8642 × 10^3^	7.7862 × 10^3^	7.2594 × 10^3^	7.1360 × 10^3^	5.6712 × 10^3^	8.1932 × 10^3^	4.1337 × 10^3^	5.4195 × 10^3^	4.4284 × 10^3^	7.7917 × 10^3^	**3.8669 × 10^3^**
	Std	4.4186 × 10^2^	3.6717 × 10^2^	7.5512 × 10^2^	**2.4172 × 10^2^**	7.2985 × 10^2^	7.0388 × 10^2^	6.8978 × 10^2^	6.4505 × 10^2^	5.6458 × 10^2^	5.9356 × 10^2^	2.8883 × 10^2^	4.2288 × 10^2^
CEC2017-F11	Avg	**1.2099 × 10^3^**	1.3313 × 10^3^	3.3327 × 10^4^	5.2010 × 10^3^	5.8647 × 10^3^	1.4171 × 10^3^	6.7692 × 10^3^	1.2190 × 10^3^	1.3340 × 10^3^	1.2681 × 10^3^	1.2705 × 10^3^	2.4280 × 10^3^
	Std	4.5642 × 10^1^	8.6188 × 10^1^	1.3605 × 10^4^	1.4517 × 10^3^	2.6176 × 10^3^	1.0750 × 10^2^	1.4504 × 10^3^	**4.0785 × 10^1^**	7.4719 × 10^1^	5.0760 × 10^1^	4.2962 × 10^1^	2.1531 × 10^3^
CEC2017-F12	Avg	**4.9618 × 10^4^**	3.7514 × 10^5^	7.3129 × 10^7^	2.9712 × 10^7^	2.4105 × 10^8^	2.5227 × 10^7^	6.1664 × 10^9^	3.8797 × 10^6^	1.1741 × 10^6^	1.7908 × 10^6^	1.1630 × 10^5^	7.2011 × 10^4^
	Std	**4.5679 × 10^4^**	3.2505 × 10^5^	7.8152 × 10^7^	1.3188 × 10^7^	1.3856 × 10^8^	1.8083 × 10^7^	1.7551 × 10^9^	2.3954 × 10^6^	8.1051 × 10^5^	1.2694 × 10^6^	1.6273 × 10^5^	6.0665 × 10^4^
CEC2017-F13	Avg	**7.8124 × 10^3^**	8.8705 × 10^3^	9.5615 × 10^6^	1.4749 × 10^6^	2.1449 × 10^6^	7.2247 × 10^5^	3.1524 × 10^9^	1.4008 × 10^5^	1.9948 × 10^4^	4.2308 × 10^4^	2.6074 × 10^4^	8.1440 × 10^3^
	Std	7.3380 × 10^3^	**6.9861 × 10^3^**	1.5818 × 10^7^	8.3549 × 10^5^	2.5220 × 10^6^	4.6397 × 10^5^	1.8456 × 10^9^	4.5314 × 10^4^	1.8501 × 10^4^	2.2188 × 10^4^	1.7307 × 10^4^	7.3695 × 10^3^
CEC2017-F14	Avg	**1.4776 × 10^3^**	1.5277 × 10^3^	6.6783 × 10^6^	2.4279 × 10^5^	2.1863 × 10^6^	6.7345 × 10^5^	5.7756 × 10^5^	2.0225 × 10^4^	5.5748 × 10^4^	1.5838 × 10^4^	1.7138 × 10^3^	1.5955 × 10^3^
	Std	**1.6657 × 10^1^**	2.6145 × 10^1^	7.5315 × 10^6^	1.4878 × 10^5^	2.9637 × 10^6^	4.6495 × 10^5^	5.4235 × 10^5^	2.2793 × 10^4^	5.3373 × 10^4^	1.4035 × 10^4^	7.5162 × 10^2^	8.1458 × 10^1^
CEC2017-F15	Avg	**1.6394 × 10^3^**	2.3295 × 10^3^	1.3873 × 10^6^	3.1898 × 10^5^	4.6559 × 10^6^	9.8062 × 10^4^	3.3246 × 10^6^	3.9248 × 10^4^	8.6024 × 10^3^	1.4234 × 10^4^	2.1868 × 10^3^	2.0244 × 10^3^
	Std	**3.8622 × 10^1^**	1.5834 × 10^3^	3.0227 × 10^6^	1.8155 × 10^5^	1.3489 × 10^7^	6.5846 × 10^4^	3.5503 × 10^6^	1.8285 × 10^4^	8.6841 × 10^3^	8.5613 × 10^3^	3.0389 × 10^2^	3.6603 × 10^2^
CEC2017-F16	Avg	2.5324 × 10^3^	2.5770 × 10^3^	3.6671 × 10^3^	2.7026 × 10^3^	4.2999 × 10^3^	3.5137 × 10^3^	3.8512 × 10^3^	2.5597 × 10^3^	2.9080 × 10^3^	2.4366 × 10^3^	2.7400 × 10^3^	**2.3013 × 10^3^**
	Std	2.4928 × 10^2^	3.0266 × 10^2^	3.5048 × 10^2^	**1.7161 × 10^2^**	7.3201 × 10^2^	5.0532 × 10^2^	4.5970 × 10^2^	1.9971 × 10^2^	3.1702 × 10^2^	2.1439 × 10^2^	2.1560 × 10^2^	2.9727 × 10^2^
CEC2017-F17	Avg	**1.8142 × 10^3^**	1.8700 × 10^3^	2.6524 × 10^3^	2.1080 × 10^3^	2.6895 × 10^3^	2.6636 × 10^3^	2.7983 × 10^3^	2.0367 × 10^3^	2.4662 × 10^3^	2.0622 × 10^3^	1.9656 × 10^3^	1.9032 × 10^3^
	Std	**5.6596 × 10^1^**	9.0466 × 10^1^	2.2422 × 10^2^	1.0604 × 10^2^	2.7958 × 10^2^	3.3140 × 10^2^	3.1112 × 10^2^	1.7306 × 10^2^	2.4016 × 10^2^	1.3812 × 10^2^	9.6739 × 10^1^	8.6515 × 10^1^
CEC2017-F18	Avg	**1.2760 × 10^4^**	3.9395 × 10^4^	7.2858 × 10^6^	1.9027 × 10^6^	6.7120 × 10^6^	2.9165 × 10^6^	4.3680 × 10^6^	2.7438 × 10^5^	8.1597 × 10^5^	5.6917 × 10^5^	1.9737 × 10^5^	2.7115 × 10^4^
	Std	**1.0050 × 10^4^**	1.8757 × 10^4^	7.7912 × 10^6^	8.0059 × 10^5^	6.9446 × 10^6^	3.2756 × 10^6^	4.5085 × 10^6^	1.9941 × 10^5^	1.1132 × 10^6^	4.7407 × 10^5^	1.3347 × 10^5^	1.8326 × 10^4^
CEC2017-F19	Avg	**1.9466 × 10^3^**	2.3721 × 10^3^	2.6830 × 10^6^	2.3886 × 10^5^	1.2707 × 10^7^	1.0366 × 10^6^	1.6596 × 10^8^	4.8061 × 10^4^	8.6869 × 10^3^	9.9597 × 10^3^	2.1377 × 10^3^	2.6129 × 10^3^
	Std	**1.3898 × 10^1^**	7.4067 × 10^2^	3.7156 × 10^6^	1.3298 × 10^5^	1.1105 × 10^7^	8.1363 × 10^5^	1.4470 × 10^8^	2.9310 × 10^4^	1.0375 × 10^4^	6.1828 × 10^3^	1.9262 × 10^2^	2.8635 × 10^3^
CEC2017-F20	Avg	**2.2085 × 10^3^**	2.2322 × 10^3^	3.0037 × 10^3^	2.4001 × 10^3^	2.8905 × 10^3^	2.7744 × 10^3^	2.6563 × 10^3^	2.2929 × 10^3^	2.8270 × 10^3^	2.3776 × 10^3^	2.3311 × 10^3^	2.2484 × 10^3^
	Std	**7.7214 × 10^1^**	1.3377 × 10^2^	2.3492 × 10^2^	1.1343 × 10^2^	2.2572 × 10^2^	2.4798 × 10^2^	1.3570 × 10^2^	1.1186 × 10^2^	2.2322 × 10^2^	1.3096 × 10^2^	8.7262 × 10^1^	1.2348 × 10^2^
CEC2017-F21	Avg	2.5273 × 10^3^	2.6786 × 10^3^	5.5512 × 10^3^	3.4584 × 10^3^	4.7685 × 10^3^	4.0598 × 10^3^	3.2260 × 10^3^	2.6920 × 10^3^	4.1099 × 10^3^	2.9021 × 10^3^	3.3925 × 10^3^	**2.4876 × 10^3^**
	Std	5.1189 × 10^2^	7.5773 × 10^2^	1.2986 × 10^3^	6.2522 × 10^2^	8.5491 × 10^2^	7.7136 × 10^2^	3.5226 × 10^2^	4.2291 × 10^2^	7.8002 × 10^2^	4.1329 × 10^2^	6.1654 × 10^2^	**2.8928 × 10^2^**
CEC2017-F22	Avg	1.7808 × 10^4^	1.7407 × 10^4^	2.0096 × 10^4^	1.8158 × 10^4^	1.8407 × 10^4^	1.6280 × 10^4^	2.0827 × 10^4^	1.0665 × 10^4^	1.4316 × 10^4^	1.1220 × 10^4^	1.8768 × 10^4^	**1.0081 × 10^4^**
	Std	1.0354 × 10^3^	1.1122 × 10^3^	1.8324 × 10^3^	8.8924 × 10^2^	1.5905 × 10^3^	1.8959 × 10^3^	1.7090 × 10^3^	2.3913 × 10^3^	1.7101 × 10^3^	1.6547 × 10^3^	**7.8438 × 10^2^**	9.9988 × 10^2^
CEC2017-F23	Avg	3.7264 × 10^3^	4.1701 × 10^3^	6.6308 × 10^3^	4.5466 × 10^3^	6.9141 × 10^3^	7.0410 × 10^3^	6.2370 × 10^3^	3.4853 × 10^3^	5.4691 × 10^3^	3.5568 × 10^3^	4.2469 × 10^3^	**3.2576 × 10^3^**
	Std	4.8786 × 10^2^	3.9466 × 10^2^	7.7147 × 10^2^	1.2184 × 10^2^	1.0539 × 10^3^	1.0711 × 10^3^	5.4007 × 10^2^	2.1390 × 10^2^	8.8208 × 10^2^	1.9723 × 10^2^	1.4285 × 10^2^	**1.0712 × 10^2^**
CEC2017-F24	Avg	3.3418 × 10^3^	3.5330 × 10^3^	6.7142 × 10^3^	4.9167 × 10^3^	5.9926 × 10^3^	5.8762 × 10^3^	6.0606 × 10^3^	**2.9504 × 10^3^**	5.8657 × 10^3^	3.5102 × 10^3^	4.3721 × 10^3^	3.0747 × 10^3^
	Std	5.2906 × 10^2^	1.0189 × 10^3^	5.9606 × 10^2^	4.1276 × 10^2^	7.4284 × 10^2^	1.4035 × 10^2^	3.3742 × 10^2^	6.3763 × 10^2^	**1.1786 × 10^2^**	7.2166 × 10^2^	1.3097 × 10^2^	3.8879 × 10^2^
CEC2017-F25	Avg	2.8808 × 10^3^	2.8849 × 10^3^	3.9835 × 10^3^	2.8825 × 10^3^	3.1070 × 10^3^	2.9454 × 10^3^	4.0589 × 10^3^	2.9036 × 10^3^	2.8884 × 10^3^	2.8893 × 10^3^	2.8802 × 10^3^	**2.8802 × 10^3^**
	Std	2.7352 × 10^0^	8.0235 × 10^0^	4.1418 × 10^2^	2.9901 × 10^0^	5.0672 × 10^1^	2.9942 × 10^1^	2.5461 × 10^2^	1.8766 × 10^1^	8.9794 × 10^0^	1.7844 × 10^1^	1.6477 × 10^0^	**1.0601 × 10^0^**
CEC2017-F26	Avg	4.1989 × 10^3^	4.4453 × 10^3^	7.9215 × 10^3^	5.4095 × 10^3^	8.5697 × 10^3^	8.0556 × 10^3^	7.7492 × 10^3^	4.1827 × 10^3^	6.6881 × 10^3^	4.3906 × 10^3^	4.9212 × 10^3^	**4.0582 × 10^3^**
	Std	6.2863 × 10^2^	1.1380 × 10^3^	7.9363 × 10^2^	**1.0708 × 10^2^**	9.1617 × 10^2^	8.9768 × 10^2^	6.6961 × 10^2^	1.1259 × 10^3^	1.4642 × 10^3^	1.1725 × 10^3^	4.4168 × 10^2^	4.9794 × 10^2^
CEC2017-F27	Avg	3.2206 × 10^3^	3.2403 × 10^3^	3.9029 × 10^3^	3.2219 × 10^3^	3.5000 × 10^3^	3.4582 × 10^3^	3.6048 × 10^3^	3.2446 × 10^3^	3.2910 × 10^3^	3.2327 × 10^3^	**3.2160 × 10^3^**	3.2365 × 10^3^
	Std	9.9421 × 10^0^	9.2144 × 10^0^	2.3142 × 10^2^	**3.7736 × 10^0^**	2.0065 × 10^2^	1.6357 × 10^2^	1.1589 × 10^2^	1.8745 × 10^1^	4.4206 × 10^1^	1.5064 × 10^1^	9.9254 × 10^0^	2.1824 × 10^1^
CEC2017-F28	Avg	**3.2030 × 10^3^**	3.2227 × 10^3^	4.5063 × 10^3^	3.2851 × 10^3^	3.5982 × 10^3^	3.3254 × 10^3^	5.1527 × 10^3^	3.2459 × 10^3^	3.2235 × 10^3^	3.2171 × 10^3^	3.2104 × 10^3^	3.2087 × 10^3^
	Std	1.8521 × 10^1^	1.8753 × 10^1^	6.5055 × 10^2^	3.1008 × 10^1^	1.1856 × 10^2^	2.9274 × 10^1^	5.5568 × 10^2^	2.8071 × 10^1^	2.3153 × 10^1^	1.8290 × 10^1^	**1.5129 × 10^1^**	3.8265 × 10^1^
CEC2017-F29	Avg	3.5674 × 10^3^	3.7134 × 10^3^	4.8732 × 10^3^	4.0314 × 10^3^	5.2362 × 10^3^	4.6624 × 10^3^	4.8517 × 10^3^	3.6700 × 10^3^	4.2881 × 10^3^	3.7477 × 10^3^	3.7903 × 10^3^	**3.5519 × 10^3^**
	Std	**1.0443 × 10^2^**	1.4914 × 10^2^	3.5849 × 10^2^	1.1935 × 10^2^	7.3298 × 10^2^	4.2726 × 10^2^	2.4194 × 10^2^	1.6721 × 10^2^	3.6608 × 10^2^	1.7940 × 10^2^	1.3234 × 10^2^	1.0829 × 10^2^
CEC2017-F30	Avg	**9.4993 × 10^3^**	1.9732 × 10^4^	9.3020 × 10^6^	1.4777 × 10^5^	3.6812 × 10^7^	4.7265 × 10^6^	1.1903 × 10^8^	2.2622 × 10^5^	1.5015 × 10^4^	1.1773 × 10^5^	2.0614 × 10^4^	1.1779 × 10^4^
	Std	**2.7938 × 10^3^**	7.0458 × 10^3^	8.3694 × 10^6^	6.9578 × 10^4^	2.3239 × 10^7^	2.2901 × 10^6^	1.7345 × 10^8^	9.2579 × 10^4^	5.8924 × 10^3^	9.9786 × 10^4^	2.0906 × 10^4^	3.7276 × 10^3^

Bold values indicate the best result.

**Table A4:** Error statistics of different algorithms on CEC2017 (D = 50).

ID	Metric	MSCPO	CPO	GA	DE	WOA	HHO	DBO	GJA	SSA	BBO	LSHADE	LSHADE-SPACMA
CEC2017-F1	Avg	**4.7650 × 10^3^**	6.6915 × 10^5^	9.9690 × 10^10^	3.7568 × 10^7^	8.9401 × 10^9^	2.7126 × 10^8^	7.0252 × 10^10^	5.1180 × 10^6^	1.3399 × 10^5^	3.0658 × 10^5^	5.2063 × 10^3^	3.1110 × 10^4^
	Std	**5.3095 × 10^3^**	4.9110 × 10^5^	4.0732 × 10^10^	4.3049 × 10^7^	2.5771 × 10^9^	6.7104 × 10^7^	6.8282 × 10^9^	7.1472 × 10^5^	7.1654 × 10^4^	1.7142 × 10^5^	6.2875 × 10^3^	2.3708 × 10^4^
CEC2017-F3	Avg	7.0228 × 10^4^	1.4141 × 10^5^	4.2008 × 10^5^	3.4343 × 10^5^	2.7557 × 10^5^	1.3210 × 10^5^	1.9580 × 10^5^	8.0838 × 10^4^	2.3539 × 10^5^	**5.1340 × 10^4^**	1.8625 × 10^5^	1.3207 × 10^5^
	Std	1.6494 × 10^4^	2.0391 × 10^4^	7.5788 × 10^4^	3.3037 × 10^4^	1.0360 × 10^5^	2.0661 × 10^4^	3.6195 × 10^4^	1.6182 × 10^4^	6.2420 × 10^4^	**1.3532 × 10^4^**	3.2794 × 10^4^	1.0690 × 10^5^
CEC2017-F4	Avg	5.5844 × 10^2^	5.9059 × 10^2^	9.7367 × 10^3^	6.9440 × 10^2^	2.2869 × 10^3^	8.3793 × 10^2^	1.6640 × 10^4^	5.9201 × 10^2^	5.6517 × 10^2^	5.6903 × 10^2^	**5.5001 × 10^2^**	5.7397 × 10^2^
	Std	5.0278 × 10^1^	6.1330 × 10^1^	6.9842 × 10^3^	**2.4679 × 10^1^**	6.2374 × 10^2^	1.1122 × 10^2^	2.6370 × 10^3^	4.1814 × 10^1^	5.1294 × 10^1^	5.6668 × 10^1^	5.7077 × 10^1^	4.9268 × 10^1^
CEC2017-F5	Avg	7.5890 × 10^2^	8.7208 × 10^2^	1.4395 × 10^3^	9.1942 × 10^2^	1.0478 × 10^3^	9.0226 × 10^2^	1.0822 × 10^3^	6.6867 × 10^2^	8.7964 × 10^2^	6.8774 × 10^2^	8.3849 × 10^2^	**6.4241 × 10^2^**
	Std	8.0294 × 10^1^	2.6601 × 10^1^	9.8646 × 10^1^	1.7807 × 10^1^	7.2999 × 10^1^	3.1058 × 10^1^	3.1576 × 10^1^	3.4133 × 10^1^	**1.7359 × 10^1^**	3.0719 × 10^1^	1.9634 × 10^1^	2.3427 × 10^1^
CEC2017-F6	Avg	6.0048 × 10^2^	6.0182 × 10^2^	7.3871 × 10^2^	6.0087 × 10^2^	6.9049 × 10^2^	6.7587 × 10^2^	6.8382 × 10^2^	6.1553 × 10^2^	6.6435 × 10^2^	6.2659 × 10^2^	**6.0023 × 10^2^**	6.0313 × 10^2^
	Std	3.3320 × 10^−1^	1.3882 × 10^0^	1.0819 × 10^1^	**1.3723 × 10^−1^**	1.2153 × 10^1^	5.6819 × 10^0^	6.6192 × 10^0^	4.1720 × 10^0^	6.2637 × 10^0^	6.6994 × 10^0^	2.5649 × 10^−1^	1.9265 × 10^0^
CEC2017-F7	Avg	1.1247 × 10^3^	1.1917 × 10^3^	3.8269 × 10^3^	1.1938 × 10^3^	1.8496 × 10^3^	1.8467 × 10^3^	1.7580 × 10^3^	**9.5099 × 10^2^**	1.7730 × 10^3^	1.0345 × 10^3^	1.1270 × 10^3^	1.0015 × 10^3^
	Std	4.3645 × 10^1^	4.6347 × 10^1^	5.1565 × 10^2^	**1.6252 × 10^1^**	1.0575 × 10^2^	9.2931 × 10^1^	6.5451 × 10^1^	4.4255 × 10^1^	2.7520 × 10^1^	6.9556 × 10^1^	2.8580 × 10^1^	4.7205 × 10^1^
CEC2017-F8	Avg	1.0804 × 10^3^	1.1668 × 10^3^	1.6432 × 10^3^	1.2137 × 10^3^	1.3374 × 10^3^	1.2091 × 10^3^	1.3713 × 10^3^	9.6398 × 10^2^	1.2001 × 10^3^	9.9882 × 10^2^	1.1420 × 10^3^	**9.4227 × 10^2^**
	Std	8.2894 × 10^1^	2.9906 × 10^1^	8.9038 × 10^1^	2.5214 × 10^1^	6.5949 × 10^1^	3.8930 × 10^1^	3.6024 × 10^1^	3.9702 × 10^1^	2.9676 × 10^1^	4.1593 × 10^1^	1.9795 × 10^1^	**1.7274 × 10^1^**
CEC2017-F9	Avg	2.4264 × 10^3^	4.2822 × 10^3^	3.2780 × 10^4^	7.9145 × 10^3^	3.3895 × 10^4^	2.9734 × 10^4^	3.0606 × 10^4^	2.8700 × 10^3^	1.3166 × 10^4^	6.0462 × 10^3^	**1.4675 × 10^3^**	3.3985 × 10^3^
	Std	1.4911 × 10^3^	1.8566 × 10^3^	1.0276 × 10^4^	2.0529 × 10^3^	1.1636 × 10^4^	3.2786 × 10^3^	3.6840 × 10^3^	1.3373 × 10^3^	**7.2072 × 10^2^**	1.7324 × 10^3^	7.2986 × 10^2^	1.0964 × 10^3^
CEC2017-F10	Avg	1.2572 × 10^4^	1.2798 × 10^4^	1.4383 × 10^4^	1.3722 × 10^4^	1.2736 × 10^4^	9.4842 × 10^3^	1.4080 × 10^4^	**6.5686 × 10^3^**	8.8687 × 10^3^	6.7518 × 10^3^	1.3865 × 10^4^	7.1115 × 10^3^
	Std	6.9887 × 10^2^	5.0166 × 10^2^	7.2682 × 10^2^	**3.0614 × 10^2^**	1.2779 × 10^3^	8.2200 × 10^2^	1.1079 × 10^3^	1.1125 × 10^3^	7.9849 × 10^2^	9.4494 × 10^2^	4.4638 × 10^2^	5.3078 × 10^2^
CEC2017-F11	Avg	1.7654 × 10^3^	5.5029 × 10^3^	8.6075 × 10^4^	2.8588 × 10^4^	6.3940 × 10^4^	5.6766 × 10^3^	2.4855 × 10^4^	1.6344 × 10^3^	3.4854 × 10^3^	**1.5057 × 10^3^**	2.6886 × 10^3^	1.0055 × 10^4^
	Std	1.3211 × 10^2^	1.5451 × 10^3^	2.3860 × 10^4^	5.6763 × 10^3^	2.0577 × 10^4^	1.6461 × 10^3^	4.2745 × 10^3^	1.2329 × 10^2^	9.8400 × 10^2^	**9.4230 × 10^1^**	4.4080 × 10^2^	1.3566 × 10^4^
CEC2017-F12	Avg	**1.4097 × 10^6^**	3.7372 × 10^6^	4.8681 × 10^9^	4.2424 × 10^8^	1.8337 × 10^9^	1.9122 × 10^8^	4.1840 × 10^10^	2.3801 × 10^7^	9.9537 × 10^6^	1.4600 × 10^7^	1.8961 × 10^6^	5.9152 × 10^6^
	Std	**7.6393 × 10^5^**	1.5611 × 10^6^	3.0418 × 10^9^	1.5612 × 10^8^	7.6003 × 10^8^	1.0856 × 10^8^	6.2961 × 10^9^	1.0929 × 10^7^	6.1497 × 10^6^	6.9778 × 10^6^	1.2317 × 10^6^	4.7938 × 10^6^
CEC2017-F13	Avg	**2.9921 × 10^3^**	3.8559 × 10^3^	3.7661 × 10^8^	6.5580 × 10^6^	1.4299 × 10^8^	6.1898 × 10^6^	1.3785 × 10^10^	4.5204 × 10^5^	2.0503 × 10^4^	7.2381 × 10^4^	1.3755 × 10^4^	1.3695 × 10^4^
	Std	**1.4282 × 10^3^**	2.5842 × 10^3^	3.9875 × 10^8^	5.5313 × 10^6^	1.0315 × 10^8^	8.3887 × 10^6^	5.5627 × 10^9^	1.5435 × 10^5^	1.6805 × 10^4^	3.5585 × 10^4^	1.4691 × 10^4^	8.4036 × 10^3^
CEC2017-F14	Avg	**5.1856 × 10^3^**	3.3313 × 10^4^	1.5905 × 10^7^	2.2247 × 10^6^	4.9488 × 10^6^	2.3598 × 10^6^	6.7970 × 10^6^	1.3674 × 10^5^	3.9576 × 10^5^	1.9671 × 10^5^	6.5311 × 10^4^	8.9150 × 10^3^
	Std	**4.5063 × 10^3^**	5.0900 × 10^4^	1.1629 × 10^7^	9.3222 × 10^5^	4.1467 × 10^6^	1.4391 × 10^6^	4.7682 × 10^6^	8.1704 × 10^4^	2.8205 × 10^5^	9.7570 × 10^4^	3.4071 × 10^4^	1.2468 × 10^4^
CEC2017-F15	Avg	**4.2458 × 10^3^**	6.9329 × 10^3^	3.2254 × 10^7^	9.7236 × 10^5^	1.6320 × 10^7^	7.5738 × 10^5^	1.6490 × 10^9^	1.3833 × 10^5^	1.7769 × 10^4^	2.8465 × 10^4^	1.6102 × 10^4^	8.1949 × 10^3^
	Std	**2.6019 × 10^3^**	3.7176 × 10^3^	3.6351 × 10^7^	9.0927 × 10^5^	1.7179 × 10^7^	3.1654 × 10^5^	6.7887 × 10^8^	3.4378 × 10^4^	5.6826 × 10^3^	1.8834 × 10^4^	6.9753 × 10^3^	4.9935 × 10^3^
CEC2017-F16	Avg	3.0908 × 10^3^	3.5837 × 10^3^	5.5848 × 10^3^	4.5772 × 10^3^	6.0478 × 10^3^	4.4500 × 10^3^	5.5665 × 10^3^	3.1111 × 10^3^	3.9909 × 10^3^	3.0430 × 10^3^	4.0673 × 10^3^	**2.9952 × 10^3^**
	Std	6.0126 × 10^2^	4.7992 × 10^2^	6.7887 × 10^2^	**2.2161 × 10^2^**	8.0064 × 10^2^	6.1713 × 10^2^	4.8179 × 10^2^	3.3287 × 10^2^	4.6026 × 10^2^	4.0817 × 10^2^	4.3705 × 10^2^	3.2827 × 10^2^
CEC2017-F17	Avg	**2.7500 × 10^3^**	2.8468 × 10^3^	4.3192 × 10^3^	3.5132 × 10^3^	4.4400 × 10^3^	3.8590 × 10^3^	4.7832 × 10^3^	2.9821 × 10^3^	3.6113 × 10^3^	3.0211 × 10^3^	3.4169 × 10^3^	2.8907 × 10^3^
	Std	2.6686 × 10^2^	2.8180 × 10^2^	5.9230 × 10^2^	**1.4238 × 10^2^**	5.4870 × 10^2^	4.4333 × 10^2^	4.6612 × 10^2^	3.0536 × 10^2^	3.0943 × 10^2^	3.0688 × 10^2^	2.0272 × 10^2^	2.5951 × 10^2^
CEC2017-F18	Avg	**1.5916 × 10^5^**	7.8502 × 10^5^	6.0229 × 10^7^	1.3833 × 10^7^	4.1915 × 10^7^	5.4105 × 10^6^	1.4495 × 10^7^	1.7391 × 10^6^	2.5137 × 10^6^	2.2098 × 10^6^	1.7417 × 10^6^	3.2543 × 10^5^
	Std	**1.1082 × 10^5^**	7.2060 × 10^5^	5.0107 × 10^7^	6.3456 × 10^6^	3.3123 × 10^7^	4.4868 × 10^6^	1.1631 × 10^7^	9.2519 × 10^5^	1.4504 × 10^6^	1.2425 × 10^6^	1.1453 × 10^6^	1.1783 × 10^6^
CEC2017-F19	Avg	**1.1174 × 10^4^**	2.1489 × 10^4^	2.3930 × 10^7^	2.6490 × 10^5^	8.4171 × 10^6^	1.9501 × 10^6^	1.1591 × 10^9^	1.0609 × 10^5^	1.7386 × 10^4^	3.1929 × 10^4^	1.8920 × 10^4^	1.4490 × 10^4^
	Std	**7.1319 × 10^3^**	8.2980 × 10^3^	1.6381 × 10^7^	1.9257 × 10^5^	8.2281 × 10^6^	1.7254 × 10^6^	7.4986 × 10^8^	3.7662 × 10^4^	1.2810 × 10^4^	1.6103 × 10^4^	1.5687 × 10^4^	1.0310 × 10^4^
CEC2017-F20	Avg	3.0767 × 10^3^	3.1667 × 10^3^	4.0784 × 10^3^	3.4911 × 10^3^	3.7239 × 10^3^	3.4998 × 10^3^	3.7955 × 10^3^	**2.7965 × 10^3^**	3.7485 × 10^3^	3.0221 × 10^3^	3.5375 × 10^3^	2.9951 × 10^3^
	Std	3.4757 × 10^2^	3.7954 × 10^2^	4.7087 × 10^2^	1.6492 × 10^2^	3.5064 × 10^2^	3.4082 × 10^2^	3.0165 × 10^2^	2.9759 × 10^2^	3.1536 × 10^2^	3.1131 × 10^2^	**1.5189 × 10^2^**	2.2697 × 10^2^
CEC2017-F21	Avg	4.6023 × 10^3^	4.8088 × 10^3^	1.0817 × 10^4^	6.4835 × 10^3^	7.9930 × 10^3^	6.2291 × 10^3^	7.0711 × 10^3^	3.9536 × 10^3^	6.1127 × 10^3^	4.2081 × 10^3^	5.5775 × 10^3^	**3.4749 × 10^3^**
	Std	1.1719 × 10^3^	1.7092 × 10^3^	1.5798 × 10^3^	3.5284 × 10^2^	1.0509 × 10^3^	**2.8776 × 10^2^**	8.1544 × 10^2^	1.0541 × 10^3^	3.1032 × 10^2^	4.7044 × 10^2^	6.3316 × 10^2^	5.9767 × 10^2^
CEC2017-F22	Avg	3.3650 × 10^4^	3.3591 × 10^4^	3.8148 × 10^4^	3.4952 × 10^4^	3.2967 × 10^4^	2.9067 × 10^4^	3.6608 × 10^4^	**1.7999 × 10^4^**	2.3457 × 10^4^	1.8155 × 10^4^	3.5646 × 10^4^	1.9106 × 10^4^
	Std	1.4046 × 10^3^	1.2684 × 10^3^	2.2838 × 10^3^	**9.8909 × 10^2^**	2.7331 × 10^3^	2.9961 × 10^3^	2.3915 × 10^3^	2.2894 × 10^3^	2.3031 × 10^3^	1.6650 × 10^3^	1.1230 × 10^3^	1.2225 × 10^3^
CEC2017-F23	Avg	5.6036 × 10^3^	6.7108 × 10^3^	1.2219 × 10^4^	7.1910 × 10^3^	1.1770 × 10^4^	1.1414 × 10^4^	1.0731 × 10^4^	4.6248 × 10^3^	9.1393 × 10^3^	5.1431 × 10^3^	6.4778 × 10^3^	**4.5975 × 10^3^**
	Std	8.8008 × 10^2^	3.0961 × 10^2^	1.3323 × 10^3^	**1.5114 × 10^2^**	1.4462 × 10^3^	1.1037 × 10^3^	5.3892 × 10^2^	3.6999 × 10^2^	1.1208 × 10^3^	4.4733 × 10^2^	2.3581 × 10^2^	2.6488 × 10^2^
CEC2017-F24	Avg	5.3993 × 10^3^	6.6485 × 10^3^	1.1623 × 10^4^	7.5307 × 10^3^	9.2170 × 10^3^	8.3018 × 10^3^	9.4716 × 10^3^	5.6416 × 10^3^	8.2545 × 10^3^	5.2589 × 10^3^	6.2963 × 10^3^	**4.3898 × 10^3^**
	Std	8.8212 × 10^2^	1.2352 × 10^3^	9.6280 × 10^2^	2.1654 × 10^2^	8.8243 × 10^2^	2.3113 × 10^2^	**1.6660 × 10^2^**	1.5178 × 10^3^	2.0701 × 10^2^	7.1997 × 10^2^	2.4117 × 10^2^	4.4445 × 10^2^
CEC2017-F25	Avg	3.0778 × 10^3^	3.1353 × 10^3^	1.1577 × 10^4^	3.1052 × 10^3^	4.1457 × 10^3^	3.2518 × 10^3^	1.1010 × 10^4^	3.1365 × 10^3^	3.1047 × 10^3^	3.0646 × 10^3^	**3.0626 × 10^3^**	3.0668 × 10^3^
	Std	3.1673 × 10^1^	2.1742 × 10^1^	3.3695 × 10^3^	2.3661 × 10^1^	2.7197 × 10^2^	5.1214 × 10^1^	1.1642 × 10^3^	3.3040 × 10^1^	**1.9946 × 10^1^**	2.8924 × 10^1^	2.8202 × 10^1^	2.7543 × 10^1^
CEC2017-F26	Avg	5.9650 × 10^3^	7.3521 × 10^3^	1.4674 × 10^4^	7.7822 × 10^3^	1.4645 × 10^4^	1.1357 × 10^4^	1.3815 × 10^4^	**4.7604 × 10^3^**	8.5778 × 10^3^	6.4353 × 10^3^	7.1025 × 10^3^	5.9975 × 10^3^
	Std	1.0411 × 10^3^	1.6800 × 10^3^	1.7343 × 10^3^	**2.1290 × 10^2^**	1.4684 × 10^3^	1.6223 × 10^3^	8.8641 × 10^2^	2.3844 × 10^3^	3.8077 × 10^3^	1.4889 × 10^3^	3.8225 × 10^2^	7.4246 × 10^2^
CEC2017-F27	Avg	3.4044 × 10^3^	3.4958 × 10^3^	5.4041 × 10^3^	3.4736 × 10^3^	4.6483 × 10^3^	4.5819 × 10^3^	4.9553 × 10^3^	3.4707 × 10^3^	3.8547 × 10^3^	3.3934 × 10^3^	**3.3801 × 10^3^**	3.5277 × 10^3^
	Std	6.7468 × 10^1^	6.7283 × 10^1^	5.6351 × 10^2^	**4.7113 × 10^1^**	5.2570 × 10^2^	4.3275 × 10^2^	3.5834 × 10^2^	1.3003 × 10^2^	3.0059 × 10^2^	5.1638 × 10^1^	8.2511 × 10^1^	9.8351 × 10^1^
CEC2017-F28	Avg	3.3575 × 10^3^	3.4502 × 10^3^	1.0454 × 10^4^	3.4471 × 10^3^	5.2203 × 10^3^	3.8273 × 10^3^	8.1364 × 10^3^	3.4114 × 10^3^	3.3647 × 10^3^	**3.3133 × 10^3^**	3.3222 × 10^3^	3.3567 × 10^3^
	Std	2.9537 × 10^1^	4.6058 × 10^1^	1.5769 × 10^3^	6.3226 × 10^1^	3.9721 × 10^2^	1.5127 × 10^2^	7.6229 × 10^2^	4.8431 × 10^1^	3.9677 × 10^1^	3.9005 × 10^1^	**1.5995 × 10^1^**	4.1470 × 10^1^
CEC2017-F29	Avg	3.9994 × 10^3^	4.4925 × 10^3^	7.1790 × 10^3^	5.2465 × 10^3^	8.9688 × 10^3^	6.1490 × 10^3^	1.0624 × 10^4^	4.3802 × 10^3^	5.1137 × 10^3^	4.5546 × 10^3^	4.5836 × 10^3^	**3.9903 × 10^3^**
	Std	2.7685 × 10^2^	3.0103 × 10^2^	8.0311 × 10^2^	2.6462 × 10^2^	1.5255 × 10^3^	7.0345 × 10^2^	1.9484 × 10^3^	2.9359 × 10^2^	3.9852 × 10^2^	**2.5908 × 10^2^**	3.1932 × 10^2^	2.7081 × 10^2^
CEC2017-F30	Avg	1.1377 × 10^6^	1.9051 × 10^6^	3.9192 × 10^8^	1.3754 × 10^7^	2.7526 × 10^8^	6.7289 × 10^7^	1.7362 × 10^9^	3.2906 × 10^6^	1.3493 × 10^6^	9.4474 × 10^6^	**1.0803 × 10^6^**	1.5865 × 10^6^
	Std	**2.3226 × 10^5^**	6.2518 × 10^5^	1.9481 × 10^8^	5.4033 × 10^6^	1.2522 × 10^8^	2.3776 × 10^7^	1.1883 × 10^9^	5.8716 × 10^5^	5.3601 × 10^5^	1.5224 × 10^6^	5.4471 × 10^5^	4.5749 × 10^5^

Bold values indicate the best result.

**Table A5:** Error statistics of different algorithms on CEC2017 (D = 100).

ID	Metric	MSCPO	CPO	GA	DE	WOA	HHO	DBO	GJA	SSA	BBO	LSHADE	LSHADE-SPACMA
CEC2017-F1	Avg	1.2701 × 10^8^	1.1138 × 10^9^	4.5232 × 10^11^	2.8907 × 10^9^	6.3413 × 10^10^	7.1047 × 10^9^	2.2225 × 10^11^	6.0541 × 10^7^	8.8652 × 10^7^	1.0566 × 10^7^	**4.8709 × 10^6^**	1.9749 × 10^8^
	Std	6.8553 × 10^7^	9.9604 × 10^8^	6.6935 × 10^10^	1.0483 × 10^9^	1.0534 × 10^10^	1.5085 × 10^9^	9.4099 × 10^9^	1.3287 × 10^7^	3.9860 × 10^7^	**2.7847 × 10^6^**	6.8899 × 10^6^	1.0162 × 10^8^
CEC2017-F3	Avg	**2.8813 × 10^5^**	4.0520 × 10^5^	8.8727 × 10^5^	8.2911 × 10^5^	8.9874 × 10^5^	3.1624 × 10^5^	4.6507 × 10^5^	3.3101 × 10^5^	4.9189 × 10^5^	3.9369 × 10^5^	5.5462 × 10^5^	4.2622 × 10^5^
	Std	3.1412 × 10^4^	3.3551 × 10^4^	1.3937 × 10^5^	9.5300 × 10^4^	1.4957 × 10^5^	**1.4244 × 10^4^**	1.1221 × 10^5^	4.0688 × 10^4^	5.9345 × 10^4^	6.8267 × 10^4^	6.5778 × 10^4^	1.7550 × 10^5^
CEC2017-F4	Avg	9.5403 × 10^2^	1.2114 × 10^3^	1.0181 × 10^5^	1.7183 × 10^3^	1.2323 × 10^4^	2.5580 × 10^3^	5.9257 × 10^4^	9.9606 × 10^2^	9.8377 × 10^2^	**7.9212 × 10^2^**	8.1872 × 10^2^	9.6634 × 10^2^
	Std	1.0366 × 10^2^	1.1634 × 10^2^	2.9901 × 10^4^	1.7309 × 10^2^	2.8080 × 10^3^	3.6173 × 10^2^	7.8191 × 10^3^	7.4607 × 10^1^	7.9217 × 10^1^	**4.9189 × 10^1^**	5.8770 × 10^1^	8.0355 × 10^1^
CEC2017-F5	Avg	1.4013 × 10^3^	1.5769 × 10^3^	2.9032 × 10^3^	1.6310 × 10^3^	1.8496 × 10^3^	1.5945 × 10^3^	1.9329 × 10^3^	**9.9218 × 10^2^**	1.3685 × 10^3^	1.0849 × 10^3^	1.4473 × 10^3^	1.0751 × 10^3^
	Std	1.2713 × 10^2^	7.2099 × 10^1^	1.7532 × 10^2^	**3.3078 × 10^1^**	1.1353 × 10^2^	5.1306 × 10^1^	4.9000 × 10^1^	9.8830 × 10^1^	4.6953 × 10^1^	8.7574 × 10^1^	3.8671 × 10^1^	9.2773 × 10^1^
CEC2017-F6	Avg	6.1505 × 10^2^	6.2119 × 10^2^	7.5659 × 10^2^	6.1893 × 10^2^	7.0134 × 10^2^	6.8671 × 10^2^	6.9726 × 10^2^	6.3016 × 10^2^	6.6530 × 10^2^	6.4494 × 10^2^	**6.0745 × 10^2^**	6.1553 × 10^2^
	Std	3.4835 × 10^0^	5.1188 × 10^0^	8.4711 × 10^0^	**1.4928 × 10^0^**	8.7251 × 10^0^	4.3657 × 10^0^	3.4944 × 10^0^	3.5775 × 10^0^	2.2487 × 10^0^	5.9429 × 10^0^	3.0241 × 10^0^	4.1403 × 10^0^
CEC2017-F7	Avg	1.9972 × 10^3^	2.1761 × 10^3^	1.0939 × 10^4^	2.1251 × 10^3^	3.7181 × 10^3^	3.7347 × 10^3^	3.6280 × 10^3^	**1.5146 × 10^3^**	3.2753 × 10^3^	1.8404 × 10^3^	2.0012 × 10^3^	2.0421 × 10^3^
	Std	1.1698 × 10^2^	9.7010 × 10^1^	1.1371 × 10^3^	**4.6347 × 10^1^**	1.7377 × 10^2^	1.3154 × 10^2^	9.0079 × 10^1^	8.3278 × 10^1^	7.9512 × 10^1^	2.5999 × 10^2^	1.0258 × 10^2^	1.9254 × 10^2^
CEC2017-F8	Avg	1.6780 × 10^3^	1.8504 × 10^3^	3.2148 × 10^3^	1.9324 × 10^3^	2.3152 × 10^3^	2.0340 × 10^3^	2.3322 × 10^3^	**1.2844 × 10^3^**	1.8498 × 10^3^	1.3861 × 10^3^	1.7421 × 10^3^	1.3783 × 10^3^
	Std	1.5143 × 10^2^	6.6795 × 10^1^	2.0091 × 10^2^	**3.9529 × 10^1^**	1.3760 × 10^2^	5.8349 × 10^1^	7.0194 × 10^1^	7.6786 × 10^1^	4.4657 × 10^1^	8.2525 × 10^1^	4.5899 × 10^1^	9.7207 × 10^1^
CEC2017-F9	Avg	2.7627 × 10^4^	4.0582 × 10^4^	1.3782 × 10^5^	5.2905 × 10^4^	7.2508 × 10^4^	6.3180 × 10^4^	7.6051 × 10^4^	2.1871 × 10^4^	2.4921 × 10^4^	2.3334 × 10^4^	**1.3467 × 10^4^**	2.2034 × 10^4^
	Std	6.1878 × 10^3^	5.0439 × 10^3^	2.4246 × 10^4^	6.9337 × 10^3^	2.0754 × 10^4^	4.5858 × 10^3^	6.4651 × 10^3^	5.6326 × 10^3^	**5.4601 × 10^2^**	4.0579 × 10^3^	5.5337 × 10^3^	5.8177 × 10^3^
CEC2017-F10	Avg	2.9692 × 10^4^	2.9463 × 10^4^	3.2424 × 10^4^	3.1754 × 10^4^	2.7659 × 10^4^	2.2785 × 10^4^	3.1093 × 10^4^	**1.4123 × 10^4^**	1.7588 × 10^4^	1.4630 × 10^4^	3.1488 × 10^4^	1.9417 × 10^4^
	Std	8.8045 × 10^2^	6.5356 × 10^2^	1.0151 × 10^3^	5.7956 × 10^2^	1.8443 × 10^3^	1.1431 × 10^3^	1.2173 × 10^3^	1.2816 × 10^3^	1.4807 × 10^3^	1.0939 × 10^3^	**4.1840 × 10^2^**	1.6947 × 10^3^
CEC2017-F11	Avg	5.4010 × 10^4^	1.4751 × 10^5^	5.9343 × 10^5^	3.8504 × 10^5^	3.0424 × 10^5^	9.6788 × 10^4^	1.8587 × 10^5^	3.9284 × 10^4^	1.5582 × 10^5^	**1.9289 × 10^4^**	1.5393 × 10^5^	1.4830 × 10^5^
	Std	9.5361 × 10^3^	1.8515 × 10^4^	1.4287 × 10^5^	5.0119 × 10^4^	1.1249 × 10^5^	1.7123 × 10^4^	2.1232 × 10^4^	9.7679 × 10^3^	2.2712 × 10^4^	**5.7574 × 10^3^**	2.5620 × 10^4^	1.2877 × 10^5^
CEC2017-F12	Avg	3.2418 × 10^7^	1.1401 × 10^8^	1.6375 × 10^11^	3.9752 × 10^9^	1.3002 × 10^10^	1.3683 × 10^9^	1.2723 × 10^11^	1.8701 × 10^8^	1.2000 × 10^8^	1.5223 × 10^8^	**1.9574 × 10^7^**	2.0890 × 10^8^
	Std	**1.3074 × 10^7^**	3.3589 × 10^7^	5.0683 × 10^10^	6.1004 × 10^8^	3.6277 × 10^9^	4.6261 × 10^8^	1.3398 × 10^10^	6.1129 × 10^7^	7.0411 × 10^7^	5.9585 × 10^7^	1.9424 × 10^7^	1.1495 × 10^8^
CEC2017-F13	Avg	**7.4980 × 10^3^**	8.4994 × 10^3^	3.1108 × 10^10^	3.2949 × 10^6^	7.5206 × 10^8^	1.8581 × 10^7^	3.0491 × 10^10^	9.5382 × 10^5^	2.8394 × 10^4^	6.8695 × 10^4^	8.3729 × 10^3^	8.7352 × 10^4^
	Std	4.1625 × 10^3^	**2.7723 × 10^3^**	1.3405 × 10^10^	5.9783 × 10^6^	3.5623 × 10^8^	4.6441 × 10^6^	3.6630 × 10^9^	1.6060 × 10^5^	1.1832 × 10^4^	2.7468 × 10^4^	3.6506 × 10^3^	1.1329 × 10^5^
CEC2017-F14	Avg	4.6911 × 10^5^	1.6863 × 10^6^	9.3110 × 10^7^	3.6340 × 10^7^	1.3004 × 10^7^	5.5380 × 10^6^	2.5357 × 10^7^	1.8660 × 10^6^	1.9371 × 10^6^	1.9558 × 10^6^	2.2157 × 10^6^	**4.6305 × 10^5^**
	Std	**2.2471 × 10^5^**	6.0757 × 10^5^	6.2776 × 10^7^	1.0967 × 10^7^	6.8054 × 10^6^	2.1601 × 10^6^	1.2583 × 10^7^	5.5252 × 10^5^	1.0049 × 10^6^	5.7638 × 10^5^	1.2375 × 10^6^	3.5608 × 10^5^
CEC2017-F15	Avg	**3.3766 × 10^3^**	3.6367 × 10^3^	4.0026 × 10^9^	4.1738 × 10^6^	1.2211 × 10^8^	4.2001 × 10^6^	1.1094 × 10^10^	3.6963 × 10^5^	1.0177 × 10^4^	4.5049 × 10^4^	5.2915 × 10^3^	1.0091 × 10^4^
	Std	**1.5706 × 10^3^**	1.7913 × 10^3^	2.8796 × 10^9^	4.6077 × 10^6^	9.1026 × 10^7^	1.2887 × 10^6^	2.3587 × 10^9^	7.5760 × 10^4^	5.3278 × 10^3^	2.0120 × 10^4^	4.2847 × 10^3^	4.5352 × 10^3^
CEC2017-F16	Avg	7.8186 × 10^3^	9.1635 × 10^3^	1.7483 × 10^4^	1.1184 × 10^4^	1.5729 × 10^4^	8.8168 × 10^3^	1.6249 × 10^4^	5.7003 × 10^3^	6.3152 × 10^3^	5.6163 × 10^3^	8.9219 × 10^3^	**5.4772 × 10^3^**
	Std	1.5412 × 10^3^	5.4547 × 10^2^	3.5943 × 10^3^	**3.6526 × 10^2^**	2.1188 × 10^3^	7.8236 × 10^2^	1.4350 × 10^3^	6.5925 × 10^2^	7.7871 × 10^2^	8.7931 × 10^2^	4.4221 × 10^2^	7.5669 × 10^2^
CEC2017-F17	Avg	5.6173 × 10^3^	6.2777 × 10^3^	1.4027 × 10^4^	7.7639 × 10^3^	1.3596 × 10^4^	6.8174 × 10^3^	8.2229 × 10^4^	5.2902 × 10^3^	6.1276 × 10^3^	**4.7563 × 10^3^**	6.5941 × 10^3^	5.0768 × 10^3^
	Std	7.9345 × 10^2^	**2.8262 × 10^2^**	1.5010 × 10^4^	2.9988 × 10^2^	8.8913 × 10^3^	7.8199 × 10^2^	7.6044 × 10^4^	4.9333 × 10^2^	6.5858 × 10^2^	5.0227 × 10^2^	2.9901 × 10^2^	4.8688 × 10^2^
CEC2017-F18	Avg	1.0813 × 10^6^	2.4812 × 10^6^	8.1950 × 10^7^	7.0192 × 10^7^	1.2183 × 10^7^	5.8297 × 10^6^	3.5956 × 10^7^	2.2841 × 10^6^	2.6029 × 10^6^	3.9889 × 10^6^	5.7476 × 10^6^	**5.4289 × 10^5^**
	Std	7.0130 × 10^5^	1.0646 × 10^6^	5.1153 × 10^7^	1.9672 × 10^7^	5.8963 × 10^6^	2.4408 × 10^6^	1.8397 × 10^7^	6.1524 × 10^5^	1.3334 × 10^6^	1.6816 × 10^6^	2.6941 × 10^6^	**2.6153 × 10^5^**
CEC2017-F19	Avg	**3.4315 × 10^3^**	3.4672 × 10^3^	4.8042 × 10^9^	5.2201 × 10^6^	1.5203 × 10^8^	1.6813 × 10^7^	9.0696 × 10^9^	7.4783 × 10^5^	6.4428 × 10^3^	4.8084 × 10^5^	5.4243 × 10^3^	8.5867 × 10^3^
	Std	2.0912 × 10^3^	**1.1943 × 10^3^**	3.6573 × 10^9^	4.7932 × 10^6^	1.1624 × 10^8^	7.5953 × 10^6^	2.7698 × 10^9^	2.4031 × 10^5^	6.8515 × 10^3^	2.9005 × 10^5^	3.6469 × 10^3^	7.1556 × 10^3^
CEC2017-F20	Avg	6.4975 × 10^3^	6.7526 × 10^3^	7.9528 × 10^3^	7.3851 × 10^3^	6.9086 × 10^3^	6.0497 × 10^3^	7.3168 × 10^3^	**4.7908 × 10^3^**	6.1338 × 10^3^	4.8636 × 10^3^	7.1456 × 10^3^	5.9049 × 10^3^
	Std	6.3102 × 10^2^	3.1485 × 10^2^	6.8663 × 10^2^	2.8324 × 10^2^	5.4989 × 10^2^	5.1158 × 10^2^	4.1474 × 10^2^	5.3416 × 10^2^	6.8093 × 10^2^	5.8773 × 10^2^	**2.8056 × 10^2^**	4.3728 × 10^2^
CEC2017-F21	Avg	1.0227 × 10^4^	1.2190 × 10^4^	2.6740 × 10^4^	1.3919 × 10^4^	1.7615 × 10^4^	1.5065 × 10^4^	1.8318 × 10^4^	**6.9153 × 10^3^**	1.2534 × 10^4^	7.9146 × 10^3^	1.1745 × 10^4^	8.0304 × 10^3^
	Std	1.8147 × 10^3^	4.2747 × 10^2^	1.8922 × 10^3^	**3.3733 × 10^2^**	1.0966 × 10^3^	1.0553 × 10^3^	7.2445 × 10^2^	7.0400 × 10^2^	1.1863 × 10^3^	8.5967 × 10^2^	3.8680 × 10^2^	1.0582 × 10^3^
CEC2017-F22	Avg	7.8809 × 10^4^	7.8178 × 10^4^	8.6996 × 10^4^	8.0495 × 10^4^	7.4772 × 10^4^	6.6609 × 10^4^	7.9341 × 10^4^	4.2093 × 10^4^	4.8863 × 10^4^	**3.8981 × 10^4^**	7.9588 × 10^4^	5.1281 × 10^4^
	Std	2.1838 × 10^3^	2.2231 × 10^3^	2.9741 × 10^3^	**1.7274 × 10^3^**	4.4165 × 10^3^	3.7331 × 10^3^	7.7910 × 10^3^	3.5686 × 10^3^	4.0845 × 10^3^	4.3535 × 10^3^	2.0504 × 10^3^	4.1180 × 10^3^
CEC2017-F23	Avg	1.0894 × 10^4^	1.2565 × 10^4^	2.7840 × 10^4^	1.3043 × 10^4^	2.2972 × 10^4^	2.1125 × 10^4^	2.1446 × 10^4^	**7.6435 × 10^3^**	1.6528 × 10^4^	8.7006 × 10^3^	1.2131 × 10^4^	7.8418 × 10^3^
	Std	1.4916 × 10^3^	5.0827 × 10^2^	1.8126 × 10^3^	**2.8550 × 10^2^**	2.0758 × 10^3^	1.8550 × 10^3^	1.1861 × 10^3^	7.3097 × 10^2^	1.8455 × 10^3^	9.1541 × 10^2^	5.1115 × 10^2^	3.9286 × 10^2^
CEC2017-F24	Avg	1.2521 × 10^4^	1.5320 × 10^4^	3.2885 × 10^4^	1.4721 × 10^4^	2.2665 × 10^4^	2.0240 × 10^4^	2.2824 × 10^4^	1.1431 × 10^4^	1.9379 × 10^4^	**1.0347 × 10^4^**	1.2908 × 10^4^	1.1038 × 10^4^
	Std	1.6553 × 10^3^	3.4463 × 10^3^	2.2561 × 10^3^	**2.9783 × 10^2^**	1.3055 × 10^3^	6.1119 × 10^2^	5.5971 × 10^2^	4.2468 × 10^3^	7.1965 × 10^2^	1.2912 × 10^3^	4.6916 × 10^2^	1.3300 × 10^3^
CEC2017-F25	Avg	3.6172 × 10^3^	3.8895 × 10^3^	5.3289 × 10^4^	5.4798 × 10^3^	7.8490 × 10^3^	4.5165 × 10^3^	1.9248 × 10^4^	3.6803 × 10^3^	3.5893 × 10^3^	**3.4218 × 10^3^**	3.4703 × 10^3^	3.6233 × 10^3^
	Std	**5.8472 × 10^1^**	8.3122 × 10^1^	1.3954 × 10^4^	2.9856 × 10^2^	5.5670 × 10^2^	1.5445 × 10^2^	1.2836 × 10^3^	8.1869 × 10^1^	6.2250 × 10^1^	6.9247 × 10^1^	8.5059 × 10^1^	7.0563 × 10^1^
CEC2017-F26	Avg	1.4887 × 10^4^	1.8924 × 10^4^	4.6981 × 10^4^	1.6883 × 10^4^	3.6841 × 10^4^	2.8435 × 10^4^	3.8626 × 10^4^	1.5505 × 10^4^	2.4446 × 10^4^	**1.4086 × 10^4^**	1.4805 × 10^4^	1.5572 × 10^4^
	Std	2.2893 × 10^3^	1.5169 × 10^3^	4.8239 × 10^3^	**3.0219 × 10^2^**	3.3451 × 10^3^	1.7968 × 10^3^	4.1294 × 10^3^	5.2015 × 10^3^	3.9929 × 10^3^	2.4920 × 10^3^	2.1921 × 10^3^	2.6741 × 10^3^
CEC2017-F27	Avg	3.7033 × 10^3^	3.8049 × 10^3^	1.0022 × 10^4^	4.1460 × 10^3^	6.0261 × 10^3^	5.2990 × 10^3^	8.2725 × 10^3^	3.7397 × 10^3^	4.1165 × 10^3^	**3.6109 × 10^3^**	3.6145 × 10^3^	3.9611 × 10^3^
	Std	**7.7381 × 10^1^**	9.7903 × 10^1^	2.2413 × 10^3^	1.3579 × 10^2^	9.7265 × 10^2^	8.4598 × 10^2^	9.1168 × 10^2^	1.0382 × 10^2^	4.8835 × 10^2^	8.1372 × 10^1^	8.2967 × 10^1^	1.8744 × 10^2^
CEC2017-F28	Avg	3.8982 × 10^3^	4.3369 × 10^3^	4.1110 × 10^4^	1.0076 × 10^4^	1.1486 × 10^4^	5.6663 × 10^3^	2.1716 × 10^4^	3.8306 × 10^3^	3.7256 × 10^3^	**3.5084 × 10^3^**	3.6124 × 10^3^	3.7799 × 10^3^
	Std	1.2127 × 10^2^	1.5143 × 10^2^	6.0587 × 10^3^	1.8533 × 10^3^	9.3633 × 10^2^	4.6061 × 10^2^	1.9758 × 10^3^	7.1264 × 10^1^	5.6227 × 10^1^	**4.3896 × 10^1^**	6.4731 × 10^1^	8.9309 × 10^1^
CEC2017-F29	Avg	7.7284 × 10^3^	9.2312 × 10^3^	2.2438 × 10^4^	9.9807 × 10^3^	1.9045 × 10^4^	1.0998 × 10^4^	6.5193 × 10^4^	**7.3049 × 10^3^**	8.0970 × 10^3^	7.5819 × 10^3^	8.7124 × 10^3^	7.5203 × 10^3^
	Std	1.0219 × 10^3^	4.2578 × 10^2^	1.1921 × 10^4^	**3.7509 × 10^2^**	3.7301 × 10^3^	8.5332 × 10^2^	3.5300 × 10^4^	5.5987 × 10^2^	7.1321 × 10^2^	5.8173 × 10^2^	6.0006 × 10^2^	7.9185 × 10^2^
CEC2017-F30	Avg	1.2135 × 10^5^	5.5110 × 10^5^	2.0139 × 10^10^	4.6468 × 10^6^	1.5944 × 10^9^	1.4641 × 10^8^	2.6094 × 10^10^	5.2084 × 10^6^	5.8463 × 10^5^	1.0163 × 10^7^	**1.7563 × 10^4^**	1.6432 × 10^6^
	Std	6.7594 × 10^4^	3.5660 × 10^5^	1.1548 × 10^10^	1.6847 × 10^6^	5.1328 × 10^8^	6.1047 × 10^7^	2.9071 × 10^9^	1.2395 × 10^6^	3.4602 × 10^5^	4.1414 × 10^6^	**5.9044 × 10^3^**	1.5843 × 10^6^

Bold values indicate the best result.

**Table A6:** Detailed results of the Wilcoxon signed-rank test on CEC2017 (D = 10).

MSCPO vs.	CPO	GA	DE	WOA	HHO	DBO	GJA	SSA	BBO	LSHADE	LSHADE-SPACMA
CEC2017-F1	+	+	+	+	+	+	+	+	+	−	−
CEC2017-F3	+	+	+	+	+	+	+	+	+	=	=
CEC2017-F4	+	+	+	+	+	+	+	+	+	+	−
CEC2017-F5	+	+	+	+	+	+	+	+	+	+	=
CEC2017-F6	+	+	−	+	+	+	+	+	+	−	+
CEC2017-F7	+	+	+	+	+	+	+	+	+	+	−
CEC2017-F8	+	+	+	+	+	+	+	+	+	+	=
CEC2017-F9	=	+	=	+	+	+	+	+	+	=	+
CEC2017-F10	=	+	+	+	+	+	=	+	+	+	−
CEC2017-F11	+	+	+	+	+	+	+	+	+	+	+
CEC2017-F12	+	+	+	+	+	+	+	+	+	+	+
CEC2017-F13	+	+	+	+	+	+	+	+	+	+	+
CEC2017-F14	=	+	+	+	+	+	+	+	+	+	+
CEC2017-F15	+	+	+	+	+	+	+	+	+	=	+
CEC2017-F16	+	+	+	+	+	+	+	+	+	+	+
CEC2017-F17	=	+	−	+	+	+	+	+	+	+	−
CEC2017-F18	+	+	+	+	+	+	+	+	+	+	+
CEC2017-F19	+	+	+	+	+	+	+	+	+	+	+
CEC2017-F20	+	+	=	+	+	+	+	+	+	=	+
CEC2017-F21	+	+	+	+	+	+	+	+	+	=	=
CEC2017-F22	=	+	+	+	+	+	+	+	+	+	+
CEC2017-F23	+	+	+	+	+	+	+	+	+	+	=
CEC2017-F24	=	+	+	+	+	+	+	=	+	=	=
CEC2017-F25	=	+	−	+	+	+	+	+	+	=	=
CEC2017-F26	+	+	+	+	+	+	−	+	=	+	+
CEC2017-F27	=	+	−	+	+	+	+	+	+	−	=
CEC2017-F28	+	+	+	+	+	+	+	+	+	+	+
CEC2017-F29	+	+	+	+	+	+	+	+	+	+	=
CEC2017-F30	−	+	+	+	+	+	+	+	+	+	=

**Table A7:** Detailed results of the Wilcoxon signed-rank test on CEC2017 (D = 30).

MSCPO vs.	CPO	GA	DE	WOA	HHO	DBO	GJA	SSA	BBO	LSHADE	LSHADE-SPACMA
CEC2017-F1	=	+	+	+	+	+	+	=	+	−	+
CEC2017-F3	+	+	+	+	+	+	=	+	−	+	=
CEC2017-F4	+	+	+	+	+	+	+	+	+	+	+
CEC2017-F5	+	+	+	+	+	+	−	+	−	+	−
CEC2017-F6	+	+	=	+	+	+	+	+	+	−	+
CEC2017-F7	+	+	+	+	+	+	−	+	=	+	−
CEC2017-F8	+	+	+	+	+	+	−	+	−	+	−
CEC2017-F9	+	+	+	+	+	+	+	+	+	−	+
CEC2017-F10	=	+	+	=	−	+	−	−	−	+	−
CEC2017-F11	+	+	+	+	+	+	=	+	+	+	+
CEC2017-F12	+	+	+	+	+	+	+	+	+	+	=
CEC2017-F13	=	+	+	+	+	+	+	+	+	+	=
CEC2017-F14	+	+	+	+	+	+	+	+	+	+	+
CEC2017-F15	+	+	+	+	+	+	+	+	+	+	+
CEC2017-F16	=	+	+	+	+	+	=	+	=	+	−
CEC2017-F17	+	+	+	+	+	+	+	+	+	+	+
CEC2017-F18	+	+	+	+	+	+	+	+	+	+	+
CEC2017-F19	+	+	+	+	+	+	+	+	+	+	+
CEC2017-F20	=	+	+	+	+	+	+	+	+	+	=
CEC2017-F21	=	+	+	+	+	+	=	+	+	+	=
CEC2017-F22	=	+	=	=	−	+	−	−	−	+	−
CEC2017-F23	+	+	+	+	+	+	−	+	=	+	−
CEC2017-F24	=	+	+	+	+	+	−	+	=	+	−
CEC2017-F25	+	+	+	+	+	+	+	+	+	−	−
CEC2017-F26	=	+	+	+	+	+	=	+	=	+	=
CEC2017-F27	+	+	=	+	+	+	+	+	+	=	+
CEC2017-F28	+	+	+	+	+	+	+	+	+	=	=
CEC2017-F29	+	+	+	+	+	+	+	+	+	+	=
CEC2017-F30	+	+	+	+	+	+	+	+	+	+	+

**Table A8:** Detailed results of the Wilcoxon signed-rank test on CEC2017 (D = 50).

MSCPO vs.	CPO	GA	DE	WOA	HHO	DBO	GJA	SSA	BBO	LSHADE	LSHADE-SPACMA
CEC2017-F1	+	+	+	+	+	+	+	+	+	=	+
CEC2017-F3	+	+	+	+	+	+	+	+	−	+	=
CEC2017-F4	+	+	+	+	+	+	+	=	=	=	=
CEC2017-F5	+	+	+	+	+	+	−	+	−	+	−
CEC2017-F6	+	+	+	+	+	+	+	+	+	−	+
CEC2017-F7	+	+	+	+	+	+	−	+	−	=	−
CEC2017-F8	+	+	+	+	+	+	−	+	−	+	−
CEC2017-F9	+	+	+	+	+	+	=	+	+	−	+
CEC2017-F10	=	+	+	=	−	+	−	−	−	+	−
CEC2017-F11	+	+	+	+	+	+	−	+	−	+	=
CEC2017-F12	+	+	+	+	+	+	+	+	+	=	+
CEC2017-F13	=	+	+	+	+	+	+	+	+	+	+
CEC2017-F14	+	+	+	+	+	+	+	+	+	+	=
CEC2017-F15	+	+	+	+	+	+	+	+	+	+	+
CEC2017-F16	+	+	+	+	+	+	=	+	=	+	=
CEC2017-F17	=	+	+	+	+	+	+	+	+	+	+
CEC2017-F18	+	+	+	+	+	+	+	+	+	+	=
CEC2017-F19	+	+	+	+	+	+	+	=	+	+	=
CEC2017-F20	=	+	+	+	+	+	−	+	=	+	=
CEC2017-F21	=	+	+	+	+	+	−	+	=	+	−
CEC2017-F22	=	+	+	=	−	+	−	−	−	+	−
CEC2017-F23	+	+	+	+	+	+	−	+	−	+	−
CEC2017-F24	+	+	+	+	+	+	=	+	=	+	−
CEC2017-F25	+	+	+	+	+	+	+	+	=	=	=
CEC2017-F26	+	+	+	+	+	+	−	+	=	+	=
CEC2017-F27	+	+	+	+	+	+	+	+	=	=	+
CEC2017-F28	+	+	+	+	+	+	+	=	−	−	=
CEC2017-F29	+	+	+	+	+	+	+	+	+	+	=
CEC2017-F30	+	+	+	+	+	+	+	=	+	−	+

**Table A9:** Detailed results of the Wilcoxon signed-rank test on CEC2017 (D = 100).

MSCPO vs.	CPO	GA	DE	WOA	HHO	DBO	GJA	SSA	BBO	LSHADE	LSHADE-SPACMA
CEC2017-F1	+	+	+	+	+	+	−	−	−	−	+
CEC2017-F3	+	+	+	+	+	+	+	+	+	+	+
CEC2017-F4	+	+	+	+	+	+	=	=	−	−	=
CEC2017-F5	+	+	+	+	+	+	−	=	−	=	−
CEC2017-F6	+	+	+	+	+	+	+	+	+	−	=
CEC2017-F7	+	+	+	+	+	+	−	+	−	=	=
CEC2017-F8	+	+	+	+	+	+	−	+	−	=	−
CEC2017-F9	+	+	+	+	+	+	−	−	−	−	−
CEC2017-F10	=	+	+	−	−	+	−	−	−	+	−
CEC2017-F11	+	+	+	+	+	+	−	+	−	+	+
CEC2017-F12	+	+	+	+	+	+	+	+	+	−	+
CEC2017-F13	+	+	+	+	+	+	+	+	+	=	+
CEC2017-F14	+	+	+	+	+	+	+	+	+	+	=
CEC2017-F15	=	+	+	+	+	+	+	+	+	=	+
CEC2017-F16	+	+	+	+	+	+	−	−	−	+	−
CEC2017-F17	+	+	+	+	+	+	=	+	−	+	−
CEC2017-F18	+	+	+	+	+	+	+	+	+	+	−
CEC2017-F19	=	+	+	+	+	+	+	+	+	+	+
CEC2017-F20	=	+	+	+	−	+	−	−	−	+	−
CEC2017-F21	+	+	+	+	+	+	−	+	−	+	−
CEC2017-F22	=	+	+	−	−	=	−	−	−	=	−
CEC2017-F23	+	+	+	+	+	+	−	+	−	+	−
CEC2017-F24	+	+	+	+	+	+	=	+	−	=	−
CEC2017-F25	+	+	+	+	+	+	+	=	−	−	=
CEC2017-F26	+	+	+	+	+	+	=	+	=	=	=
CEC2017-F27	+	+	+	+	+	+	=	+	−	−	+
CEC2017-F28	+	+	+	+	+	+	−	−	−	−	−
CEC2017-F29	+	+	+	+	+	+	=	=	=	+	=
CEC2017-F30	+	+	+	+	+	+	+	+	+	−	+

**Table A10:** Error statistics of different algorithms on CEC2022 (D = 10).

ID	Metric	MSCPO	CPO	GA	DE	WOA	HHO	DBO	GJA	SSA	BBO	LSHADE	LSHADE-SPACMA
CEC2022-F1	Avg	**3.0000 × 10^2^**	3.0000 × 10^2^	3.0001 × 10^2^	3.1396 × 10^2^	4.1790 × 10^4^	2.9111 × 10^3^	3.5281 × 10^3^	3.0000 × 10^2^	3.0012 × 10^2^	1.9439 × 10^4^	**3.0000 × 10^2^**	1.8296 × 10^3^
	Std	1.0556 × 10^−14^	9.4383 × 10^−3^	2.4894 × 10^−2^	2.6545 × 10^1^	1.3780 × 10^4^	1.3046 × 10^3^	1.2199 × 10^3^	4.1977 × 10^−4^	3.0059 × 10^−2^	1.0688 × 10^4^	**0.0000 × 10^0^**	3.4982 × 10^3^
CEC2022-F2	Avg	4.0126 × 10^2^	**4.0098 × 10^2^**	4.1909 × 10^2^	4.2709 × 10^2^	4.5788 × 10^2^	4.0793 × 10^2^	5.6525 × 10^2^	4.0540 × 10^2^	4.0888 × 10^2^	4.4924 × 10^2^	4.0580 × 10^2^	4.0546 × 10^2^
	Std	2.5658 × 10^0^	2.1326 × 10^0^	2.6716 × 10^1^	3.3995 × 10^1^	2.2813 × 10^1^	**7.6889 × 10^−1^**	1.0179 × 10^2^	1.7888 × 10^1^	2.1179 × 10^1^	6.5876 × 10^1^	3.2456 × 10^0^	3.3753 × 10^0^
CEC2022-F3	Avg	**6.0000 × 10^2^**	6.0000 × 10^2^	6.1368 × 10^2^	6.3280 × 10^2^	6.6330 × 10^2^	**6.0000 × 10^2^**	6.2379 × 10^2^	6.0016 × 10^2^	6.0032 × 10^2^	6.4358 × 10^2^	**6.0000 × 10^2^**	6.0000 × 10^2^
	Std	5.2850 × 10^−9^	3.0241 × 10^−7^	1.1658 × 10^1^	1.2668 × 10^1^	1.0243 × 10^1^	1.0970 × 10^−13^	5.6861 × 10^0^	3.9650 × 10^−1^	2.1184 × 10^−1^	1.5115 × 10^1^	**4.7206 × 10^−14^**	1.2762 × 10^−2^
CEC2022-F4	Avg	8.0792 × 10^2^	8.1002 × 10^2^	8.3363 × 10^2^	8.2629 × 10^2^	8.6980 × 10^2^	8.2078 × 10^2^	8.3430 × 10^2^	8.1342 × 10^2^	8.0869 × 10^2^	8.3726 × 10^2^	8.1298 × 10^2^	**8.0519 × 10^2^**
	Std	3.0658 × 10^0^	3.8922 × 10^0^	9.9919 × 10^0^	8.1727 × 10^0^	1.2701 × 10^1^	4.2988 × 10^0^	5.1417 × 10^0^	6.3463 × 10^0^	3.5076 × 10^0^	1.3366 × 10^1^	3.7020 × 10^0^	**2.1808 × 10^0^**
CEC2022-F5	Avg	**9.0000 × 10^2^**	**9.0000 × 10^2^**	1.4431 × 10^3^	1.3815 × 10^3^	9.2504 × 10^2^	9.0001 × 10^2^	1.0836 × 10^3^	9.0032 × 10^2^	9.0017 × 10^2^	1.6221 × 10^3^	**9.0000 × 10^2^**	9.0013 × 10^2^
	Std	**0.0000 × 10^0^**	1.0622 × 10^−10^	1.0555 × 10^2^	1.6307 × 10^2^	7.7149 × 10^0^	1.0565 × 10^−2^	7.5417 × 10^1^	5.7824 × 10^−1^	3.7403 × 10^−1^	4.2522 × 10^2^	**0.0000 × 10^0^**	2.5466 × 10^−1^
CEC2022-F6	Avg	**1.8007 × 10^3^**	1.8038 × 10^3^	3.5586 × 10^3^	5.1615 × 10^3^	3.7776 × 10^3^	5.2438 × 10^3^	3.7702 × 10^5^	2.7387 × 10^3^	3.6305 × 10^3^	3.9181 × 10^3^	1.8082 × 10^3^	1.8176 × 10^3^
	Std	**7.7366 × 10^−1^**	2.0591 × 10^0^	1.9423 × 10^3^	3.1883 × 10^3^	1.6342 × 10^3^	2.7402 × 10^3^	9.2769 × 10^5^	1.1415 × 10^3^	1.6861 × 10^3^	1.7658 × 10^3^	8.0712 × 10^0^	1.6553 × 10^1^
CEC2022-F7	Avg	**2.0006 × 10^3^**	2.0006 × 10^3^	2.0451 × 10^3^	2.0672 × 10^3^	2.0880 × 10^3^	2.0067 × 10^3^	2.0577 × 10^3^	2.0218 × 10^3^	2.0154 × 10^3^	2.0783 × 10^3^	2.0033 × 10^3^	2.0051 × 10^3^
	Std	**6.2531 × 10^−1^**	8.1963 × 10^−1^	4.2407 × 10^1^	2.9243 × 10^1^	2.6968 × 10^1^	6.3848 × 10^0^	1.3924 × 10^1^	8.2830 × 10^0^	9.3245 × 10^0^	3.0865 × 10^1^	6.8814 × 10^0^	7.5346 × 10^0^
CEC2022-F8	Avg	2.2099 × 10^3^	**2.2058 × 10^3^**	2.2422 × 10^3^	2.2368 × 10^3^	2.2622 × 10^3^	2.2187 × 10^3^	2.2298 × 10^3^	2.2208 × 10^3^	2.2237 × 10^3^	2.2363 × 10^3^	2.2117 × 10^3^	2.2168 × 10^3^
	Std	8.9399 × 10^0^	6.1993 × 10^0^	4.4113 × 10^1^	1.4962 × 10^1^	4.0554 × 10^1^	4.1268 × 10^0^	3.3831 × 10^0^	**3.3324 × 10^0^**	2.3062 × 10^1^	1.1594 × 10^1^	8.8353 × 10^0^	7.2909 × 10^0^
CEC2022-F9	Avg	**2.5293 × 10^3^**	2.5293 × 10^3^	2.5293 × 10^3^	2.5774 × 10^3^	2.6565 × 10^3^	2.5293 × 10^3^	2.6351 × 10^3^	2.5293 × 10^3^	2.5293 × 10^3^	2.5829 × 10^3^	2.5293 × 10^3^	2.5293 × 10^3^
	Std	5.6014 × 10^−13^	7.3384 × 10^−8^	1.0151 × 10^−1^	4.4085 × 10^1^	4.3490 × 10^1^	**0.0000 × 10^0^**	5.3496 × 10^1^	6.8443 × 10^−7^	3.9917 × 10^−3^	4.6158 × 10^1^	**0.0000 × 10^0^**	**0.0000 × 10^0^**
CEC2022-F10	Avg	2.5040 × 10^3^	2.5074 × 10^3^	2.6100 × 10^3^	2.5970 × 10^3^	2.6882 × 10^3^	**2.5000 × 10^3^**	2.5096 × 10^3^	2.5648 × 10^3^	2.5590 × 10^3^	2.6830 × 10^3^	2.5296 × 10^3^	2.5003 × 10^3^
	Std	1.9908 × 10^1^	2.6782 × 10^1^	1.4289 × 10^2^	1.0226 × 10^2^	4.1556 × 10^2^	2.8857 × 10^1^	2.4456 × 10^1^	5.7508 × 10^1^	5.5898 × 10^1^	2.8700 × 10^2^	4.9468 × 10^1^	**8.7521 × 10^−2^**
CEC2022-F11	Avg	2.8700 × 10^3^	**2.6433 × 10^3^**	2.8765 × 10^3^	2.8178 × 10^3^	3.2285 × 10^3^	2.8888 × 10^3^	2.8508 × 10^3^	2.8506 × 10^3^	2.9145 × 10^3^	2.9693 × 10^3^	2.8933 × 10^3^	2.8824 × 10^3^
	Std	9.1539 × 10^1^	1.1351 × 10^2^	8.0193 × 10^1^	1.1755 × 10^2^	4.4235 × 10^2^	**3.7193 × 10^1^**	9.1627 × 10^1^	1.1387 × 10^2^	8.3446 × 10^1^	2.6875 × 10^2^	5.8329 × 10^1^	1.1723 × 10^2^
CEC2022-F12	Avg	2.8645 × 10^3^	2.8647 × 10^3^	2.8751 × 10^3^	2.9138 × 10^3^	2.9736 × 10^3^	**2.8614 × 10^3^**	2.8839 × 10^3^	2.8656 × 10^3^	2.8654 × 10^3^	2.9007 × 10^3^	2.8634 × 10^3^	2.8645 × 10^3^
	Std	8.5176 × 10^−1^	**8.3475 × 10^−1^**	2.3293 × 10^1^	5.6427 × 10^1^	4.6002 × 10^1^	1.2919 × 10^0^	1.5831 × 10^1^	1.5071 × 10^0^	1.2106 × 10^0^	3.6772 × 10^1^	1.3339 × 10^0^	1.2529 × 10^0^

Bold values indicate the best result.

**Table A11:** Error statistics of different algorithms on CEC2022 (D = 20).

ID	Metric	MSCPO	CPO	GA	DE	WOA	HHO	DBO	GJA	SSA	BBO	LSHADE	LSHADE-SPACMA
CEC2022-F1	Avg	3.2227 × 10^2^	4.1083 × 10^3^	8.5854 × 10^4^	3.4597 × 10^4^	2.4776 × 10^4^	9.0873 × 10^3^	2.7225 × 10^4^	3.0980 × 10^2^	7.2206 × 10^3^	**3.0029 × 10^2^**	2.4391 × 10^3^	1.0718 × 10^4^
	Std	2.8530 × 10^1^	1.8467 × 10^3^	2.4318 × 10^4^	6.4895 × 10^3^	7.3915 × 10^3^	4.0540 × 10^3^	6.5260 × 10^3^	1.8252 × 10^1^	5.9058 × 10^3^	**2.5899 × 10^−1^**	1.3445 × 10^3^	1.5389 × 10^4^
CEC2022-F2	Avg	4.5267 × 10^2^	4.5520 × 10^2^	5.8842 × 10^2^	4.4962 × 10^2^	5.8895 × 10^2^	5.0299 × 10^2^	9.6322 × 10^2^	4.5679 × 10^2^	4.5223 × 10^2^	4.5258 × 10^2^	4.5088 × 10^2^	**4.3625 × 10^2^**
	Std	1.3686 × 10^1^	1.4748 × 10^1^	5.1452 × 10^1^	**3.8123 × 10^0^**	5.5302 × 10^1^	4.1197 × 10^1^	1.5776 × 10^2^	1.2871 × 10^1^	1.7437 × 10^1^	1.6091 × 10^1^	1.2294 × 10^1^	2.0080 × 10^1^
CEC2022-F3	Avg	6.0000 × 10^2^	6.0000 × 10^2^	7.0530 × 10^2^	6.0000 × 10^2^	6.6627 × 10^2^	6.6026 × 10^2^	6.5689 × 10^2^	6.0304 × 10^2^	6.3982 × 10^2^	6.0287 × 10^2^	**6.0000 × 10^2^**	6.0031 × 10^2^
	Std	4.9401 × 10^−5^	9.0930 × 10^−4^	1.4970 × 10^1^	**2.1979 × 10^−5^**	9.3450 × 10^0^	8.2508 × 10^0^	7.5777 × 10^0^	2.1173 × 10^0^	1.4054 × 10^1^	4.6658 × 10^0^	1.1599 × 10^−4^	2.7839 × 10^−1^
CEC2022-F4	Avg	8.4041 × 10^2^	8.6515 × 10^2^	1.0294 × 10^3^	9.1264 × 10^2^	9.2964 × 10^2^	8.8689 × 10^2^	9.3031 × 10^2^	8.4062 × 10^2^	8.9022 × 10^2^	8.4251 × 10^2^	8.7498 × 10^2^	**8.2178 × 10^2^**
	Std	1.7267 × 10^1^	1.4722 × 10^1^	3.4391 × 10^1^	**6.3304 × 10^0^**	3.9372 × 10^1^	1.3494 × 10^1^	1.4562 × 10^1^	1.2239 × 10^1^	1.9973 × 10^1^	1.5628 × 10^1^	1.0952 × 10^1^	6.8461 × 10^0^
CEC2022-F5	Avg	9.0068 × 10^2^	9.0396 × 10^2^	1.3985 × 10^3^	9.7819 × 10^2^	4.1318 × 10^3^	2.9116 × 10^3^	2.6806 × 10^3^	9.1221 × 10^2^	2.4275 × 10^3^	1.0213 × 10^3^	**9.0014 × 10^2^**	9.2143 × 10^2^
	Std	9.2327 × 10^−1^	1.7783 × 10^1^	6.4628 × 10^2^	5.1807 × 10^1^	1.6955 × 10^3^	2.6748 × 10^2^	2.8505 × 10^2^	1.0033 × 10^1^	6.7230 × 10^1^	1.4267 × 10^2^	**2.3435 × 10^−1^**	2.4905 × 10^1^
CEC2022-F6	Avg	2.2095 × 10^3^	4.3648 × 10^3^	2.3763 × 10^4^	2.4521 × 10^6^	9.3182 × 10^5^	1.6664 × 10^5^	4.8946 × 10^7^	2.6516 × 10^4^	7.1037 × 10^3^	4.3543 × 10^3^	1.8881 × 10^4^	**2.0066 × 10^3^**
	Std	5.5204 × 10^2^	2.1664 × 10^3^	1.9890 × 10^4^	1.6279 × 10^6^	2.7387 × 10^6^	7.1147 × 10^4^	5.5336 × 10^7^	8.1657 × 10^3^	6.1376 × 10^3^	2.8197 × 10^3^	1.4442 × 10^4^	**1.6498 × 10^2^**
CEC2022-F7	Avg	2.0383 × 10^3^	2.0407 × 10^3^	2.2631 × 10^3^	2.0476 × 10^3^	2.2243 × 10^3^	2.1868 × 10^3^	2.1601 × 10^3^	2.0530 × 10^3^	2.1459 × 10^3^	2.0632 × 10^3^	2.0463 × 10^3^	**2.0317 × 10^3^**
	Std	5.9851 × 10^0^	6.6625 × 10^0^	7.4054 × 10^1^	7.6928 × 10^0^	6.4803 × 10^1^	6.1328 × 10^1^	2.9431 × 10^1^	2.3244 × 10^1^	8.4465 × 10^1^	2.6170 × 10^1^	6.6562 × 10^0^	**5.4122 × 10^0^**
CEC2022-F8	Avg	2.2274 × 10^3^	2.2277 × 10^3^	2.3411 × 10^3^	2.2287 × 10^3^	2.2900 × 10^3^	2.2786 × 10^3^	2.3398 × 10^3^	2.2685 × 10^3^	2.3000 × 10^3^	2.2543 × 10^3^	2.2332 × 10^3^	**2.2224 × 10^3^**
	Std	1.4089 × 10^0^	1.3656 × 10^0^	8.7556 × 10^1^	**1.1251 × 10^0^**	5.8427 × 10^1^	8.0489 × 10^1^	1.0216 × 10^2^	6.0135 × 10^1^	8.7268 × 10^1^	5.3719 × 10^1^	3.6922 × 10^0^	3.3623 × 10^0^
CEC2022-F9	Avg	2.4808 × 10^3^	2.4808 × 10^3^	2.6650 × 10^3^	2.4808 × 10^3^	2.5597 × 10^3^	2.4997 × 10^3^	2.6240 × 10^3^	2.4811 × 10^3^	2.4808 × 10^3^	2.4809 × 10^3^	**2.4808 × 10^3^**	2.4808 × 10^3^
	Std	1.3284 × 10^−5^	7.0012 × 10^−3^	4.8565 × 10^1^	1.4915 × 10^−4^	5.2106 × 10^1^	1.5454 × 10^1^	6.1279 × 10^1^	2.4580 × 10^−1^	2.0955 × 10^−2^	1.2294 × 10^−1^	**8.4444 × 10^−14^**	1.0782 × 10^−7^
CEC2022-F10	Avg	2.5159 × 10^3^	2.5049 × 10^3^	5.5443 × 10^3^	2.5183 × 10^3^	4.8477 × 10^3^	3.8960 × 10^3^	2.5570 × 10^3^	2.6615 × 10^3^	3.9993 × 10^3^	2.8724 × 10^3^	2.7069 × 10^3^	**2.5042 × 10^3^**
	Std	4.0088 × 10^1^	2.9491 × 10^1^	1.4316 × 10^3^	2.9033 × 10^1^	1.0301 × 10^3^	8.6680 × 10^2^	**1.6216 × 10^1^**	1.6642 × 10^2^	9.1500 × 10^2^	5.3073 × 10^2^	4.5948 × 10^2^	4.3441 × 10^1^
CEC2022-F11	Avg	2.9000 × 10^3^	2.9100 × 10^3^	6.5796 × 10^3^	2.9000 × 10^3^	3.4629 × 10^3^	3.1610 × 10^3^	6.4783 × 10^3^	3.0047 × 10^3^	2.9233 × 10^3^	2.9061 × 10^3^	**2.9000 × 10^3^**	2.9008 × 10^3^
	Std	5.5255 × 10^−7^	3.0515 × 10^1^	1.2464 × 10^3^	5.0947 × 10^−6^	2.7719 × 10^2^	7.4123 × 10^2^	4.0670 × 10^2^	1.0317 × 10^1^	7.7385 × 10^1^	5.4364 × 10^0^	**1.8882 × 10^−13^**	3.8306 × 10^0^
CEC2022-F12	Avg	2.9465 × 10^3^	2.9604 × 10^3^	3.5174 × 10^3^	**2.9428 × 10^3^**	3.0638 × 10^3^	3.1571 × 10^3^	3.1621 × 10^3^	2.9684 × 10^3^	3.0173 × 10^3^	2.9613 × 10^3^	2.9473 × 10^3^	2.9527 × 10^3^
	Std	6.0775 × 10^0^	7.4974 × 10^0^	1.6128 × 10^2^	**2.8884 × 10^0^**	8.1727 × 10^1^	1.7807 × 10^2^	5.7753 × 10^1^	1.7443 × 10^1^	5.5027 × 10^1^	2.0146 × 10^1^	9.4921 × 10^0^	1.6655 × 10^1^

Bold values indicate the best result.

**Table A12:** Detailed results of the Wilcoxon signed-rank test on CEC2022 (D = 10).

MSCPO vs.	CPO	GA	DE	WOA	HHO	DBO	GJA	SSA	BBO	LSHADE	LSHADE-SPACMA
CEC2022-F1	+	+	+	+	+	+	+	+	+	=	+
CEC2022-F2	−	+	+	+	+	+	+	+	=	+	+
CEC2022-F3	+	+	−	+	+	+	+	+	+	−	+
CEC2022-F4	+	+	+	+	+	+	=	+	+	+	−
CEC2022-F5	+	+	+	+	+	+	+	+	+	=	+
CEC2022-F6	+	+	+	+	+	+	+	+	+	+	+
CEC2022-F7	=	+	+	+	+	+	+	+	+	=	+
CEC2022-F8	=	+	+	+	+	+	+	+	+	=	+
CEC2022-F9	+	+	=	+	+	+	+	=	+	=	=
CEC2022-F10	=	+	−	+	+	+	+	+	+	=	=
CEC2022-F11	−	+	=	+	=	=	+	+	−	=	=
CEC2022-F12	=	+	−	+	+	+	+	+	+	−	=

**Table A13:** Detailed results of the Wilcoxon signed-rank test on CEC2022 (D = 20).

MSCPO vs.	CPO	GA	DE	WOA	HHO	DBO	GJA	SSA	BBO	LSHADE	LSHADE-SPACMA
CEC2022-F1	+	+	+	+	+	+	−	+	−	+	=
CEC2022-F2	=	+	=	+	+	+	+	=	=	=	−
CEC2022-F3	+	+	−	+	+	+	+	+	+	−	+
CEC2022-F4	+	+	+	+	+	+	=	+	=	+	−
CEC2022-F5	=	+	+	+	+	+	+	+	+	−	+
CEC2022-F6	+	+	+	+	+	+	+	+	+	+	=
CEC2022-F7	=	+	+	+	+	+	+	+	+	+	−
CEC2022-F8	=	+	+	+	+	+	=	+	=	+	−
CEC2022-F9	+	+	+	+	+	+	+	+	+	−	−
CEC2022-F10	=	+	+	+	+	+	+	+	+	=	=
CEC2022-F11	+	+	+	+	+	+	+	+	+	−	+
CEC2022-F12	+	+	−	+	+	+	+	+	+	=	=
